# Market Integration and Price Dynamics under Market Shocks in European Union Internal and External Cheese Export Markets

**DOI:** 10.3390/foods11050692

**Published:** 2022-02-26

**Authors:** Huidan Xue, Liming Wang, Chenguang Li

**Affiliations:** 1 School of Economics and Management, Beijing University of Technology, Beijing 100124, China; liming.wang@ucd.ie; 2 Irish Institute for Chinese Studies, University College Dublin, Belfield, Dublin 4, D04 V1W8 Dublin, Ireland; 3 School of Agriculture and Food Science, University College Dublin, Belfield, Dublin 4, D04 V1W8 Dublin, Ireland; chenguang.li@ucd.ie

**Keywords:** global vector autoregressive model, GVAR, price transmission, market integration, uncertainties, time series

## Abstract

The dairy sector in the European Union (EU) has experienced policy changes and market shocks recently. Using the global vector autoregressive (GVAR) model, this paper explores regional market integration, the feedback between market shocks and price dynamics, and the link between EU’s cheese export markets and energy market. This paper assesses and compares which influencing factors are typically associated with intra-EU and extra-EU cheese export price movement with regards to shocks to crude oil price, farm-gate raw milk price, and consumer price index (CPI) for food and cheese production of six representative EU member states, respectively. Using generalized impulse response functions, this paper finds that EU’s internal cheese export market is not well integrated, while EU’s external market is well integrated, with France as an exception. It also finds that the external cheese export market is vulnerable to shocks from the energy market compared to the internal market. Raw milk prices from the upstream supply chain have strong spill-over effects on EU’s internal cheese export market, yet their impact on extra-EU cheese export prices is relatively less significant. The movement patterns of extra-EU cheese export prices of Ireland and the UK show similar patterns in the long run. It is concluded that the dynamics of cheese export prices in the internal and external markets of the EU are different under market shocks.

## 1. Introduction

European Union (EU) dairy policy is subject to the Common Agricultural Policy (CAP) and one of the most important objectives of the CAP is to facilitate agricultural market integration within individual member states as well as at EU level by reinforcing price discovery mechanisms. The EU is heterogeneous, with 28 member states of different economies, industry structures, and trade patterns before 2020. Article 2 of the Lisbon Treaty states the common ideal of the EU member states is to progress by cooperation, specifically:

“The Union shall establish an internal market. It shall work for the sustainable development of Europe based on balanced economic growth and price stability, a highly competitive social market economy, aiming at full employment and social progress, and a high level of protection and improvement of the quality of the environment.

…

It shall promote economic, social and territorial cohesion, and solidarity among Member States”.

As seen in Article 2, the EU promotes internal market, economic, and social cohesion and designs various policies to achieve this goal. In terms of the dairy sector, CAP has undergone several rounds of reforms to implement a more market-oriented policy for enhancing competitiveness of the EU dairy sector in the international market. These policy and market changes have resulted in market-oriented competition and interactions among member states of the EU and structural changes for the EU dairy sector in international trade [1]. However, changes to EU dairy policies such as the abolition of milk production quota and removal of price floors have also led to price fluctuation and market unpredictability [2]. The most fundamental policy change in the dairy sector was the abolition of the milk quota in March 2015 which released constraint on milk production in the northwestern EU member states [3]. EU CAP reform and the outcome of WTO trade negotiations to lower border protection from imports and reduce subsidized exports make it clear that the EU dairy sector will be more market oriented in future. Besides CAP reforms, the EU dairy sector is experiencing market shocks, such as uncertainties brought on by Brexit, rising trade protectionism, and a slowdown in global economic growth, which could impact the equilibrium of EU dairy markets and trade patterns. Therefore, it is necessary to have a comprehensive understanding of EU dairy price transmission and market dynamics for the intra-EU and extra-EU trade market.

Many studies have conducted research on market integration in the EU at the regional level [1,3,4,5,6], or spatial price transmission of only one single dairy product’s prices [7,8,9,10], or studied the effects of changes on the dairy sector at the national level for a single-country case [11]. However, there are few studies that focus on the effect of influencing factors on the dairy sector across the EU or comprehensive studies on the degree of market integration in terms of dairy trade for the EU. Therefore, a comprehensive analysis to reveal the interactions and heterogeneity across EU member states can provide insightful information to facilitate industry development, enhance competitiveness, and ensure price stability in the EU dairy sector.

The dairy sector is a multi-product, high-energy-consumption, and dynamic industry with its products varying in terms of storability, market characteristics, and price stability. Thus, it is necessary to conduct analysis at the disaggregated product level to better understand the market dynamics and price mechanisms for each specific product. In this study, cheese is selected as a representative dairy product to analyze spatial price transmission and market dynamics in the EU for the following reasons: (1) The EU is the world’s biggest cheese exporter and is a producer with an enormous number of cheese varieties exported all over the world. (2) Cheese, as one of the major dairy products mainly produced and exported in developed countries, has more potential for future market expansion to other emerging markets. Understanding price transmission and price dynamics mechanisms for cheese could shed light on the adjustment of trade strategy and policy to boost the dairy industry as a whole. (3) Cheese in the EU has the unique characteristic that its export market is dominated by a few major European exporting countries. (4) Cheeses are one of the major dairy products with great nutritional and biological value, produced through the coagulation of milk protein (casein) separated from the milk’s whey, and many different varieties are traded and marketed around the world. It is an important dairy product and its price transmission could reflect the effects of marketing, branding, and geographical protection. This indicates useful implications on policy prescription to ensure market and price stability for dairy products. Among 28 EU member states, Germany, the Netherlands, France, Italy, Ireland, and the United Kingdom (UK) are selected as the countries to study for the following reasons: (1) Germany, the Netherlands, France, and Italy are the top four European cheese exporters, accounting for almost half of EU total cheese exports. (2) Cheeses are the most exported and imported dairy product between the EU-27 and the UK. Ireland and the UK have very close trade relations in terms of cheese. Incorporating these two countries into the analysis could provide insights on the possible consequences of Brexit on trade relations between the UK and other EU member states. Brexit could incur potential policy shocks for the EU, especially for Ireland, and this is one of the main areas of interest for policy investigation in this study.

This study concentrates on the EU dairy market with a special focus on cheese exports and investigates whether an internal export market with strong market integration for cheese exists in the EU and whether price transmission is smooth in non-EU (external) export markets. It also examines the impact of market shocks on the cheese export price of each individual country. In particular, the following questions are investigated and analyzed:

1. To what degree is there market integration among major EU cheese-exporting member states in terms of intra-EU and extra-EU (non-EU) exports, respectively?

2. How do export prices for different countries interact with one another? Are there any differences for intra-EU export price transmission and extra-EU export price transmission?

3. How do various market shocks affect cheese export prices for different countries? Are there any differences in the impacts on intra-EU export prices and extra-EU export prices?

To better understand these questions, the global vector autoregressive model (GVAR) proposed by Pesaran et al. [12] is employed. Compared with other econometric models, such as dynamic equilibrium models and basic vector autoregressive model, the GVAR approach has special advantages: (1) the data requirements are relatively low, while at the same time this high-dimensional model can arrive at rich conclusions via model estimates. (2) It can illustrate the dynamic relationships among studied variables across country, variables, and time spans. (3) It connects country-specific models via several channels of international linkages, deciphering the size and speed of price transmission and shocks from other countries and their domestic markets. (4) It well fits the objectives of this study: it allows for a high-dimension dataset that allows this study to simultaneously incorporate higher country-dimension, time-dimension, and variable-dimension datasets to conduct dynamic analysis of the price transmission and interactions between different countries following market shocks [13].

The structure of the rest of this study is as follows: Section 2 presents an overview of EU’s trade patterns for cheese in the internal and external markets. Section 3 describes the methodology and conceptual framework for this study. Section 4 presents the results of the empirical analysis. Finally, Section 5 reaches the conclusion and Section 6 discusses implications and future research.

## 2. Overview of EU Cheese Sector and Trading Context

### Cheese in the EU

The EU is the largest cheese producer and exporter in the world and accounts for 79% of global cheese export with USD 24.8 billion in export value in 2018 (including both intra-EU and extra-EU exports of the EU-28 member states). The EU is projected to account for 37% of world cheese exports by 2027 and 48% by 2028 (including only extra-EU exports). This export growth will be sustained by increased exports to Canada via the EU-Canada Comprehensive Economic and Trade Agreement, the assumed future ending of the ban imposed by the Russian Federation, and increased exports to Japan following ratification of the bilateral trade agreement in 2019 [14,15]. Many cheeses from the EU member states have been protected under the Rules and Regulations 2081/92 and No. 1804/99: namely, Protected Designation of Origin (PDO), Protected Geographical Indication (PGI), and Traditional Speciality Guaranteed (TSG). These quality labels protect product names against imitation of cheese originating from specific areas, promoting product diversity and creating uniqueness for EU cheese products [16,17].

The production levels of EU cheese have remained stable over the years and consumption to production ratio has exhibited a slight downward trend. In 2018, total production and consumption of cheese amounted to over 9.9 million and 8.8 million tonnes, respectively, with a production to consumption ratio of 88.74% according to OECD-FAO Agricultural Outlook Database (2018–2029). In general, a large share of cheese produced in the EU is domestically consumed either by trade between EU members or domestic consumption within individual EU member states.

Intra-EU trade of cheese is much more pronounced than extra-EU trade in terms of both exports and imports. In 2017, Germany was the largest cheese exporter with an average export value of USD 4.3 billion, which represents 15% of total world export. The most significant export destinations are Italy (21%), the Netherlands (13%), Austria (6.9%), France (6.5%), and Spain (6.1%). The second place belongs to the Netherlands with an average export value of USD 3.80 billion, which represents 13% of total world exports. Its top five export destinations are Germany (32%), Belgium-Luxembourg (13%), France (9.4%), Spain (5.7%), and the United Kingdom (4.3%). They represent almost three-quarters (64.4%) of the Netherlands’ cheese export. France ranks third for cheese exports in trade value, with the average export value hitting USD 3.50 billion, representing 12% of world exports, followed by Italy and Denmark with shares of 9.8% and 5.7%, respectively. In total, the top five cheese export countries have absolute dominance as they account for 55.5% of global cheese export by value. Compared with Germany, the other EU countries including the Netherlands, France, Italy, the United Kingdom, and Ireland have much smaller cheese exports by value and over 50% of Ireland’s cheese export is destined for the United Kingdom.

Intra-industry trade (IIT), which is defined as the concurrent importation and exportation of similar goods [18], has been a common phenomenon in international trade [19]. There is high intra-industry trade in the EU due to taste similarity, product differentiation, and scale economies. Specifically, economic integration and proximity to community and other European markets might lead to deeper intra-industry trade in the EU [20]. Many European countries trade cheese to offer wider varieties of cheeses in their domestic markets so their residents can select and consume whichever types of cheeses they prefer. The intra-industry trade of cheese in the EU satisfies the consumers who prefer variety, choice, and the advantages accruing from competitive pressures [21].

**Figure 1:**
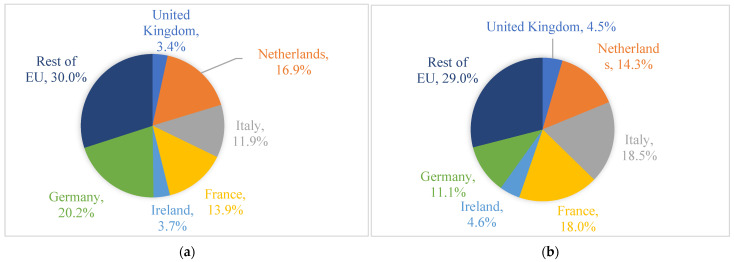
Six studied countries’ cheese export shares in international markets in 2018 (Billion USD). (Source: authors’ depiction using data downloaded from Eurostat database, accessed on 11 August 2019). (**a**) Cheese export shares in the intra-EU market. (**b**) Cheese export shares in the extra-EU market.

Figure 1a,b depict cheese export shares of the six studied countries in the intra-EU and extra-EU markets. Germany contributed the largest share (20.2%) of intra-EU cheese exports, followed by the Netherlands (16.9%), France (13.9%), and Italy (11.9%). In terms of extra-EU cheese export, Italy takes first place in cheese export share, accounting for 18.5% of the EU’s extra-EU cheese exports, followed by France (18.0%), the Netherlands (14.3%), and Germany (11.1%).

**Figure 2:**
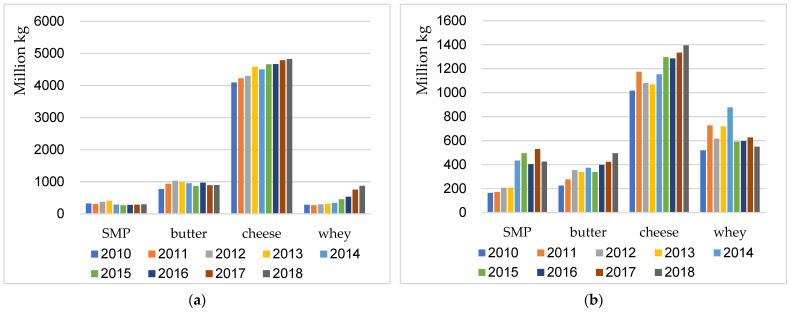
The bilateral export in quantity of major dairy products between EU-27 and the UK. (Source: authors’ depiction using data downloaded from Eurostat database, accessed on 6 September 2019). (**a**) EU-27 export quantity of major dairy products on the UK market. (**b**) UK export quantity of major dairy products on the EU-27 market.

Many stakeholders in the EU dairy sector have concerns over the inevitable Brexit and the corresponding disruption of the EU free trade and single market system. The UK has very close trade relationships with other EU member states in the case of dairy products, especially cheese, as shown in Figure 2a,b. Brexit might have a negative influence on EU market structure, and on the intra-EU and extra-EU dairy trade, especially for Irish dairy exports. Cheese is the most exported dairy product from the other EU countries to the UK as well as from the UK to the other EU countries, with increasing export quantities over the years. The net export of cheese from the EU-27 to the UK market is significant and cheese is of vital importance for both EU-27 member states and the UK.

In summary, the EU is the largest exporter of cheeses globally and its intra-EU cheese exports outweigh its extra-EU cheese exports. Most of the top cheese-exporting countries of the EU are also top cheese importers because different EU member states produce different cheese varieties and customers have preferences for different varieties of cheeses. Various types of cheeses from the EU are traded between EU member states and all over the world. The UK and Ireland are relatively small cheese exporters in the EU, yet the cheese trade between the two countries is vibrant and occurs in large quantities. These two countries could be affected most by Brexit and potential trade policy changes.

**Figure 3:**
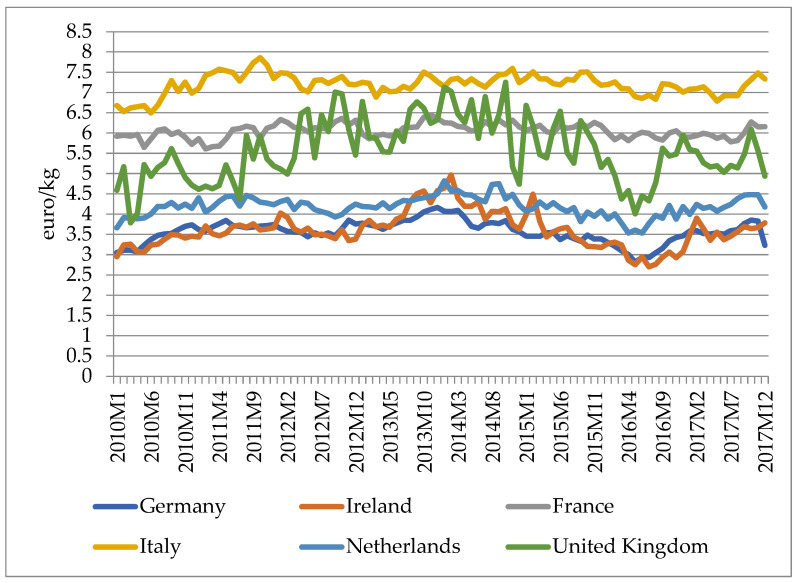
Intra-EU cheese export prices of major EU exporters, January 2010–December 2017. (Source: prices calculated by author using data downloaded from Eurostat Database, HS Classification EU trade since 1988 by HS2,4,6 and CN8 [DS-645593], product code: HS heading 0406–cheeses and curds, accessed in August 2019).

**Figure 4:**
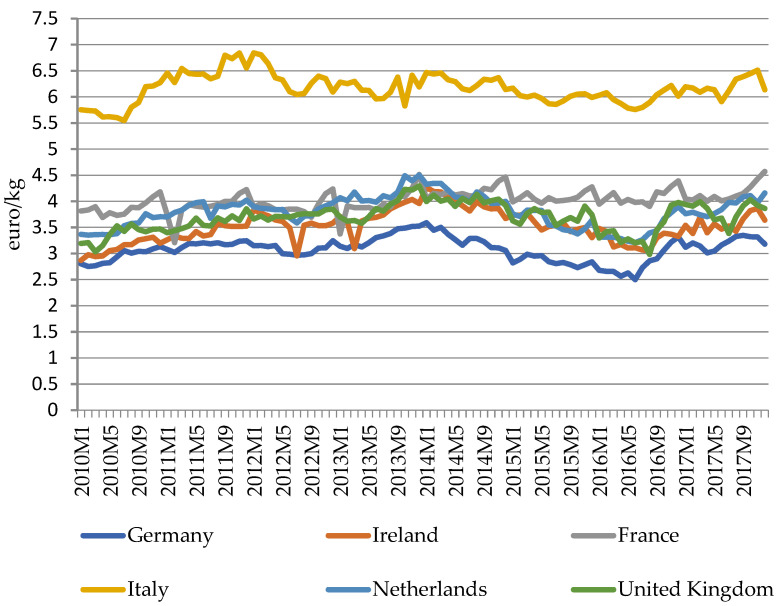
Extra-EU cheese export prices of major EU exporters, January 2010–December 2017. (Source: prices calculated by author using data downloaded from Eurostat Database, HS Classification EU trade since 1988 by HS2,4,6 and CN8 [DS-645593], product code: HS heading 0406–cheeses and curds, accessed in August 2019).

Figure 3 and Figure 4 below depict the studied European countries’ monthly export prices for cheese to extra-EU and intra-EU markets between 2010 and 2017. The free movement and trade among EU member states and the tariff and non-tariff barriers faced by EU member states for non-EU exports result in clear differences between intra-EU and extra-EU export prices for EU cheeses. As indicated in Figure 3 and Figure 4, extra-EU export prices are higher than intra-EU export prices, and Italy has the highest export prices while Germany, as the largest cheese exporter and importer, has had the lowest prices over the years. The cheese prices of all the studied countries displayed common movement patterns but were different at the price level. For extra-EU export prices, price levels and fluctuations of different countries vary greatly: (a) Prices of Italy, France, and the UK are the top three highest, while prices of the Netherlands, Ireland, and Germany are relatively lower and their price series have displayed similar fluctuation patterns over the years; (b) The price series of Italy and France have displayed similar patterns since 2012, while the UK’s price has fluctuated violently over the years and has inflated to a high level during 2012 to 2015. For intra-EU export prices, the price series of all countries display similar fluctuation patterns, while the price level for Italy is the highest and its price fluctuation before 2012 was larger than other countries. The main reason for the price level differences among the studied countries is that their major exported cheese types are different: In 2018, the Netherlands had the largest net export quantity, with Gouda, Edam, and Emmentaler as the main exported cheeses. France is in second place as net exporter of cheeses, with Brie and Camembert as the main exported cheese types. Italy is in third place as a net exporter of cheeses, with Grana Padano and Parmigiano Reggiano as the main exported cheese types. Ireland ranks fourth place as a net exporter, with Cheddar and Jarlsberg as the top net exported products. Compared with other countries, Germany is both the largest cheese exporter and importer in the EU. It is in fifth place as a net exporter in the studied six EU member states. Among various exported and imported cheese types, Gouda and Edam are the top four main exported cheeses, while Gouda, Cheddar, and Edam are the top three imported cheeses in Germany. Different from other countries, the UK is a net importer of cheese and imports Cheddar the most. Other fresh cheese (product code: CN 04061080) and Kefalograviera and Kasseri are the only two categories of cheeses that have positive net exports for the UK.

Russia has imported huge amounts of cheese from the EU. In 2014, Russia banned the import of EU dairy products [22], which greatly harmed EU cheese exports and cheese then ended up on world markets, resulting in a crash in prices. In March 2015, the EU abolished its quotas on milk production which boosted the milk production further, thus causing further price declines. In the same period, New Zealand, Australia, and the United States increased their dairy production. All of these factors created a challenging environment for the EU dairy sector in 2015 [23]. The figures show that export prices of both extra-EU and intra-EU cheese exports declined from early 2014 to mid-2016. The gradual rise of price since mid-2016 is due to development of both the supply and demand sides of the EU dairy sector: (1) The EU removed 351,029 tonnes of skim milk powder from market by public purchases (EU intervention policy); (2) Increases in domestic and international cheese consumption and production reduction in some key producers created stronger demand for EU cheeses [23].

## 3. Methods and Data

In this study, it is assumed that cheese export prices in the EU are driven by shocks to: (a) macroeconomic factors (price index of food); (b) production-side factors (production levels and crude oil price); (c) input prices (farm-gate raw milk price and crude oil price); and (d) competition and price transmission (the cheese export prices of other major exporters). To understand market integration and spatial price transmission of cheese export prices among the EU member states, cheese prices of seven studied countries and the country group are incorporated into the analysis. The spatial price transmission analysis is to test the assumption that the internal EU cheese market is well integrated, while the extra-EU cheese trade market is less integrated than the internal one. Market shocks will be simulated to test and address the assumption that increases in crude oil price, raw milk price, and CPI for food cause increases in cheese export prices and increases in production will decrease cheese export prices.

### 3.1. Methods

The global vector autoregressive (GVAR) model is employed in this paper. The GVAR model has been widely applied in macroeconomics, including (see Chudik and Pesaran [13] for comprehensive surveys) studying the factors and shocks affecting global inflation [24], global imbalance [25,26,27,28], effects of fiscal and monetary policy [29,30,31,32,33,34,35], credit supply shock [36,37,38], spill-overs in the labor market [39], financial market [40], energy market [41], trade [42], and different sectors [43,44,45], etc. So far, the GVAR model has been applied to agricultural markets in a few empirical studies, including analyzing linkages among food commodity prices, energy prices, and financial sectors in the major wheat export countries by Gutierrez et al. [46], studying short-run food price shock propagation in Sub-Saharan Africa (SSA) by Pierre and Kaminski [47], and analyzing spatial price transmission of the global butter export market under different market shocks such as exchange rate fluctuation, shocks to fertilizer prices, palm oil price, and crude oil price by Xue et al. [48].

For comparison, two global vector autoregressive (GVAR) models are separately constructed to analyze the price dynamics and impacts of shocks on intra-EU and on extra-EU export prices. For both the intra-EU GVAR model and extra-EU GVAR model, six VECX models are constructed, respectively, one for each of the main exporters among EU member states: Ireland, the Netherlands, Italy, Germany, France, and the UK. Moreover, the rest of the EU (REU) regional VECX model is specified to represent the effects from all the other EU member states. This study follows a similar approach to Gutierrez, Piras, and Paolo Roggero [46] and Pierre and Kaminski [47] as follows.

The GVAR model is constructed in two steps: the first step is to construct the individual unit models VARX, and the second step is to stack the estimated unit models together to form one large global VAR model.

It is assumed that there are N cross-section units, for each of which k variables are observed during the time period t=1, 2, 3, …, T. *x_it_* denotes the $k_{i}\times 1\;$vector of variables specific to unit i in time period *t*. $x_{t}=\left(x_{1t}^{\prime },x_{2t}^{\prime },\ldots ,\;x_{nt}^{\prime }\right)\prime$ denotes the k×1 vector of all the variables, where $k=\overset{N}{\underset{i=1}{\sum }}k_{i}$. For models in this paper, the cross-section units refer to the specific countries and regions defined (i.e., Ireland, the Netherlands, Italy, Germany, France, and the UK. Moreover, the rest of the EU (REU) model for research purposes is specified.

Country-specific conditional models are estimated separately. These individual country models explain the domestic variables of a given economy, *x_it_*, conditional on country-specific cross-section averages of foreign variables, collected in the $k^{*}\times 1$ vector.

(1) $$x_{it}^{*}={\tilde{W}}_{i}^{\prime }x_{t}$$

for i=1, 2, 3, …, N, where ${\tilde{W}}_{i}$ is$\;k\times k^{*}$ matrix of country-specific weights, typically constructed using data on bilateral foreign trade. Both $k_{i}\;$and $k^{*}$ are treated as small (typically 4 to 6).

*x_it_* is modelled as a VARX model, namely a VAR model augmented by the vector of the “star” variables $x_{it}^{*}$ and their lagged values as Equation (2). The model will be represented as Equation (3) when it is augmented by global variables $d_{t}$ and its lagged values.

(2) $$x_{it}=a_{i0}+a_{i1}t+\overset{p_{i}}{\underset{l=1}{\sum }}\Phi_{il}x_{i,\;t-l}+\Lambda_{i0}x_{it}^{*}+\overset{q_{i}}{\underset{l=1}{\sum }}\Lambda_{il}x_{i,t-l}^{*}+\varepsilon_{it}$$

(3) $$x_{it}=a_{i0}+a_{i1}t+\overset{p_{i}}{\underset{l=1}{\sum }}\Phi_{il}x_{i,\;t-l}+\Lambda_{i0}x_{it}^{*}+\overset{q_{i}}{\underset{l=1}{\sum }}\Lambda_{il}x_{i,t-l}^{*}+\Psi_{i0}d_{t}+\overset{s_{i}}{\underset{l=1}{\sum }}\Psi_{il}d_{t-l}+\varepsilon_{it}$$

for i=1, 2, …, N, where $\Phi_{il}$, for *l* = 1, 2, …, $p_{i}$, $\Lambda_{il}$, for *l* = 0, 1, 2, … $q_{i}$, are $k_{i}\times k_{i}\;$and $k{\;}_{i}\times k^{*}$ matrices of unknown parameters, respectively. Additionally, $\varepsilon_{it}$ are$\;k_{i}\times 1$ error vectors.

For simplicity, it is assumed that $\Psi_{il}=0$ for $l=0,\;1,\;2,\ldots ,s_{i}$ in the following derivation. Therefore, let $Z_{it}={\left(x_{it}^{\prime },\;{x_{it}^{*}}^{\prime }\right)}^{\prime }$ be $k_{i}+k^{*}$ dimensional vector; thus, Equation (2) can be rewritten as:

(4) $$A_{io}{Ζ}_{it}=a_{i0}+a_{i1}t+\overset{p}{\underset{l=1}{\sum }}A_{il}{Ζ}_{i,\;t-l}+\varepsilon_{it}$$

where,

$A_{i0}=\left({Ι}_{ki}-\Lambda_{i0}\right)$, $A_{it}=\left(\Phi_{il},{\;\Lambda }_{il}\right)$ for l=1, 2, …, p.

The first step of the GVAR approach to estimate country models VARX as Equation (3) is completed. VARX allows for cointegration within and across economies (via the star variables).

Then the deduction is made to obtain error-correction representation (8): minus $x_{i,\;t-1}$ on both sides of Equation (2); the result is:

(5) $$\Delta x_{it}=a_{i0}+a_{i1}t+\Lambda_{i0}x_{it}^{*}-\Lambda_{i0}x_{i,t-1}^{*}+\Lambda_{i0}x_{i,t-1}^{*}-x_{i,t-1}+\overset{p_{i}}{\underset{l=1}{\sum }}\Phi_{it}x_{i,t-l}+\overset{q_{i}}{\underset{l=1}{\sum }}\Lambda_{it}x_{i,t-l}^{*}+\varepsilon_{it}$$

Then:

(6) $$\Delta x_{it}=a_{i0}+a_{i1}t+\Lambda_{i0}\Delta x_{it}^{*}+\Lambda_{i0}x_{i,t-1}^{*}-x_{i,t-1}+\overset{p_{i}}{\underset{l=1}{\sum }}\Phi_{it}x_{i,t-l}+\overset{q_{i}}{\underset{l=1}{\sum }}\Lambda_{it}x_{i,t-l}^{*}+\varepsilon_{it}$$

Then:

(7) $$\Delta x_{it}=a_{i0}+a_{i1}t+\Lambda_{i0}\Delta x_{it}^{*}-A_{i0}{Ζ}_{i,t-1}+\overset{p}{\underset{l=1}{\sum }}A_{il}{Ζ}_{i,\;\;t-l}+\varepsilon_{it}$$

Therefore, by rearranging terms, the error-correction representation of Equation (2) as follows is constructed as Equation (8):

(8) $$\Delta x_{it}=a_{i0}+a_{i1}t+\Lambda_{i0}\Delta x_{it}^{*}-\Pi_{i0}{Ζ}_{i,t-1}+\overset{p}{\underset{l=1}{\sum }}H_{il}\Delta {Ζ}_{i,\;t-l}+\varepsilon_{it}$$

where $\Pi_{i0}=-(A_{i0}-\overset{p}{\underset{l=1}{\sum }}A_{il}),$ $H_{il}=-\overset{p-1}{\underset{l=1}{\sum }}A_{i,\;l+1},\;\Delta =1-L$ is the first-order difference.

The second step of the GVAR approach is to stack estimated country models to form one large global VAR model.

Using the $\left(k_{i}+k^{*}\right)\times k$ dimensional “link” matrices $W_{i}\;=\;(E_{i}^{\prime },{\tilde{W}}_{i}^{\prime })$, where $E_{i}$ is $k\times k_{i}$ dimensional selection matrix that selects $x_{it}$, namely $x_{it}={Ε}_{i}^{\prime }x_{t}$, and ${\tilde{W}}_{i}^{\prime }$ is the weight matrix introduced in Equation (1) to define country-specific foreign star variables. Then:

(9) $$Z_{it}={\left(x_{it}^{\prime },\;{x_{it}^{*}}^{\prime }\right)}^{\prime }=W_{i}x_{t}$$

Then Equation (4) can be written as Equation (10):

(10) $$A_{i0}W_{i}x_{t}=a_{i0}+a_{i1}t+\overset{p}{\underset{l=1}{\sum }}A_{il}W_{i}x_{t-l}+\varepsilon_{it}$$

Then Equation (11) is:

(11) $$G_{0}x_{t}=a_{0}+a_{1}t+\overset{p}{\underset{l=1}{\sum }}G_{l}x_{t-l}+\varepsilon_{t}$$

where $\varepsilon_{t}={\left(\varepsilon_{1t}^{\prime },\;\varepsilon_{2t}^{\prime },\ldots ,\varepsilon_{Nt}^{\prime }\right)}^{\prime }$, $a_{0}={\left(a_{10}^{\prime },\;a_{20}^{\prime },\;\ldots ,\;a_{N0}^{\prime }\right)}^{\prime }$, $a_{1}={\left(a_{11}^{\prime },\;a_{21}^{\prime },\;\ldots ,\;a_{N1}^{\prime }\right)}^{\prime }$,

$$G_{l}=\left(\begin{array}{c} \begin{array}{c} \begin{array}{c} A_{1,\;l}W_{1}\\ A_{2,l}W_{2} \end{array}\\ \vdots \end{array}\\ A_{N,l}W_{N} \end{array}\right)\;$$

If matrix $G_{0}\;$is invertible, then by multiplying Equation (6) by $G_{0}^{-1}$ from the left, the solution to the GVAR model is obtained.

(12) $$x_{t}=b_{0}+b_{1}t+\overset{p}{\underset{l=1}{\sum }}F_{l}x_{t-l}+G_{0}^{-1}\varepsilon_{t}$$

where $F_{l}=G_{l}G_{0}^{-1},$ $b_{0}=a_{0}G_{0}^{-1},$ $b_{1}=a_{0}G_{0}^{-1}.$

Equation (12) can be solved recursively and used for analyzing the impulse responses.

In this study, the country-specific variables include: (1) index of extra-EU-28 export prices in EUR as $p_{it}^{e\_ex}$, which is only included in the extra-EU export price GVAR model; (2) index of intra-EU-28 export prices in EUR as $p_{it}^{e\_in}$*,* which is only included in the intra-EU export price GAVR model; (3) Harmonised Index of Consumer Price (HICP) of food, denoted as ${\mathrm{HICPF}}_{it}$*,* which reflects food inflation in each country. (4) Cheese production, $Pro_{it}$, which reflects the supply of cheese in each country. (5) The index of farm-gate prices of raw milk in EUR as $p_{it}^{f}$, which represents the upstream price along the supply chain.

The foreign-specific variables are established as a geometric average of the country-specific variables. The weights are computed as averages of shares of total EU exports from 2010 to 2017. Therefore, the foreign-specific variables include: (1) the average of competitors’ export prices for extra-EU-28 trade, $p_{it}^{e\_ex*}=\underset{i\neq j}{\sum }w_{j}p_{jt}^{e\_ex}$ (extra-EU export price GVAR model only); (2) the average of competitors’ export prices for intra-EU-28 trade, $p_{it}^{e\_in*}=\underset{i\neq j}{\sum }w_{j}p_{jt}^{e\_in}$ (intra-EU export price GVAR model only); (3) the average of the HICP food,$\;{\mathrm{HICP}}_{it}^{*}=\underset{j\neq i}{\sum }w_{j}{\mathrm{HICP}}_{jt}$; (4) the average of the cheese production index, $Pro_{it}^{*}=\underset{j\neq i}{\sum }w_{j}Pro_{jt}$; (5) the average of competitors’ raw milk prices, $p_{it}^{f*}=\underset{i\neq j}{\sum }w_{j}p_{jt}^{f}$.

Global variables can affect the systems of each region and are of vital importance to all countries. The dairy industry is dependent on energy in oil and energy is necessary for the production and transportation of milk and dairy products. Moreover, oil price inflation will indirectly increase feed prices through biofuel and corn price transmission. Therefore, the dairy market can be affected by changes in energy prices such as world crude oil price, $p_{t}^{o}$. In the constructed GVAR model or global vector error-correction model (GVECM), crude oil price is set to be endogenous in the UK VECX model for both intra-EU and extra-EU models. This is because the UK is the major crude oil producer among the 28 member states of the EU [49]. The variable vectors are as follows:

The domestic variable vectors for the intra-EU VECX models:

(13) $$x_{it}={\left(p_{it}^{e_{in}},\;{\mathrm{HICPF}}_{it},Pro_{it},\;p_{it}^{f}\right)}^{\prime }$$

The domestic variable vectors for the extra-EU VECX models:

(14) $$x_{it}={\left(p_{it}^{e_{ex}},\;{\mathrm{HICPF}}_{it},Pro_{it},\;p_{it}^{f}\right)}^{\prime }$$

The foreign variable vectors for the intra-EU VECX models:

(15) $$x_{it}^{*}={\left(p_{it}^{e\_in*},\;{\mathrm{HICP}}_{it}^{*}\;,\;Pro_{it}^{*}\;,\;p_{it}^{f*}\;\right)}^{\prime }$$

The foreign variable vectors for the extra-EU VECX models:

(16) $$x_{it}^{*}={\left(p_{it}^{e\_ex*},\;{\mathrm{HICP}}_{it}^{*}\;,\;Pro_{it}^{*}\;,\;p_{it}^{f*}\;\right)}^{\prime }$$

The global variable vectors for intra-EU and extra-EU VECX models:

(17) $$d_{t}=\left(p_{it}^{o}\right)$$

where for *i* = 1, 2, …, 7, which represents the seven studied EU countries, that is, Ireland, the Netherlands, Italy, Germany, France, the UK, and REU, respectively.

The above-mentioned vectors are put into the model specification for analysis.

### 3.2. Data

The monthly data series from January 2010 to December 2016 is used for the analysis and all the variables are transformed to their indexes using the average value of data from January 2010 to December 2010 as the base year data and then converting the index into natural logarithm forms. The export prices are calculated using the equation: $p_{it}^{h}=\frac{V_{it}^{h}}{Q_{it}^{h}}$, where $V_{it}^{h}\;$is the total export value in EUR and $Q_{it}^{h}$ is the total export weight in kilogram. Export quantity, export value, HICP of food index, and cheese production data series are downloaded from Eurostat. Raw milk prices and crude oil prices are downloaded from the European Commission Milk Market Observatory and the World Bank database, respectively. In this study, the software MATLAB 2019a is used for statistical analysis and Microsoft Excel is used for the depiction of figures. Specifically, the GVAR Toolbox 2.0 developed by Smith and Galesi [50] was applied for the GVAR model estimation and analysis.

The average share of each country’s total dairy export values compared to the EU’s total dairy export values from 2010 to 2017 was used for the fixed weights as shown in Table 1.

(18) $$W_{ij}=\frac{1}{8}\sum_{t=2010}^{2017}\frac{ExV_{t}^{ij}}{ExV_{t}^{i\mathrm{EU}}}$$

where *i* represents the export countries, *j* represents the partner countries, $ExV_{t}^{ij}$ represents the export values of the reporter *i* to partner *j* in the year *t*, $ExV_{t}^{i\mathrm{EU}}$ represents the export values of the reporter *i* exporting to other EU member countries in the year *t*.

Therefore, the average of the share of reporters’ dairy export value to partners in the reporters’ dairy export value to the rest of the EU member countries from 2010 to 2017 is calculated as the weight. In the above table, the exporters (reporters) are displayed in the column and each column sums to 1.

The dairy export flows by value are constructed as the weights to indicate the mutual trade partnership among studied countries. As the weights show, the main dairy export destinations of the UK are Ireland, Germany, France, and the rest of the EU, while Irish dairy is primarily exported to the UK. The Netherlands and Italy share similar export destinations. Germany and France share similar export destinations.

## 4. Empirical Results and Analysis

### 4.1. Cointegration, Weak Exogeneity Tests, and Contemporaneous Effects Analysis

Firstly, the unit root test is conducted to test the stationarity of all the variable series. The results of augmented Dickey–Fuller (ADF) unit root tests for each variable series in intra-EU GVECM and extra-EU GVECM are shown in Table 2. As indicated by the ADF tests, the intra-EU export price series of France and cheese production index series for all countries except the Netherlands are stationary, while all other series included in this model do not reject the null hypothesis of non-stationarity and are stationary at the first difference level, thus they are I (1). Therefore, the variables in each country or region could have cointegrating relationships that require cointegration tests.

**Figure A1:**
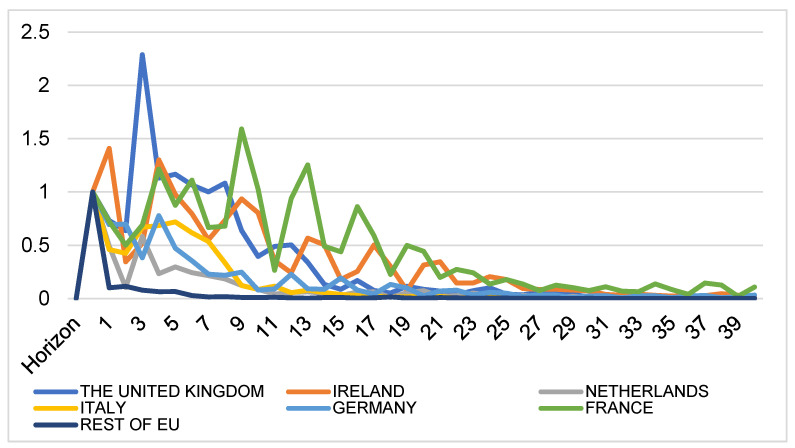
Persistence profile of the effect of system-wide shocks to the cointegrating relations of the intra-EU GVAR model.

**Figure A2:**
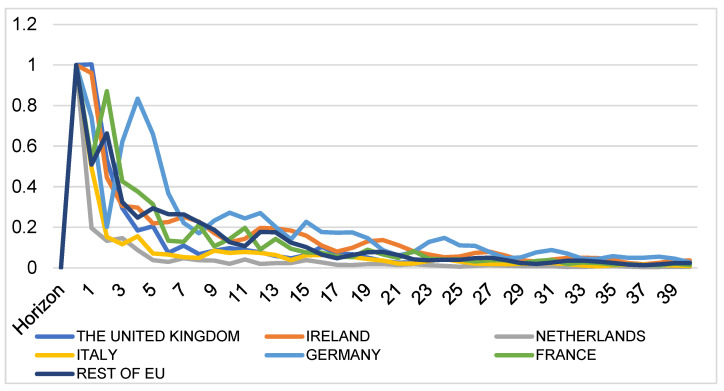
Persistence profile of the effect of system-wide shocks to the cointegrating relations of the intra-EU GVAR model.

For each country or region’s VARX model, the orders of *p* and *q* were selected using the AIC criteria based on the pre-constraint 4 ≥* q(i)* ≥1. To find appropriate lag orders, it is assumed that the model has both an unrestricted intercept and a co-trending restriction to each country or region model. The cointegration test is conducted using the maximum eigenvalue statistic and the trace statistic. The results are shown in Table 3. There are cointegrating relations among the variables for every studied country. Therefore, the vector-error correction models will be implemented in the following analysis. In the intra-EU GVAR model, the selected *p* and *q* lags for most of the relations fall into the constraint of no larger than 4. However, in the extra-EU GVAR model, except the VARX model of the UK and Ireland, the selected lags of all the other country-level models are constrained to 4 lags in terms of both *p* and *q*. As shown in Table 3, the rank for country cases was changed to 1 in all cases due to the fact that using the tested cointegrating relations will cause model instability, following many previous GVAR studied such as Assenmacher [51] and Bettendorf [25]. This ensures model stability and ensures that the analysis better serves the objectives of this study. The model stability tests are also conducted as shown in Appendix A. The results of structural stability tests for Intra-EU GVAR model as shown in Table A1 and for extra-EU GVAR as shown in Table A3, as well as persistence profile figures as depicted in Figure A1 and Figure A2 indicate that both intra-EU and extra-EU GVAR models are structurally stable.

Given the results that there are cointegrating relations among the variables, the VARX Equation (1) can be rewritten in its vector error-correction (VECMX) form:

(19) $$\Delta x_{it}=a_{i0}+a_{i1}t+\Lambda_{i0}\Delta x_{it}^{*}-\Pi_{i0}{Ζ}_{i,t-1}+\overset{p}{\underset{l=1}{\sum }}H_{il}\Delta {Ζ}_{i,\;t-l}+\varepsilon_{it\;}\;$$

where $\Pi_{i0}=-(A_{i0}-\overset{p}{\underset{l=1}{\sum }}A_{il}),$ $H_{il}=-\overset{p-1}{\underset{l=1}{\sum }}A_{i,\;l+1},\;\Delta =1-L$ is the first-order difference; $Z_{it}={\left(x_{it}^{\prime },\;x_{it}^{{*}^{\prime }}\right)}^{\prime }$ be $k_{i}+k^{*}$ dimensional vector. $x_{it}$ and $x_{it}^{*}$ is the variable vector as defined previously in Equations (13)–(16).

The important assumption of the GVAR model estimation is the weak exogeneity of foreign variables. The weak exogeneity hypothesis has a profound implication for the analyses of the international market for EU cheese. It indicates that all the countries in the model jointly determine each other and there is no leader, which is consistent with the assumption of this study that cheese export prices of the EU member states are jointly determined. It also allows the short-run impact of one or more main exporting countries on export price dynamics for the EU as a whole.

The weak exogeneity hypothesis can be tested following the procedure proposed by Johansen [52] and Harbo et al. [53] to perform the regression below for each GVECX country model and each foreign variable in the vector of $x_{it}^{*}$:

(20) $$x_{it,l}^{*}=\mu_{il}+\overset{r_{i}}{\underset{j=i}{\sum }}\gamma_{it,l}{\hat{ECT}}_{i,\;t-1}^{j}+\overset{p_{i}}{\underset{p=1}{\sum }}\Phi_{ip,t}\Delta x_{i,\;t-p}+\overset{q_{i}}{\underset{m=1}{\sum }}\theta_{im,\;t}\Delta y_{t,\;t-m}^{*}+\varepsilon_{it,l}$$

In this Equation (20), $\Delta x_{i,\;t-p}$ is the vector of domestic variables in first differences, where $p=1,\;2,\ldots ,p_{i}$ and $p_{i}$ is the selected lag order of the domestic vector of variables for each country model *i* = 0, 1, 2, 3 in this study. $\Delta y_{t,\;t-m}^{*}$ is the vector of foreign and global variables in first differences, where $m=1,2,\ldots ,q_{i}$ and $q_{i}$ is the lag order of the foreign and global vectors of variables for each *i*th country model. ${\hat{ECT}}_{i,\;t-1}^{j}$ is the estimated error correction term, where $j=1,\;2,\;\ldots ,\;r_{i}$ and $r_{i}$ is the number of cointegrating relations for the VECM of country *i*. The weak exogeneity test is to test the null hypothesis that $y_{i,\;j-1}$ = 0 for each $j=1,\;2,\;\ldots ,\;r_{i}$ using the F test.

Table 4 reports the results of the weak exogeneity tests which indicate that the null hypothesis of weak endogeneity for foreign-specific variables cannot be rejected at the 95% significance level if the value is smaller than the critical value. As the results show, most of the foreign-specific variables are weakly exogenous. However, it may be a concern that, for intra-EU GVAR model: (1) all the foreign variables in the model for France are not weakly exogenous. Besides, (2) export price variable in the models for the Netherlands and Germany, the production variable in the models for Ireland and Germany, the HICPF variable in the models for the Netherlands, and the raw milk price variable for Germany all reject the weak exogeneity hypothesis. For extra-EU GVAR model: production variable for models of the Netherlands, Italy, Germany, France, and the rest of the EU, HICPF variables for model of Germany, and crude oil price variable for model of France reject the weak exogeneity hypothesis. However, this does not seem to be too serious a violation and could be due to insufficient dynamics [54,55]. However, for the following analysis, the France intra-EU model and shocks to production in the extra-EU model should take this into consideration. The possible significant impacts might be due to the violation of weak exogeneity. The GVAR model partially satisfied the condition to conduct comparative studies on the relationship between country-specific and foreign-specific variables.

The effects foreign-specific variables have on the corresponding domestic variables can be analyzed when performing the cointegrating VECMX as reported in Table 5. The contemporaneous effects could be interpreted as the impact elasticities to show the short-run relationship between domestic and foreign variables. Table 5 shows the following results: (1) The effects coefficients for the extra-EU cheese export price index for all countries or region are positive with the exception of France. However, only Ireland and the rest of the EU have significant estimates, which means foreign-specific extra-EU export prices do not have a strong effect on domestic cheese export prices for each country. The effect coefficients of intra-EU cheese export price of the UK, the Netherlands, Germany, and the rest of the EU are estimated to be positive and statistically significant, while the coefficients for other countries’ cheese export prices are estimated to be negative and not statistically significant; (2) In both models, the effects coefficients estimated for CPI for food in all the countries, with the exception of Ireland, are positive and statistically significant, implying that in the short run, domestic food prices in one EU member state are easily affected by changes in other EU member states’ food prices regardless of intra-EU or extra-EU export prices; (3) In both models, the effect coefficients estimated for cheese production for all the countries, except Ireland, are positive and statistically significant, suggesting there are relatively strong co-movements among EU member states’ cheese production in the short term. However, weak exogeneity is not found in the production series, so the strong co-movement could be due to the endogenous relationship between domestic and foreign production; (4) In the extra-EU GVAR model, it is statistically significant for the short-run co-movement of foreign and domestic farm-gate raw milk price indices of the UK, Germany, and the rest of the EU, and all the countries have a positive short-run contemporaneous effect coefficient. In the intra-EU models, it is statistically significant for the short-run co-movement of foreign and domestic farm-gate raw milk price indices of the UK, Ireland, the Netherlands, Germany, and the rest of the EU, and all the estimated coefficients are positive. This indicates that possible linkages exist across the EU member states for farm-gate milk prices.

### 4.2. Dynamic Analysis: General Impulse Response Function Analysis

In this section, the dynamic analysis will be conducted using the general impulse response function (GIRF) analysis proposed by Koop et al. [56] and developed by Pesaran and Shin [57] to understand the dynamic interactions of cheese export prices with other influencing factors and global variables. A GIRF approach can be sufficient and effective to identify the short-run dynamics of the established GVEC models without a priori information and strong structural assumptions, as it is invariant to the ordering of the variables and the ordering of the studied countries. To better investigate the research objective of this study and display the dynamic interlinkages more effectively, only the GIRF of cheese export prices for the studied countries is presented here with a focus on the 40 months after the simulated shock. The following analyses are based on GIRF figures for cheese export prices in the different EU member states, and the bootstrap median estimates of GIRFs are visualized for analysis. Bootstrap median estimates of GIRFs with 90% bootstrap error bounds are visualized in the supplementary materials (Figures S1–S55). Five shocks were simulated to analyze the dynamic characteristics of cheese export prices and the impacts of the shocks on intra-EU and extra-EU export prices in intra-EU and extra-EU GVECMs, respectively:

1. A one-standard-error positive shock to cheese export prices in the intra-EU and extra-EU models, respectively;

2. A one-standard-error positive shock to crude oil price;

3. A one-standard-error positive shock to raw milk price;

4. A one-standard-error positive shock to CPI for food;

5. A one-standard-error negative shock to cheese production.

#### 4.2.1. Spatial Price Transmission and Market Integration for Cheese Exports

One of the essential ideas behind the creation of the EU is for common policies to form an internal market and foster the integration of spatially separated commodity markets, to facilitate the free movement of products among EU members and for trade with non-EU countries as a common economy in international markets. In terms of the dairy market, the CAP, with principles supporting free trade within the EU and common external tariffs and policies externally, was developed to facilitate the market integration of EU agricultural and food markets. Prices can act as appropriate signals for the redeployment of resources to profit from trade in a well-integrated market. The lack of market integration in the EU could cause trade-liberalizing initiatives and EU economic development to not function as predicted [58]. Hence, examinations of market integration are important for the EU.

##### Intra-EU Cheese Export Price Transmission and Market Integration

**Figure 5:**
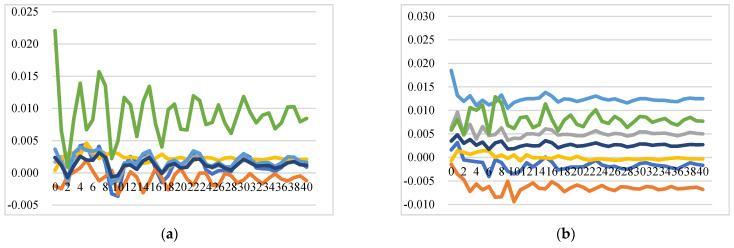

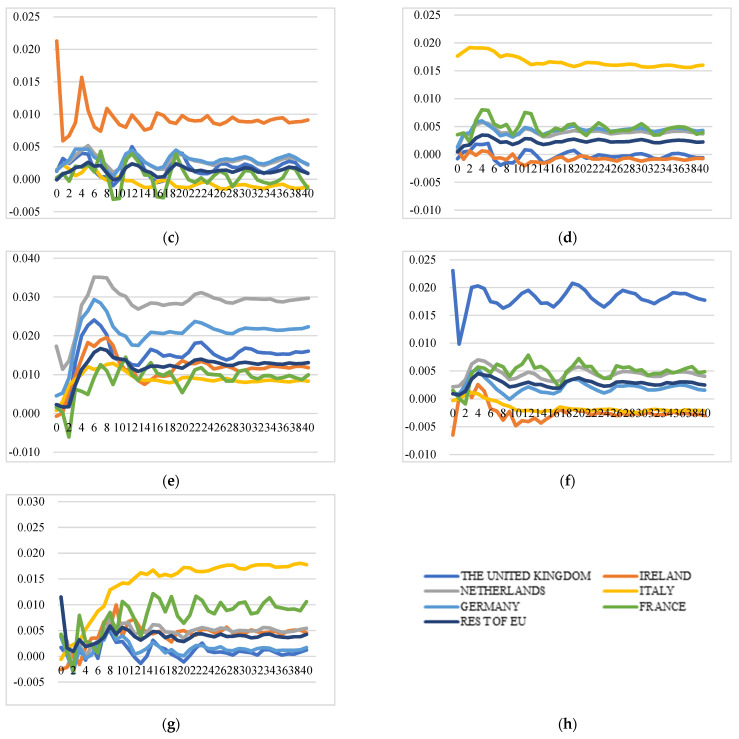
Generalized impulse response functions (GIRFs) of six EU member states’ intra-EU cheese export prices after a positive one-standard-error shock to intra-EU cheese export price (bootstrap median estimates). (**a**) GIRFs after a shock to France’s intra-EU export price. (**b**) GIRFs after a shock to Germany’s intra-EU export price. (**c**) GIRFs after a shock to Ireland’s intra-EU export price. (**d**) GIRFs after a shock to Italy’s intra-EU export price. (**e**) GIRFs after a shock to the Netherlands’ intra-EU export price. (**f**) GIRFs after a shock to the UK’s intra-EU export price. (**g**) GIRFs after a shock to the rest of the EU’s intra-EU export price. (**h**) legend.

One of the prevalent definitions for market integration is the degree to which shocks from demand and supply sides are transmitted from one location (country/region, etc.) to another, with prices showing common movements in different locations in the long run [59,60]. Figure 5 illustrates the GIRFs of intra-EU export prices after a positive shock to intra-EU export price, which demonstrates how a country’s export price responds to its own and other EU countries’ price shocks. In general, the intra-EU cheese export market is not well integrated, and the positive shock to one country’s cheese export price leads to an immediate increase in the export price itself at a level of around 1.8% (for the Netherlands, it itself increases by around 3%), and also increases export prices of other EU member states with similar price dynamic patterns but at lower levels. Notably, there are several insightful findings from the GIRFs of intra-EU export price transmission: (1) Strong trade relationships exhibit higher price response: The more one country imports from exporters, the more the price of this country is affected by its exporters’ price shocks. For instance, the shock to Germany’s intra-EU export price will trigger higher increases in the intra-EU export prices of France, the Netherlands, and the rest of the EU at the level of 0.77%, 0.51%, and 0.27% over time, respectively. (2) Large exporters are significantly affected by other intra-EU countries’ cheese prices: as the top three largest EU cheese exporters for the intra-EU export market, the prices of Germany, the Netherlands, and France, together with the aggregated prices of the rest of the EU countries, are estimated to have a positive and significant response to positive shocks to other countries’ prices. The reasons could be that major exported cheeses of Germany and the Netherlands are classic and homogenous products without name protection and with similar price settings. (3) Ireland and Italy are not well integrated into the EU market: Positive shocks to prices of France, Germany, Italy, and the UK do not significantly affect prices of Ireland and Italy, while the positive shocks to their own prices, and prices of the Netherlands and the rest of the EU have significant and positive impacts on the prices of Ireland and Italy. This might be because Ireland and Italy do not compete directly with other countries due to speciality types of cheese with product differentiation. (4) A positive shock to the price of the rest of the EU will cause an increase in the prices of all the studied countries, with the prices of Italy and France displaying long-lasting and significant increases compared to prices for the other countries.

##### Extra-EU Cheese Export Price Transmission and Market Integration

**Figure 6:**
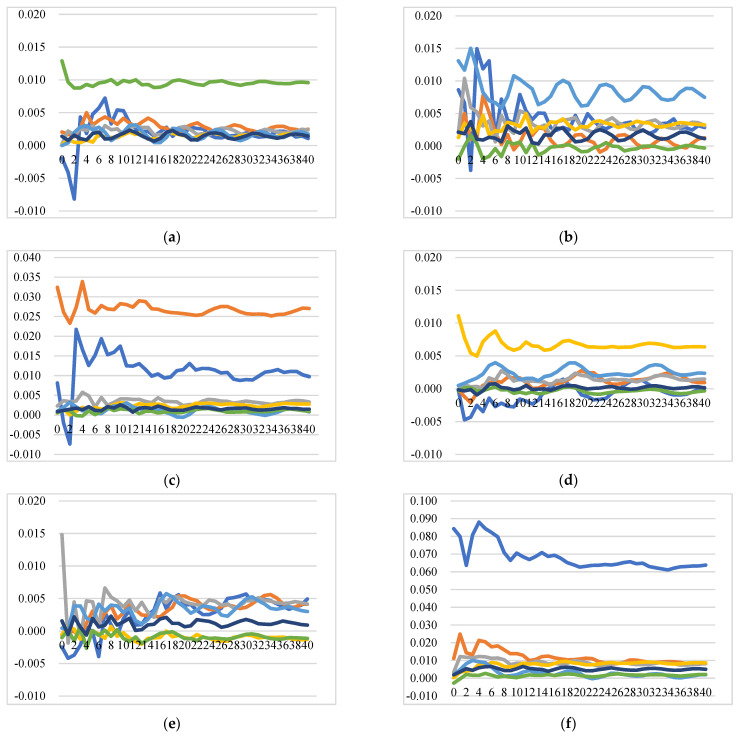

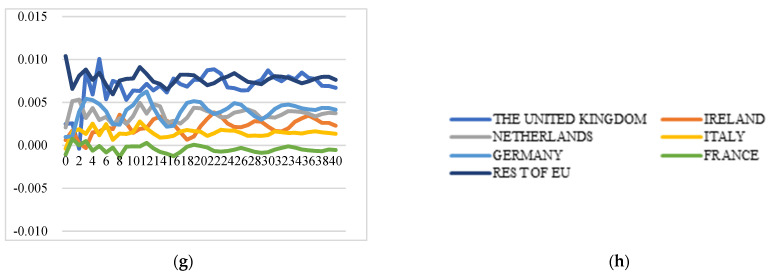
Generalized impulse response functions (GIRFs) of six EU member states’ extra-EU cheese export prices after a positive one-standard-error shock to extra-EU cheese export price (bootstrap median estimates). (**a**) GIRFs after a shock to France’s extra-EU export price. (**b**) GIRFs after a shock to Germany’s extra-EU export price. (**c**) GIRFs after a shock to Ireland’s extra-EU export price. (**d**) GIRFs after a shock to Italy’s extra-EU export price. (**e**) GIRFs after a shock to the Netherlands’ extra-EU export price. (**f**) GIRFs after a shock to the UK’s extra-EU export price. (**g**) GIRFs after a shock to the rest of the EU’s extra-EU export price. (**h**) legend.

Price transmission in intra-EU and in extra-EU export markets follows different dynamics. The extra-EU cheese export price transmission indicates that this export market is not well integrated as well. However, there are clear price interactions between Ireland and the UK. A positive shock to one country’s export price instantly increases the price itself at the level of around 1% (for Ireland and the UK, the levels are 2.7% and 6%, respectively), while it increases other country’s export price at lower levels. Several findings can be identified from the GIRFs results depicted in Figure 6: (1) a positive shock to Germany’s price will cause increases in prices of Germany, the UK, the Netherlands, and Italy, although at a relatively lower level than the increase in itself. However, the positive shocks to the prices of France and Italy do not have a significant impact on other country’s extra-EU export prices. (2) A positive shock to the price of the Netherlands will lead to an increase in the prices of itself, Germany, Ireland, and the UK at a similar level, which means the extra-EU export price of the Netherlands has strong spill-over effects on the prices of Germany, Ireland, and the UK. (3) A positive shock to Ireland’s price leads to significant price inflation in the UK at a level of 1%, while a positive shock to the UK’s price leads to significant and long-lasting price inflation of itself (6%) and Ireland (2%). (4) The extra-EU export price of France is not affected by price shocks to other countries and shows no interlinkages with other prices.

In summary, market integration exists in the extra-EU cheese export market for several countries; however, France and Italy, the second and third largest extra-EU exporters, are not integrated in the extra-EU market because they do not directly compete with other countries due to product differentiation. The price shocks of the UK and Ireland have less price influence on other EU countries. However, there is strong market integration between these two counties: The prices of Ireland and the UK are vulnerable to direct shocks to their own prices, and the price shock will transmit to each other at a larger scale than other countries and the impact will last longer. The shocks to the Netherlands’ prices will cause a swifter and more long-lasting response from their competitors at the similar level and will have strong contagious effects.

#### 4.2.2. Impact of Crude Oil Price Shocks

Crude oil price is assumed to affect agricultural commodity price via two major channels: (1) directly as input costs: production, transportation, and distribution consume energy that is directly linked to the crude oil price; (2) indirectly from the price transmission of biofuel production: The production of biofuel consumes large quantities of corn which is the feed for cows in the dairy sector. Although the theoretical and some empirical evidence point out the impact of crude oil price on the agricultural commodities prices, there is still no consensus in the empirical literature on the crude oil price transmission to individual agricultural markets [61].

##### Impact on Intra-EU Cheese Export Prices

**Figure 7:**
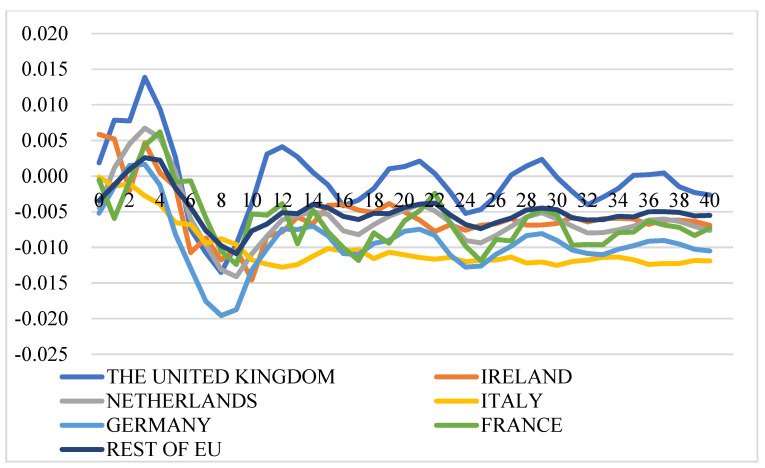
Generalized impulse response functions (GIRFs) of six EU member states’ intra-EU cheese export prices after a positive one-standard-error shock to crude oil price (bootstrap median estimates).

Figure 7 depicts the generalized impulse response functions (GIRFs) of intra-EU export prices after a positive shock to the price of crude oil. Overall, a positive shock will only cause slight increases in the intra-EU export price for the first four months, while the positive impact dies down over time. Crude oil price inflation is estimated to cause higher intra-EU export price changes in the UK and the Netherlands than in other EU countries. Specifically, Ireland and the Netherlands display similar response patterns after the shock, while the impact on the UK’s export price is the greatest. In summary, crude oil price has negligible impact on intra-EU cheese export prices. The reasons might be that the EU dairy sector is characterized by grass-fed and less industrialized production processes which are less reliant on crude oil and energy.

##### Impact on the Extra-EU Cheese Export Price

**Figure 8:**
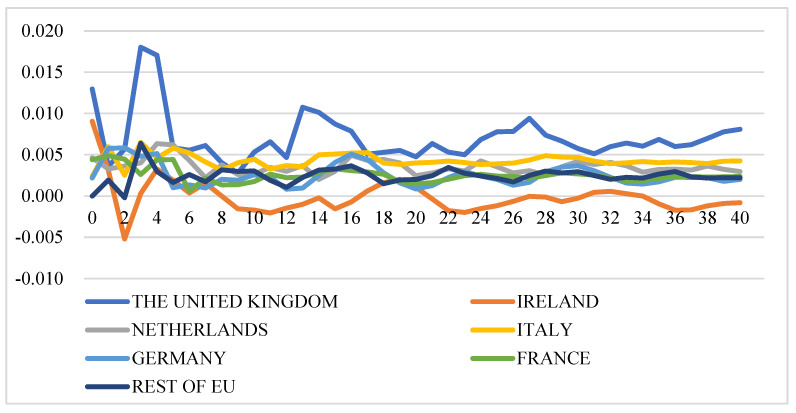
Generalized impulse response functions (GIRFs) of six EU member states’ extra-EU cheese export prices after a positive one-standard-error shock to crude oil price (bootstrap median estimates).

Figure 8 illustrates the generalized impulse response functions (GIRFs) of extra-EU export prices after a positive shock to the crude oil price. Different from the responses of intra-EU export prices, the crude oil price inflation is estimated to cause more significant extra-EU export price rises in all the countries. Moreover, the GIRFs patterns show obvious differentiations among the studied countries. The impact on the UK’s cheese export prices is the highest, while the impact on Ireland is negligible over time. The responses in the extra-EU export prices of Italy, the Netherlands, Germany, and the rest of the EU display similar patterns over time and they show higher increases at a level of 0.5%. So, extra-EU export prices for EU cheese exporters are more vulnerable to crude oil price shocks. This might be due to the fact that the economies of major importers such as the US, Japan, and Saudi Arabia, etc., are highly dependent on crude oil and transportation costs connect cheese export prices to energy prices.

#### 4.2.3. The Impacts of Raw Milk Price

Price transmission across countries and from upstream along the supply chain to export price reflects price relationships between domestic and international markets. Understanding price transmission could shed light on welfare allocation for different stakeholders in the dairy industry. If the domestic markets or local suppliers are isolated from the international market, the price in the latter will not respond quickly to changes in prices in domestic national markets [62,63]. Besides, farm-gate raw milk is the main raw material to produce cheeses, so the rise in raw milk prices will increase the final product prices.

##### Impact on the Intra-EU Cheese Export Price

**Figure 9:**
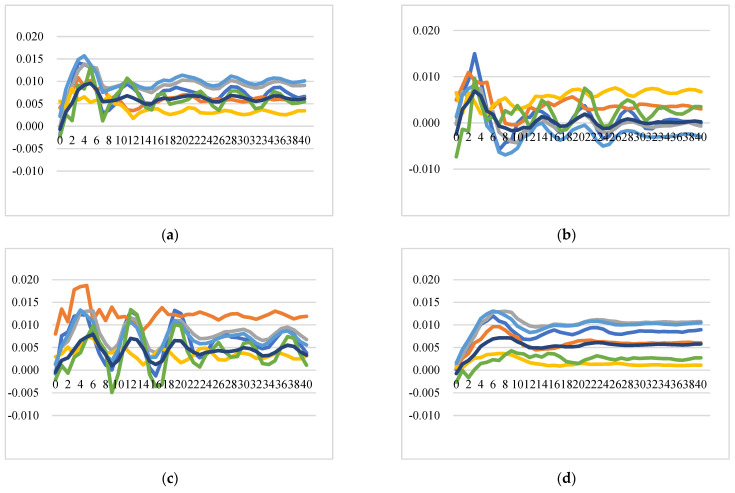

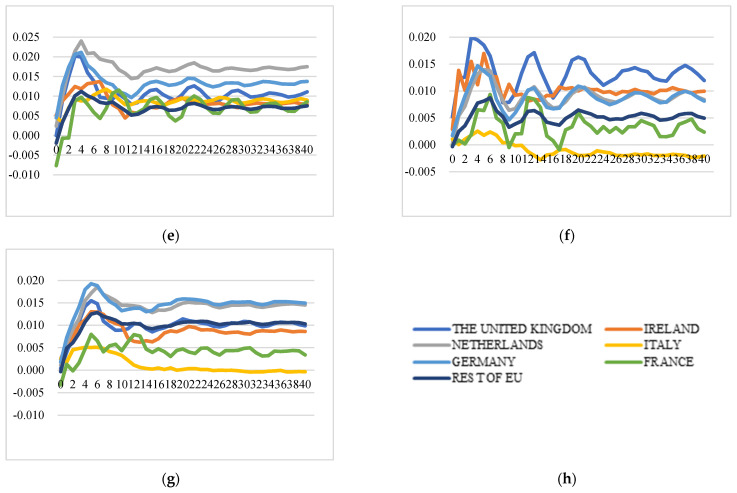
Generalized impulse response functions (GIRFs) of six EU member states’ intra-EU cheese export prices after a positive one-standard-error shock to raw milk price (bootstrap median estimates). (**a**) GIRFs after a shock to France’s raw milk price. (**b**) GIRFs after a shock to Germany’s raw milk price. (**c**) GIRFs after a shock to Ireland’s raw milk price. (**d**) GIRFs after a shock to Italy’s raw milk price. (**e**) GIRFs after a shock to the Netherlands’ raw milk price. (**f**) GIRFs after a shock to the UK’s raw milk price. (**g**) GIRFs after a shock to the rest of the EU’s raw milk price. (**h**) legend.

Figure 9 illustrates the GIRFs of intra-EU export prices after a positive shock to raw milk price. In general, the positive shock to raw milk price in one country is estimated to lead to increases in the intra-EU export prices of all EU countries, though at different levels, which implies strong integration between the domestic and the EU internal markets. There are several insightful results to indicate the pattern and degree of price transmission and market integration in the intra-EU cheese export market: (1) the responses of export prices of Germany and the Netherlands show similar patterns and at similar levels after the shocks to raw milk prices of each studied EU country; (2) the impact on the prices of Italy is the lowest after simulated shocks to raw milk prices of France, Ireland, Italy, the UK, and the aggregated rest of the EU, while the impact on the prices of Italy is highest after a shock to the raw milk price of Germany; (3) a shock to the raw milk price of the Netherlands has the most significant impact on export prices of all the EU countries, while Germany has the least impact on export prices of all the countries over time.

In summary, the intra-EU export price and raw milk price transmission found relatively strong integration of EU internal markets with domestic raw milk markets, dominated by local supply shocks and quick contagion effects and price adjustments. Italian cheese export prices are not significantly affected by raw milk price shocks.

##### Raw Milk Price Shock’s Impact on the Extra-EU Cheese Export Price

**Figure 10:**
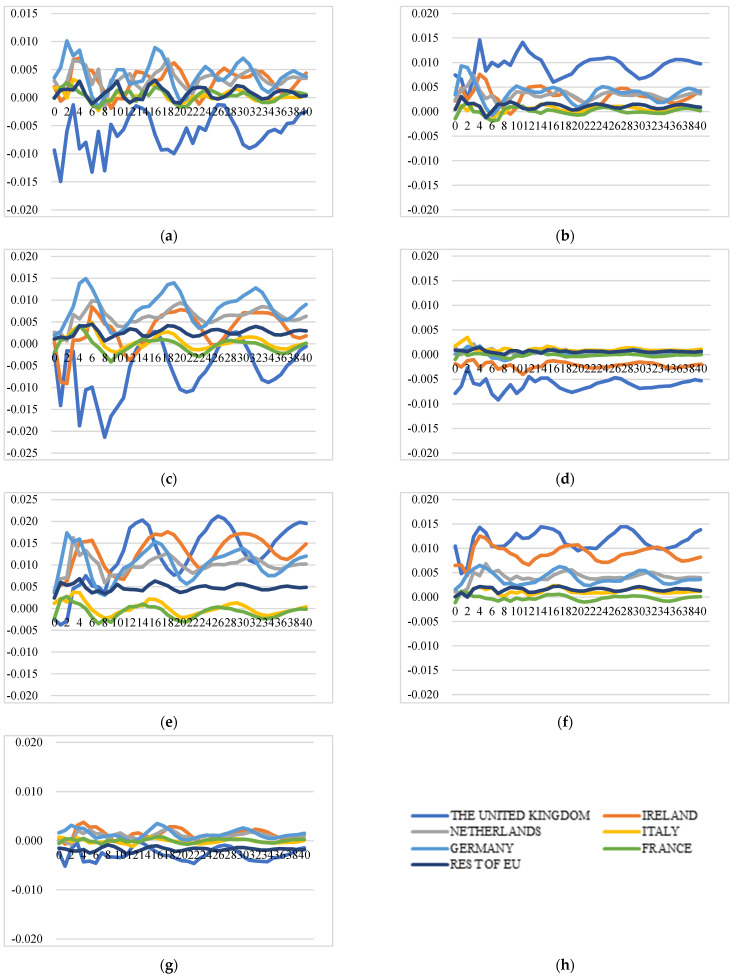
Generalized impulse response functions (GIRFs) of six EU member states’ extra-EU cheese export prices after a positive one-standard-error shock to raw milk price (bootstrap median estimates). (**a**) GIRFs after a shock to France’s raw milk price. (**b**) GIRFs after a shock to Germany’s raw milk price. (**c**) GIRFs after a shock to Ireland’s raw milk price. (**d**) GIRFs after a shock to Italy’s raw milk price. (**e**) GIRFs after a shock to the Netherlands’ raw milk price. (**f**) GIRFs after a shock to the UK’s raw milk price. (**g**) GIRFs after a shock to the rest of the EU’s raw milk price. (**h**) legend.

Figure 10 illustrates the GIRFs of extra-EU export prices after a positive shock to raw milk price. In general, the shocks to raw milk price lead to less significant changes in extra-EU export prices of the studied countries. In the international market outside of the EU, integration between raw milk price and cheese export price is weak. Specifically, (1) the shocks to raw milk prices of Italy and the rest of the EU barely have an impact on the export prices of all the countries. (2) Positive shocks to raw milk prices of France and Ireland even decrease the export price of the UK. (3) In the extra-EU market, the export prices of the Netherlands and Germany display similar patterns and a similar response level after the positive shocks to raw milk prices of every studied country. (4) Export price of Ireland responds positively and swiftly after a shock to raw milk price of the UK. (5) Export prices of France and Italy are barely affected by the shocks to raw milk prices of every studied country, including their own domestic raw milk prices. The reason that export prices of France and Italy are less susceptible to raw milk price changes could be that their most exported cheeses are high-end and differentiated cheeses. For example, Brie (CN04069084) is the most exported and net exported cheese type for France and is known as “The Queen of Cheeses” under the EU geographical protection. Grana Padano, Parmigiano Reggiano (CN 04069061) are the most exported and net exported cheese category for Italy. Grana Padano, granted DOP on 12 June 1996, is one of the few cheeses that can possibly compete with the King of Cheeses; Parmigiano Reggiano.

In summary, there is weak raw milk price and extra-EU export price transmission for EU member states. The export markets of France and Italy are isolated from their domestic raw milk market. Irish extra-EU export prices can be affected by the raw milk price of the UK at similar levels to British extra-EU export prices.

#### 4.2.4. The Impacts of CPI for Food

The consumer price index of food in the EU has been collected and calculated using the Harmonised Index of Consumer Price (HICP) of food (HICP of food is designed for international comparisons of consumer price inflation to ensure the statistical consistency and harmonization of CPI in different EU member states, which allows international comparisons of consumer price inflation for EU member states). HICP of food could indicate domestic food price inflation. The stability of HICP of food serves the purpose of EU monetary policy effectiveness.

##### Impact on the Intra-EU Cheese Export Price

**Figure 11:**
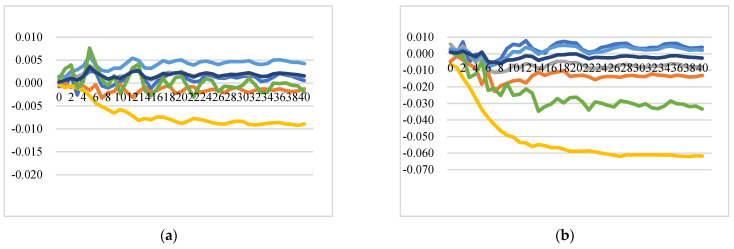

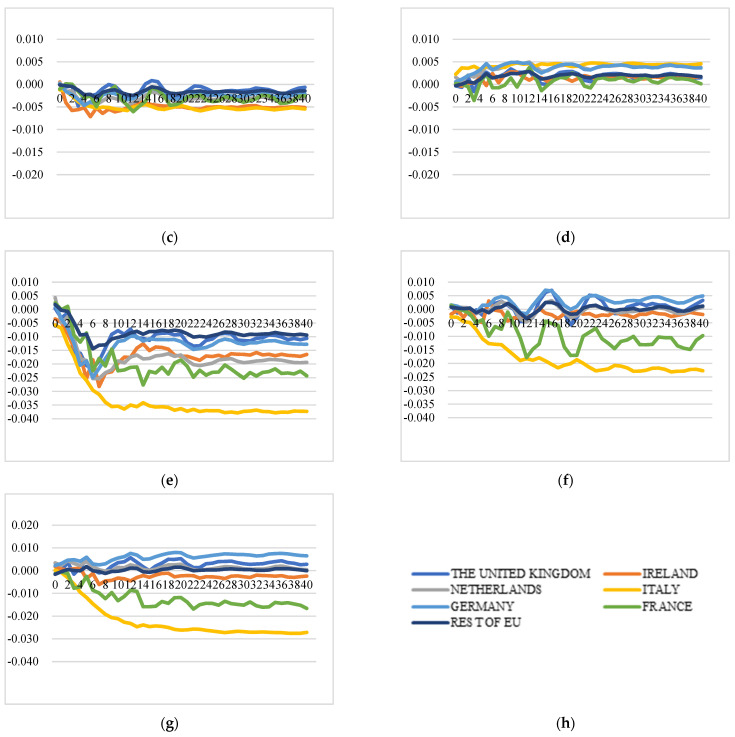
Generalized impulse response functions (GIRFs) of six EU member states’ intra-EU cheese export prices after a positive one-standard-error shock to the Harmonised Index of Consumer Price (HICP) of food (bootstrap median estimates). (**a**) GIRFs after a shock to France’s CPI. (**b**) GIRFs after a shock to Germany’s CPI. (**c**) GIRFs after a shock to Ireland’s CPI. (**d**) GIRFs after a shock to Italy’s CPI. (**e**) GIRFs after a shock to the Netherlands’ CPI. (**f**) GIRFs after a shock to the UK’s CPI. (**g**) GIRFs after a shock to the rest of the EU’s CPI. (**h**) legend.

Figure 11 illustrates the GIRFs of intra-EU export prices after a positive shock to HICP of food. The direction for the responses of export prices after a positive shock to the HICP of food is not consistent across countries: (1) The export prices of most countries increase after a positive shock to Italy’s HICP of food, with prices of Italy, Germany, and the Netherlands having common movements. However, the export prices of most countries decrease after positive shocks to HICP of food in Ireland and the Netherlands. (2) The export prices of France, Ireland, and Italy decrease after a positive shock to HICP of food in almost all the countries. (3) The export price of Germany responds positively and more significantly than others after simulated shocks to HICP of food of France, Germany, Italy, the UK, and the rest of the EU.

To sum up, positive shocks to the food CPI led to significantly negative responses in Italian cheese prices and slight negative responses in French and Irish cheese prices. The reasons behind the results are that the most exported Italian cheese represents a type of high-end product. According to economic theory of consumption, as price level rises, interest rates rise, domestic investment in foreign countries decreases, the real exchange rate appreciates, and net exports decrease, which suggests that inflation means fewer exports and more imports for that country. Consumers are price sensitive to food and for high-end cheese price, elasticity of demand is high. So, demands for Italian high-end cheese decline as consumers give it up to purchase other cheaper and substitute cheeses when inflation occurs in the import countries; then the price of exported Italian cheese will decrease accordingly.

##### Impact on the Extra-EU Cheese Export Price

**Figure 12:**
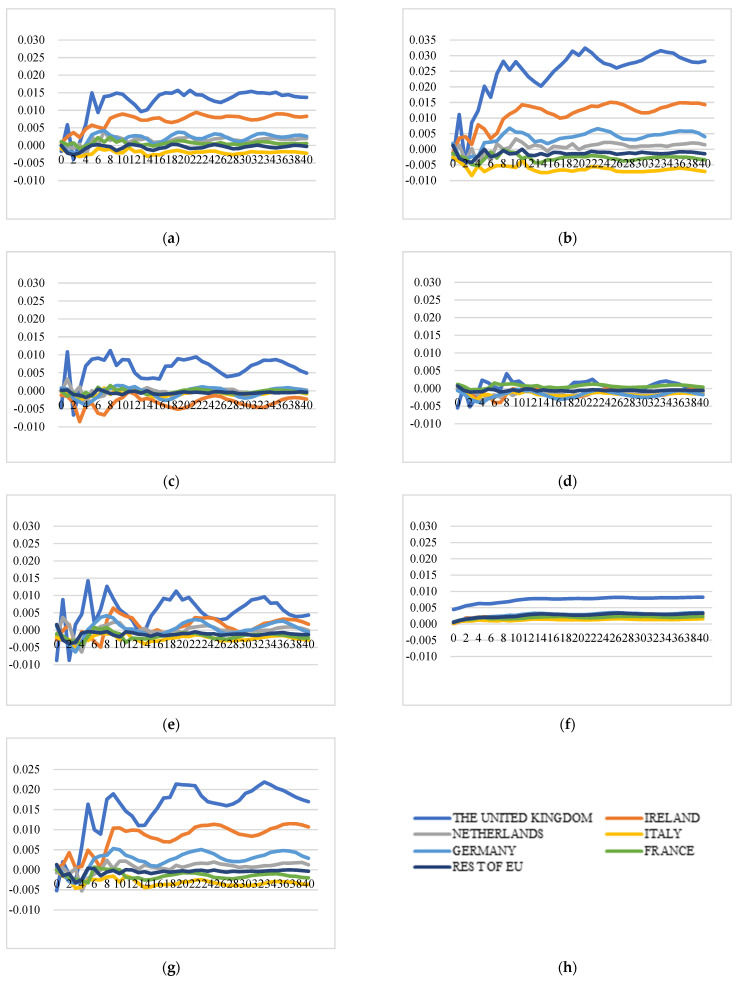
Generalized impulse response functions (GIRFs) of six EU member states’ extra-EU cheese export prices after a positive one-standard-error shock to the Harmonised Index of Consumer Price (HICP) of food (bootstrap median estimates). (**a**) GIRFs after a shock to France’s CPI. (**b**) GIRFs after a shock to Germany’s CPI. (**c**) GIRFs after a shock to Ireland’s CPI. (**d**) GIRFs after a shock to Italy’s CPI. (**e**) GIRFs after a shock to the Netherlands’ CPI. (**f**) GIRFs after a shock to the UK’s CPI. (**g**) GIRFs after a shock to the rest of the EU’s CPI. (**h**) legend.

Figure 12 illustrates the GIRFs of extra-EU export prices after a positive shock to HICP of food. In general, extra-EU export price dynamics are different from intra-EU export price dynamics after a positive shock to HICP of food. The extra-EU export price will increase after a positive shock to HICP of food: (1) the export price of the UK is significantly affected by the HICP of food shocks from all the studied countries, with price increasing at higher levels than others after positive shocks to HICP of food of all the studied countries. (2) The shocks to HICP of food of Ireland, Italy, and the Netherlands are estimated to have insignificant impacts on extra-EU export prices of almost all the countries except the UK. (3) The shocks to HICP of food of France, Germany, and the rest of the EU have significant positive effects on the export price of Ireland at a level of around 1%.

#### 4.2.5. The Impact of Shocks on the Cheese Production Index

Slow growth or decline in production has always been regarded as a critical supply-side factor that can cause sharp increases in global agricultural commodity prices [64,65]. Extreme weather and climate conditions, distorted policies, and wrong market signals might cause cheese production decline in the EU market.

##### Impact on the Intra-EU Cheese Export Price

**Figure 13:**
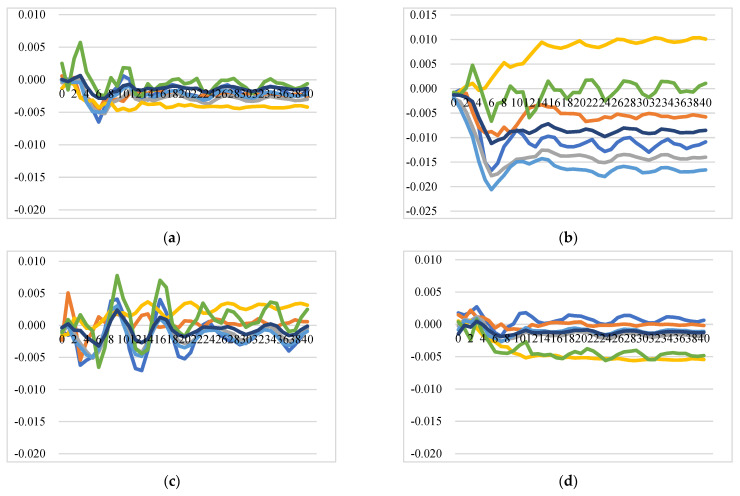

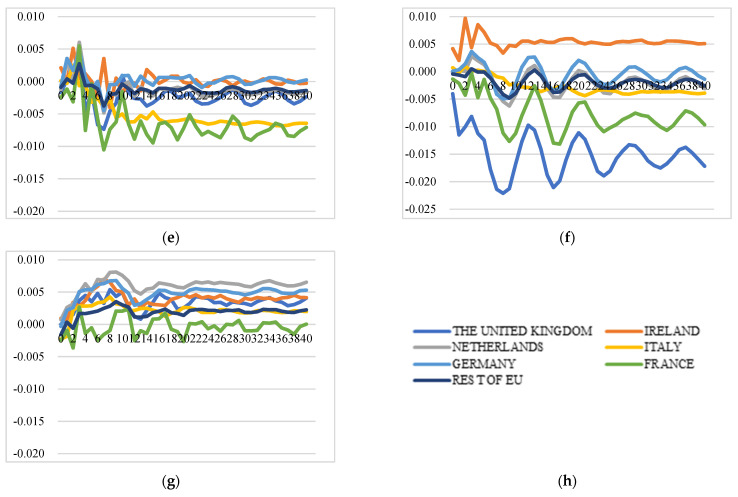
Generalized impulse response functions (GIRFs) of six EU member states’ intra-EU cheese export prices after a negative one-standard-error shock to cheese production (bootstrap median estimates). (**a**) GIRFs after a shock to France’s cheese production. (**b**) GIRFs after a shock to Germany’s cheese production. (**c**) GIRFs after a shock to Ireland’s cheese production. (**d**) GIRFs after a shock to Italy’s cheese production. (**e**) GIRFs after a shock to the Netherlands’ cheese production. (**f**) GIRFs after a shock to the UK’s cheese production. (**g**) GIRFs after a shock to the rest of the EU’s cheese production. (**h**) legend.

Figure 13 illustrates the GIRFs of intra-EU export prices after a negative shock to the cheese production index. In general, the impact direction of negative shocks to cheese production indexes on intra-EU cheese export prices is uncertain: (1) A negative shock to cheese production index of the rest of the EU has a positive impact on export prices of almost all the studied countries. (2) The export price of France remains unaffected after a shock to production indexes of France, Germany, Ireland, and the rest of the EU, while it decreases over time after shocks to production indexes of Italy, the Netherlands, and the UK. (3) The impacts of shocks to production indexes of France, Ireland, Italy, and the Netherlands on the export price of Ireland are negligible, while the export price of Ireland will increase after simulated shocks to production of the UK and the rest of the EU and decrease after a shock to production of Germany. (4) The export prices of many countries decrease after negative shocks to production of Germany, Italy, the Netherlands, and the UK.

##### Impact on the Extra-EU Cheese Export Price

**Figure 14:**
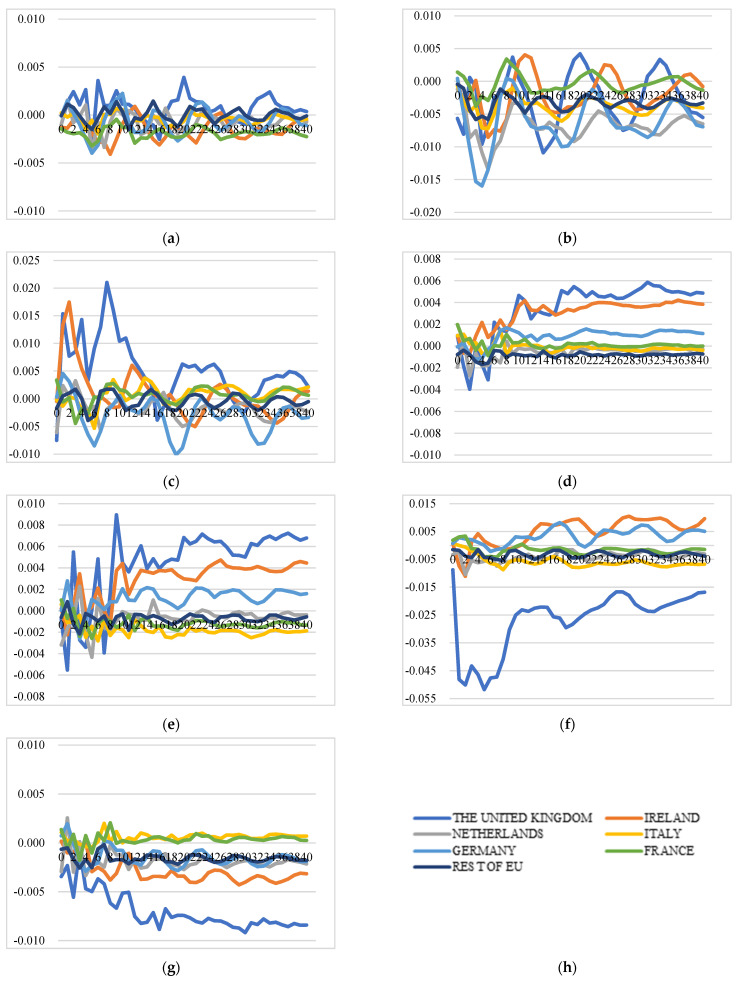
Generalized impulse response functions (GIRFs) of six EU member states’ extra-EU cheese export prices after a negative one-standard-error shock to cheese production (bootstrap median estimates). (**a**) GIRFs after a shock to France’s cheese production. (**b**) GIRFs after a shock to Germany’s cheese production. (**c**) GIRFs after a shock to Ireland’s cheese production. (**d**) GIRFs after a shock to Italy’s cheese production. (**e**) GIRFs after a shock to the Netherlands’ cheese production. (**f**) GIRFs after a shock to the UK’s cheese production. (**g**) GIRFs after a shock to the rest of the EU’s cheese production. (**h**) legend.

Figure 14 illustrates the GIRFs of extra-EU export prices after a negative shock to the cheese production index. The extra-EU export price dynamics are different from intra-EU export price dynamics after one-standard-error shock to the cheese production index and the direction is uncertain as well. In general, production shocks only affect extra-EU export prices of a few countries: (1) the shocks to the cheese production index of France and Germany barely have an impact on extra-EU export prices of all studied countries. (2) GIRFs movement patterns for Ireland and the UK are similar over time with more significant responses than other countries. (3) Negative shocks to Italy and the Netherlands lead to increases in export prices of the UK, Ireland, and Germany, while the impacts are negligible on other countries.

## 5. Conclusions

The results of this study conclude that the shocks have different impacts on intra-EU and extra-EU cheese export prices and the impacts on intra-EU export prices are more consistent and milder than impacts on extra-EU export prices. Several findings are taken from the empirical evidence that provide economic and policy insights.

Firstly, the transmission mechanism of intra-EU cheese export prices implies weak contagion effects in the EU internal market, yet the degree of contagion effects is determined by the trade relationships between member states, which means that the internal market of EU cheese is not well integrated. In the internal market, the importing market’s price dynamics are subject to its major exporters’ price dynamics to a certain degree. The intra-EU export prices of the UK and Ireland are weakly linked, despite a close trade relationship and export dependence of Irish cheese on the UK. However, stronger market integration exists in the external market for several countries, except France, the second largest extra-EU exporter that shows insensitivity to all countries’ price shocks.

Secondly, the intra-EU cheese export prices are not sensitive to global crude oil price shocks, while oil price shocks will have slight effects on extra-EU cheese export prices. This implies that the internal market creates relatively strong protection from external shocks for EU member states.

Thirdly, the different transmission mechanisms between raw milk price shocks and intra-EU prices indicate that the producer price in upstream supply chains has spill-over effects in the internal market of the EU; however, its spill-over effect is weak in the external market. This implies that raw milk prices could be an effective signal for internal trade among EU member states and could primarily affect the intra-EU cheese trade.

Fourthly, food inflation effects on the intra-EU cheese export price are insignificant, and intra-EU export prices of almost all EU member states remain unaffected by food inflation. However, food inflation originating from any of the EU member states could lead to significant extra-EU cheese export price inflation.

Finally, the prices of Ireland and the UK are vulnerable to direct shocks to their own prices, and their extra-EU export prices are highly linked with each other. The movement patterns of extra-EU cheese export prices of Ireland and the UK show similar patterns in the long run. Moreover, the intra-EU cheese export price of Ireland responds significantly to shocks to the UK’s raw milk price and cheese production. The extra-EU cheese export prices of the UK are sensitive to the other EU members’ food CPI. However, the linkages between their intra-EU cheese export prices are relatively weak. So, in the post-Brexit era, Ireland could be more exposed to the effects of the end of transition than the UK, especially if there is no deal and tariffs are imposed.

However, there are several limitations and issues that should be addressed. Firstly, GVAR model has not been employed extensively in the agricultural price transmission area, thus its reliability of results might need to be further justified, especially with comparison with other dynamic panel models and models that could incorporate economic theory. Secondly, foreign variables of France’s VARX in this paper did not pass the weak exogeneity tests; this might affect the reliability of the results. Thirdly, similar to other time-series models, GVAR model relies heavily on time series, thus making it less flexible for incorporating useful policy variables and limiting it by data availability. So, the policy implications of this paper might require further study. Fourthly, as many GIRFs estimated in this paper were not statistically significant, it might be necessary to employ other models or set benchmark models to crosscheck the robustness of the results. Finally, as several cointegrating relations were found that make models structurally unstable, incorporating structural break into the analysis to ensure stability might be another good way to ensure model stability.

## 6. Discussion

The findings are useful for policymakers and dairy sector stakeholders in the EU. Successive CAP reforms (e.g., decrease in intervention prices and milk quota removal, etc.) have created more market-oriented openness to global markets, which brings both challenges and opportunities for the EU dairy sector. Cheese, as a heterogeneous dairy product, exhibits comparative price stability to market shocks. However, free trade and movement of products enhance the price linkages along the supply chain, incorporating intra-EU export prices into the cheese supply chain in the EU internal market, which enhances the interaction of raw material prices and end products, and amplifies the contagious effects of upstream raw milk price volatility and fluctuation transmission. In the EU’s international external market, cheese prices are more susceptible to external shocks, such as crude oil price, than shocks from the internal market. As the EU opens its market further via the bilateral free trade agreement to reduce the duty or even grant duty-free market access, this might increase competition for EU producers.

The findings also provide an interesting debate for the possible impacts of Brexit on the EU dairy industry, especially for Ireland, its neighbor and biggest dairy exporter. Ireland and France are the main exporters of cheeses to the British market, especially fresh cheese and some processed cheeses. The UK’s exit from the EU and the corresponding uncertainties on Brexit have been hotly discussed by political leaders, scholars, and stakeholders of various industries. However, this study implies that the negative impacts on Irish cheese export to the UK might be overestimated and exaggerated because the intra-EU cheese export market of the UK and Ireland is weakly linked in terms of market shocks and price dynamics. However, in the post-Brexit era, the UK should create continuous policies that will not cause additional tariff and non-tariff barriers for dairy sector, considering the UK’s intra-EU export prices are highly linked with the other EU members’ raw milk prices and its close trade relationship with the EU.

There are several research areas that could be explored in future research. First, cheese is a type of dairy product with plenty of varieties. In particular, cheese in the EU is protected by the PDO and PGI that will create the differentiation among cheese products and avoid price arbitrage. The study on products with different varieties might require further study at a more disaggregated product level (6-digit product level, HS 040690, the most traded cheese category; or even further research on 8-digit product level) to discover the competitive advantages for cheese exports in different EU countries. Second, climate change, especially unforeseen extreme weather events, such as increased chances of drought, storms, and disease threats, could constrain milk yields by affecting grass growth and feed supply, thereby influencing farmers’ decisions on cow inventory adjustment, thus further increasing the price volatility of milk and dairy products [66]. Therefore, weather indicators such as rainfall indices (precipitation), temperature indices, and frequency of extreme weather could be incorporated into the model to decipher the impact of climate change on dairy product price transmission. Third, a structural model as proposed by Dees, Mauro, Pesaran, and Smith [54] might be used to incorporate economic theory, institutional knowledge, and other constraints such as policy variables into the analysis to assess the causal relationships and effects of possible shocks on export prices. Fourthly, new conditions are confronted due to Brexit; further studies with regime-changing scenarios should be conducted to find out how tariff and trade policy of the UK after Brexit affects the EU’s dairy exports.

## Figures and Tables

**Table 1:** Trade weights ^1^ based on total dairy product export values.

Countries	The United Kingdom	Ireland	The Netherlands	Italy	Germany	France	Rest of EU
The United Kingdom	0	0.711359	0.044705	0.009894	0.016276	0.047488	0.047138
Ireland	0.255302	0	0.046463	0.004949	0.028415	0.035054	0.02155
The Netherlands	0.089263	0.059234	0	0.062786	0.339163	0.211221	0.236062
Italy	0.07023	0.016594	0.03607	0	0.070562	0.14972	0.071726
Germany	0.129338	0.080058	0.435481	0.405671	0	0.188045	0.381733
France	0.202296	0.063443	0.09476	0.17051	0.143953	0	0.241792
Rest of EU	0.253571	0.069311	0.342521	0.346191	0.401631	0.368472	0

^1^ Note: Trade weights are computed using the Equation (18).

**Table 2:** Augmented Dickey–Fuller (ADF) unit root test statistics for domestic and foreign variables. ^2^

Variables	The United Kingdom	Ireland	The Netherlands	Italy	Germany	France	Rest of EU
$p_{it}^{in}$ (with trend)	−2.88	−1.82	−1.97	−3.17	−1.97	−5.36	−2.55
$p_{it}^{in}$ (no trend)	−2.96	−2.17	−1.87	−2.69	−1.85	−4.03	−1.97
$D.p_{it}^{in}$	−6.51	−8.96	−5.91	−5.81	−5.48	−8.10	−6.70
$p_{it}^{ex}$ (with trend)	−2.53	−1.49	−2.58	−3.29	−2.55	−3.14	−2.37
$p_{it}^{ex}$ (no trend)	−2.66	−1.44	−2.35	−3.46	−2.18	−3.16	−2.45
$D.p_{it}^{ex}$	−10.76	−4.29	−7.41	−7.88	−4.98	−5.60	−7.20
${Pro}_{it}$ (with trend)	−6.74	−5.87	−2.43	−4.04	−6.71	−4.15	−5.61
${Pro}_{it}$ (no trend)	−4.23	−5.94	−1.04	−4.03	−4.49	−2.84	−1.30
$D.{Pro}_{it}$	−5.78	−6.92	−11.06	−9.90	−4.91	−12.13	−5.31
${\mathrm{HICPF}}_{it}$ (with trend)	−1.08	−0.56	−2.38	−2.08	−2.69	−2.22	−1.42
${\mathrm{HICPF}}_{it}$ (no trend)	−2.40	1.14	−1.34	−1.54	−0.80	−1.75	−1.91
$D.{\mathrm{HICPF}}_{it}$	−5.45	−5.92	−6.21	−7.12	−5.18	−6.86	−5.75
$p_{it}^{f}$(with trend)	−2.13	−2.85	−2.51	−2.26	−3.12	−2.26	−3.34
$p_{it}^{f}$ (no trend)	−2.25	−2.71	−2.41	−1.93	−2.90	−2.15	−3.26
$D.p_{it}^{f}$	−4.41	−4.76	−4.19	−4.68	−4.59	−7.09	−4.16
$p_{it}^{in*}$ (with trend)	−2.15	−2.72	−2.20	−2.19	−2.22	−2.26	−2.18
$p_{it}^{in*}$ (no trend)	−2.18	−2.78	−2.06	−2.09	−2.11	−2.03	−2.17
$D.p_{it}^{in*}$	−5.61	−6.30	−5.61	−6.50	−6.29	−5.02	−6.27
$p_{it}^{ex*}$ (with trend)	−1.89	−2.49	−3.26	−2.87	−2.44	−2.78	−2.97
$p_{it}^{ex*}$ (no trend)	−2.32	−2.64	−3.19	−2.83	−2.55	−2.82	−2.81
$D.p_{it}^{ex*}$	−6.59	−10.33	−3.92	−4.70	−5.77	−5.10	−4.36
(with trend)	−5.98	−6.53	−7.12	−6.26	−5.86	−7.19	−6.41
${Pro}_{it}^{*}$ (no trend)	−5.82	−4.37	−3.66	−2.91	−2.01	−2.79	−4.81
$D.{Pro}_{it}^{*}$	−5.44	−5.92	−6.12	−5.39	−6.09	−5.73	−5.95
${\mathrm{HICPF}}_{it}^{*}$ (with trend)	−1.65	−1.09	−2.21	−2.31	−1.67	−1.90	−2.07
${\mathrm{HICPF}}_{it}^{*}$ (no trend)	−1.94	−2.55	−1.44	−1.47	−1.69	−1.60	−1.37
$D.{\mathrm{HICPF}}_{it}^{*}$	−5.27	−5.21	−5.35	−4.97	−4.98	−4.86	−4.94
$p_{it}^{f*}$(with trend)	−3.11	−2.61	−3.22	−3.25	−2.44	−3.24	−3.37
$p_{it}^{f*}$(no trend)	−2.96	−2.64	−3.07	−3.10	−2.33	−3.34	−2.40
$D.p_{it}^{f*}$	−4.31	−3.78	−4.64	−4.50	−3.48	−3.59	−4.04
$p_{it}^{\mathrm{co}*}$ (with trend)	−1.98	-	-	-	-	-	-
$p_{it}^{\mathrm{co}*}$ (no trend)	−0.79	-	-	-	-	-	-
$D.p_{it}^{\mathrm{co}*}$	−5.72	-	-	-	-	-	-

^2^ In this table, the 95% critical values of variables with trend, without trend, and at first difference level are −3.45, −2.89, and −2.89, respectively. In this Table, *D*. denotes the first difference; the following labels are the same.

**Table 3:** VARX order and number of cointegrating relationships for intra-EU GVAR model and extra-EU GVAR model.

	Intra-EU				Extra-EU			
	*p*	*q*	Tested Cointegrating Relations	Adjusted Cointegrating Relations	*p*	*q*	Tested Cointegrating Relations	Adjusted Cointegrating Relations
The United Kingdom	1	4	1	1	1	4	3	1
Ireland	4	4	2	1	2	3	3	1
The Netherlands	2	3	4	1	4	4	2	1
Italy	1	4	3	1	4	4	2	1
Germany	2	2	4	1	4	4	2	1
France	4	4	3	1	4	4	2	1
Rest of EU	2	1	1	1	4	4	2	1

**Table 4:** F statistics of weak exogenous test at the 5% significance level of intra-EU and extra-EU GVECMs.

	Intra-EU					Extra-EU				
Country	$p_{it}^{in*}$	$Pro_{it}^{*}$	${\mathrm{HICPF}}_{it}^{*}$	$p_{it}^{f*}$	$p_{it}^{co}$	$p_{it}^{ex*}$	$Pro_{it}^{*}$	${\mathrm{HICPF}}_{it}^{*}$	$p_{it}^{f*}$	$p_{it}^{co}$
The UK	0.49	0.01	2.87	0.90		0.53	0.12	0.54	1.32	
Ireland	1.36	4.52	0.18	0.74	0.34	0.82	2.66	0.31	0.76	0.07
The Netherlands	4.63	0.20	6.76	0.00	0.42	0.60	14.33	0.23	1.54	0.01
Italy	0.02	0.01	2.25	0.59	1.07	0.92	7.14	1.53	0.80	0.00
Germany	4.96	8.56	0.18	4.05	0.03	3.47	11.03	0.01	1.03	0.37
France	7.96	10.12	4.20	4.94	4.11	3.24	9.26	4.86	3.12	4.32
REU	0.00	0.00	0.19	1.15	0.32	0.74	15.44	1.63	0.27	0.11

Note: critical value for intra-EU UK is 4.04 and for extra-EU UK it is 4.10, and critical values for others are all 3.98.

**Table 5:** Contemporaneous effects of foreign variables on domestic counterparts of intra-EU and extra-EU GVECMs.

		Intra-EU				Extra-EU			
Country		$p_{it}^{in}$	$Pro_{it}$	${\mathrm{HICPF}}_{it}$	$p_{it}^{f}$	$p_{it}^{ex}$	$Pro_{it}$	${\mathrm{HICPF}}_{it}$	$p_{it}^{f}$
The UK	Coefficient	0.62	0.19	0.70	0.75	0.52	0.11	0.64	0.58
	t-ratio	2.36	2.64	2.80	4.40	0.62	1.67	2.69	3.05
Ireland	Coefficient	−0.36	−0.55	0.10	0.81	0.17	0.64	0.04	0.39
	t-ratio	−1.51	−0.71	1.11	3.01	2.27	0.88	0.44	1.49
The Netherlands	Coefficient	0.83	0.31	0.61	0.52	0.29	0.22	0.70	0.25
	t-ratio	4.30	3.42	3.70	3.07	1.12	2.04	3.65	1.18
Italy	Coefficient	−0.13	0.32	0.63	0.25	0.07	0.41	0.76	0.17
	t-ratio	−0.71	2.49	6.14	1.65	0.31	2.87	5.85	1.25
Germany	Coefficient	0.75	0.89	1.14	1.05	0.18	0.83	1.15	1.41
	t-ratio	5.05	14.08	7.16	7.64	0.67	10.24	7.04	7.87
France	Coefficient	0.61	0.70	0.86	0.37	−0.13	0.57	0.80	0.37
	t-ratio	1.45	8.11	6.86	1.54	−0.59	5.93	6.08	1.27
Rest of EU	Coefficient	0.44	0.78	0.83	0.72	0.39	1.02	0.67	0.76
	t-ratio	6.13	13.98	16.02	23.64	2.15	14.55	8.69	17.55

## Data Availability

No new data were created or analyzed in this study. Data sharing is not applicable to this article.

## Supplementary Materials

The following supporting information can be downloaded at: https://www.mdpi.com/article/10.3390/foods11050692/s1.

## Appendix A

Structural tests were conducted to check for potential instability of model parameters over time. Table A1, Table A2, Table A3 and Table A4 outline the statistics and critical values of the structural stability test, namely, maximal OLS cumulative sum (CUSUM) statistic, denoted by PKsup and its mean square variant PKmsq, proposed by Ploberger and Krämer (1992).

The statistic for each variable in intra-EU GVECM is smaller than its corresponding critical value, so the null hypothesis cannot be rejected. So, all the series are structurally stable in our analysis.

**Table A1:** Structural stability tests for intra-EU GVECM: statistics.

Variables	$p_{it}^{in*}$	$Pro_{it}^{*}$	$HICPF_{it}^{*}$	$p_{it}^{f*}$	$p_{it}^{co}$
PKsup					
UK	0.463694	0.8314	0.74255	0.941864	0.605639
Ireland	0.340478	0.645837	0.579837	0.611149	
The Netherlands	0.432776	0.489759	0.468543	0.467494	
Italy	0.784771	0.692224	0.573054	0.27915	
Germany	0.707873	0.559412	0.85324	0.555987	
France	0.676779	0.360939	0.530133	0.603389	
Rest of EU	0.792665	0.714418	0.98315	1.024304	
PKmsq					
UK	0.025343	0.191054	0.156352	0.283167	0.119887
Ireland	0.026055	0.096732	0.039636	0.079477	
The Netherlands	0.038238	0.04151	0.047248	0.035731	
Italy	0.201022	0.128603	0.112031	0.024663	
Germany	0.133299	0.067007	0.223563	0.089999	
France	0.080725	0.031602	0.054723	0.088671	
Rest of EU	0.098799	0.129659	0.36196	0.231079	

**Table A2:** Structural stability tests for intra-EU GVECM: critical values at 90% confidence intervals.

Critical Values	$p_{it}^{in*}$	$Pro_{it}^{*}$	$HICPF_{it}^{*}$	$p_{it}^{f*}$	$p_{it}^{co}$
PKsup					
UK	0.897416	0.905347	0.98611	1.017793	0.923818
Ireland	0.874901	0.790495	0.806877	0.837865	
The Netherlands	0.901752	0.919272	1.01729	0.761882	
Italy	1.050474	0.886111	0.774586	0.993005	
Germany	0.935659	0.947594	0.965402	0.667643	
France	0.906599	0.59459	0.804358	0.859856	
Rest of EU	0.894507	1.098827	1.021138	1.001084	
PKmsq					
UK	0.20909	0.182905	0.258218	0.29313	0.228361
Ireland	0.188175	0.16106	0.160689	0.171427	
The Netherlands	0.204018	0.188362	0.27233	0.120657	
Italy	0.266705	0.191774	0.146847	0.261703	
Germany	0.250349	0.265227	0.236297	0.089392	
France	0.246314	0.076773	0.183384	0.214443	
Rest of EU	0.159505	0.307603	0.274201	0.262245	

Figure A1 depicts the persistence profile of the effect of system-wide shocks to the cointegrating relationship of the GVAR model. It can be observed that the speed of convergence was fast for the Rest of EU, the Netherlands, Germany, and Italy. However, the speed of adjustment was very slow for France and Ireland.

As reported in Table A3 and Table A4, the statistic for each variable in extra-EU GVECM is smaller than its corresponding critical value, so the null hypothesis cannot be rejected. So, all the series are structurally stable in our analysis.

**Table A3:** Structural stability tests for extra-EU GVECM: Statistics.

**Variables**	$p_{it}^{ex*}$	$Pro_{it}^{*}$	$HICPF_{it}^{*}$	$p_{it}^{f*}$	$p_{it}^{co}$
PKsup					
UK	0.903102	0.95222	0.859617	0.887978	0.487304
Ireland	0.556062	0.55685	0.997763	0.452633	
Netherlands	0.520766	0.78265	0.843414	0.626127	
Italy	0.505215	0.700167	0.406689	0.713595	
Germany	0.619687	0.689697	0.57601	0.58145	
France	0.565724	0.59363	0.621403	0.543569	
Rest of EU	0.666355	0.40087	0.983137	0.512081	
PKmsq					
UK	0.156232	0.223775	0.228553	0.195099	0.046937
Ireland	0.038956	0.037861	0.228477	0.041554	
Netherlands	0.040555	0.093031	0.130051	0.065953	
Italy	0.079996	0.041545	0.032675	0.131871	
Germany	0.119449	0.071015	0.041657	0.056514	
France	0.100109	0.044648	0.082583	0.033416	
Rest of EU	0.134005	0.019042	0.298026	0.038923	

**Table A4:** Structural stability tests for extra-EU GVECM: Critical values.

**Critical Values**	$p_{it}^{ex*}$	$Pro_{it}^{*}$	$HICPF_{it}^{*}$	$p_{it}^{f*}$	$p_{it}^{co}$
PKsup					
UK	1.002966	0.951037	1.009028	0.953285	1.00214
Ireland	0.978034	0.733827	1.029252	0.904821	
Netherlands	1.084848	0.890726	1.031816	0.966088	
Italy	1.05471	0.934134	0.959599	1.116367	
Germany	0.942283	0.988631	0.93383	0.745155	
France	0.944595	0.593392	0.860866	1.057197	
Rest of EU	0.80429	1.004234	0.952274	0.743777	
PKmsq					
UK	0.261357	0.251381	0.244638	0.229542	0.271005
Ireland	0.248853	0.118944	0.283955	0.224651	
Netherlands	0.305206	0.209448	0.308843	0.227954	
Italy	0.269976	0.199697	0.245641	0.340598	
Germany	0.256489	0.27992	0.242966	0.139537	
France	0.213078	0.072333	0.217016	0.274276	
Rest of EU	0.183509	0.237285	0.230599	0.154522	

Figure A2 below indicates that the speed of convergence is fast for all countries, so the model is stable over time.
