# Supplementary material for: Market Integration and Price Dynamics under Market Shocks in European Union Internal and External Cheese Export Markets
Source: Foods. 2022 Feb 26;11(5):692. doi: 10.3390/foods11050692 (PMC8909711; doi:10.3390/foods11050692)
Supplement: Supplementary file 1 [file foods-11-00692-s001.zip › foods-1580510-supplementary.pdf]

Supplementary Materials

Huidan Xue, Chenguang Li, and Liming Wang

The following figures depict the GIRFs of intra-EU cheese export prices after simulated shocks (bootstrap median estimates with 90% bootstrap error bounds).

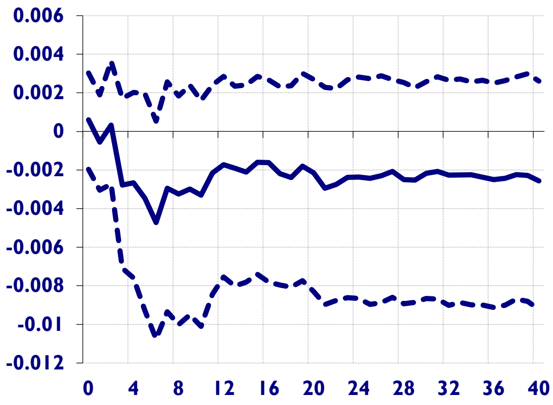

(a) Ireland

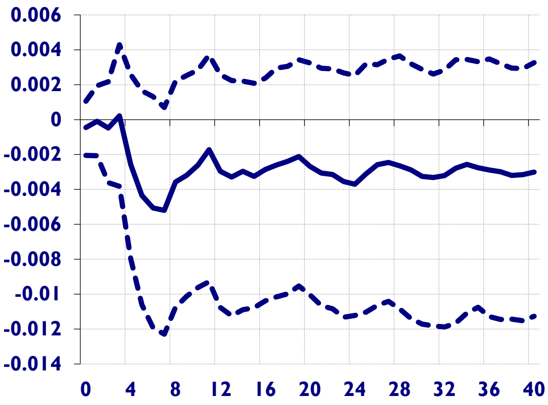

(b) The Netherlands

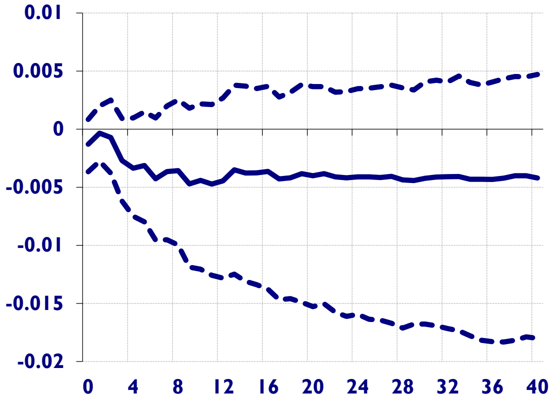

(c) Italy

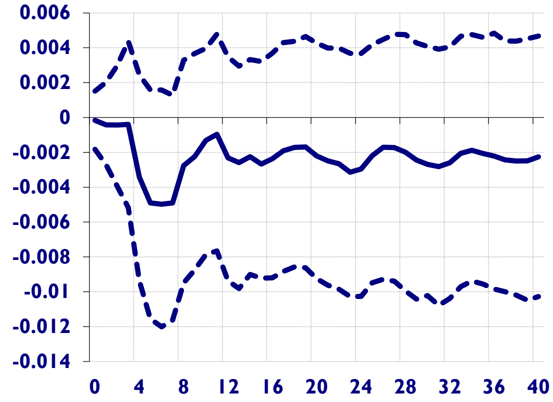

(d) Germany

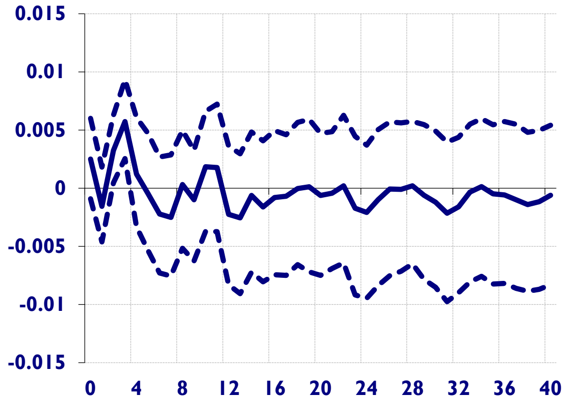

(e) France

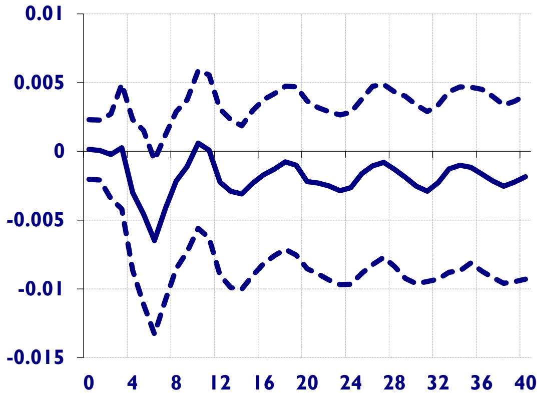

(f) The United Kingdom

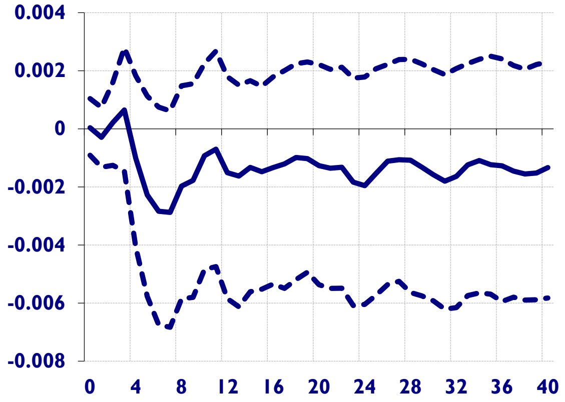

(g) The rest of the EU

Figure S1. Generalized impulse response functions (GIRFs) of intra-EU cheese export prices after a negative one-standard-error shock to France's cheese production

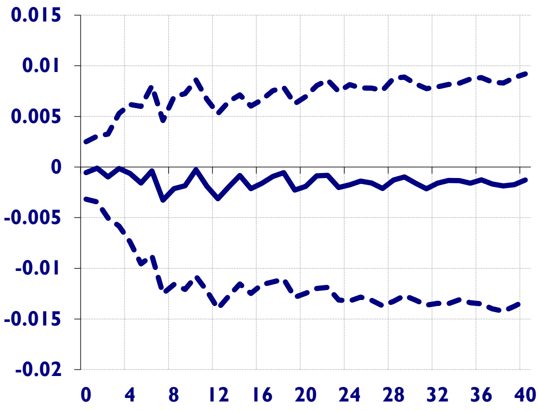

(a) Ireland

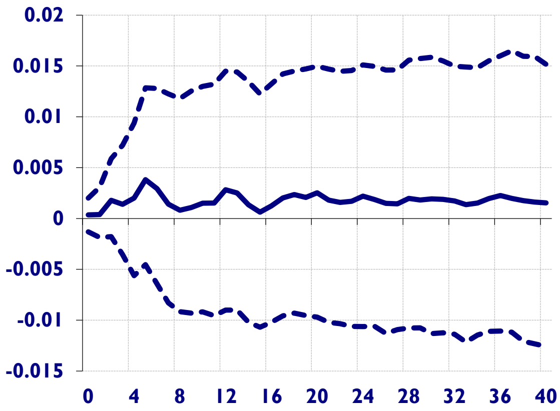

(b) The Netherlands

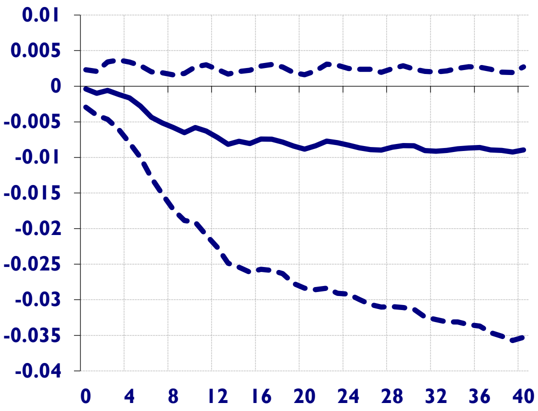

(c) Italy

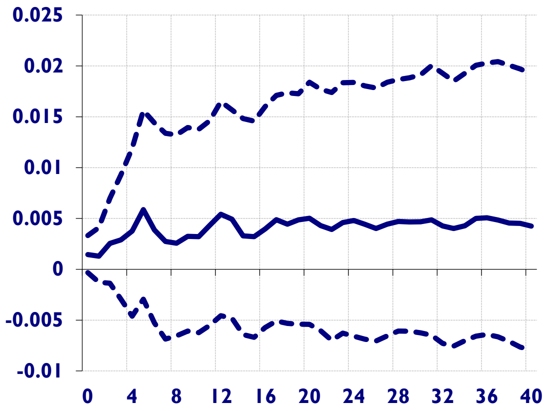

(d) Germany

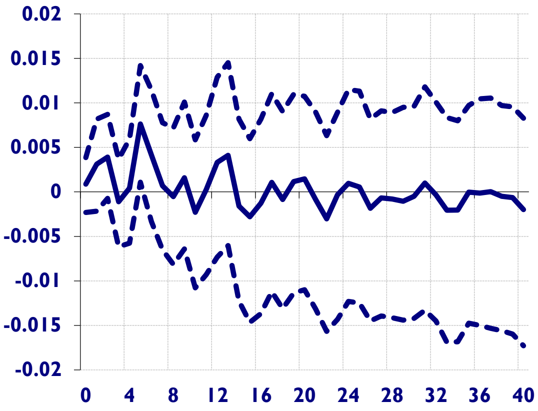

(e) France

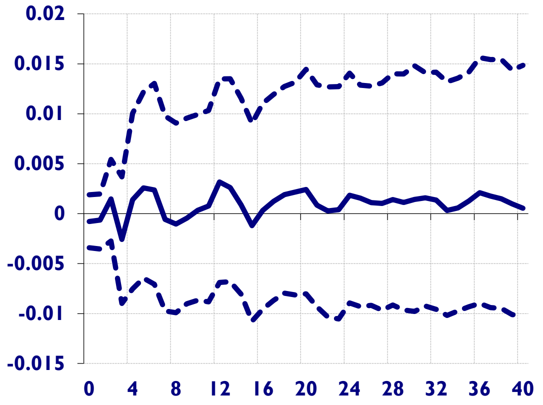

(f) The United Kingdom

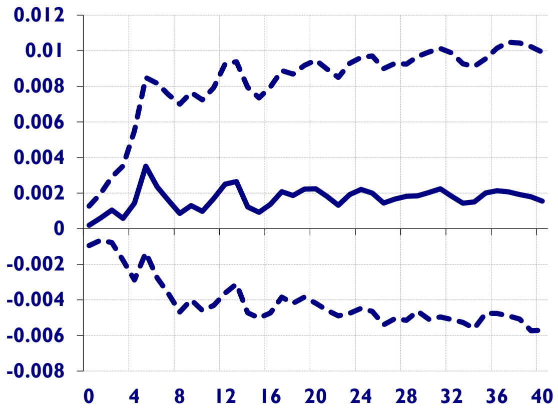

(g) The rest of the EU

Figure S2. Generalized impulse response functions (GIRFs) of intra-EU cheese export prices after a negative one-standard-error shock to France's HICP

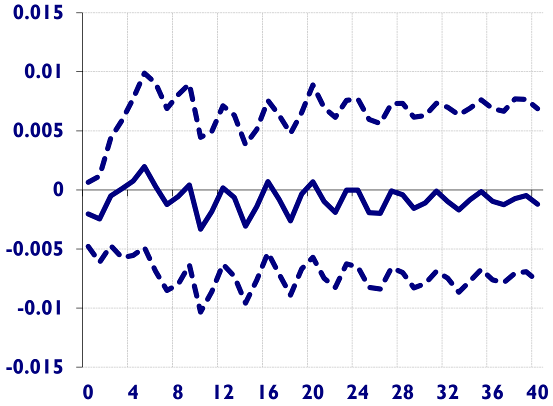

(a) Ireland

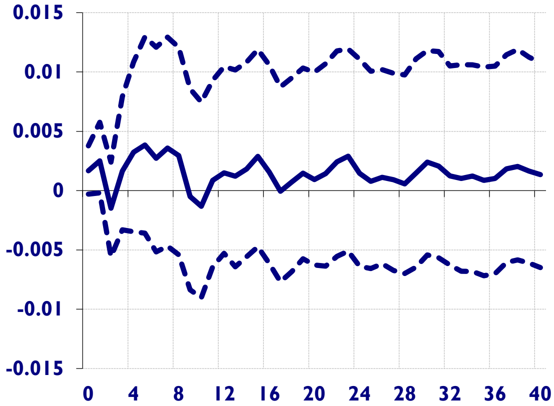

(b) The Netherlands

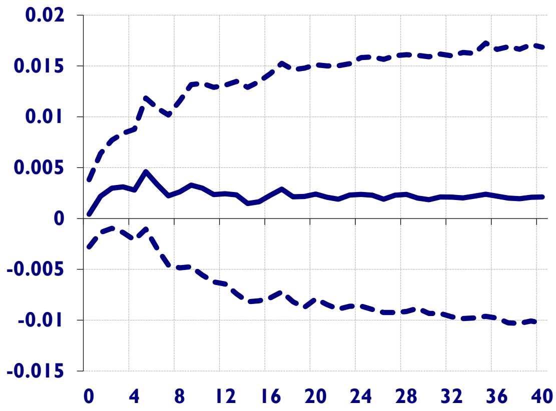

(c) Italy

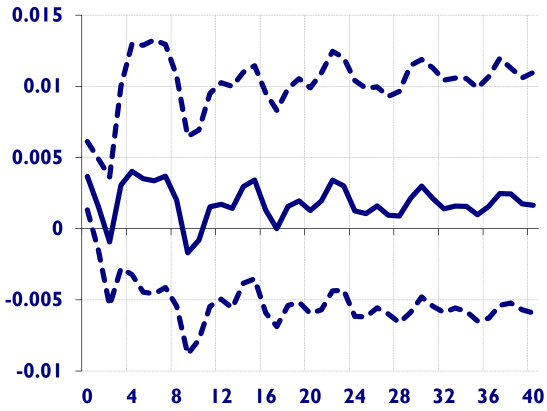

(d) Germany

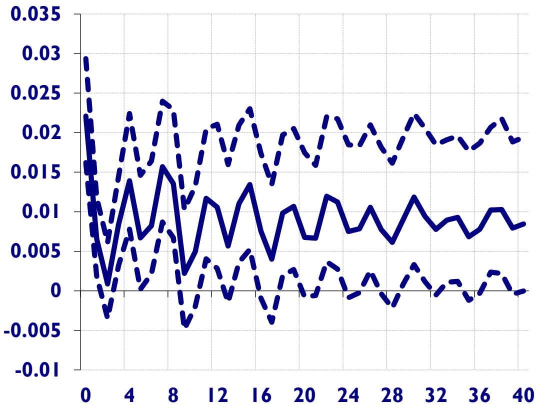

(e) France

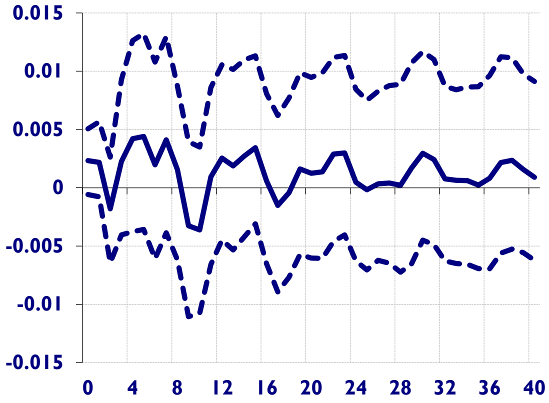

(f) The United Kingdom

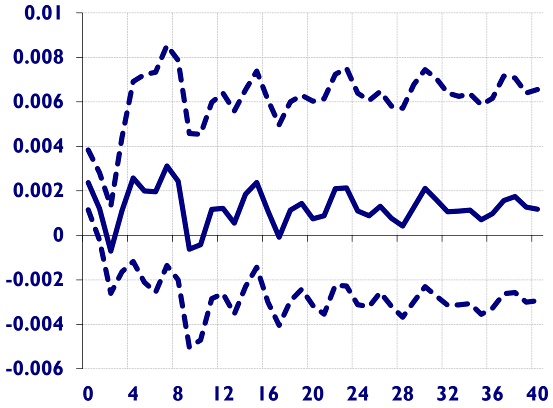

(g) The rest of the EU

Figure S3. Generalized impulse response functions (GIRFs) of intra-EU cheese export prices after a negative one-standard-error shock to France's intra EU cheese export price

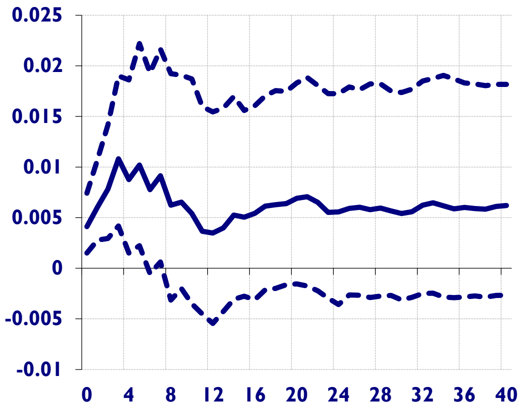

(a) Ireland

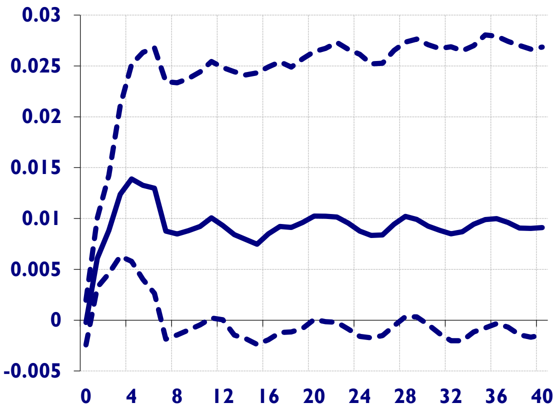

(b) The Netherlands

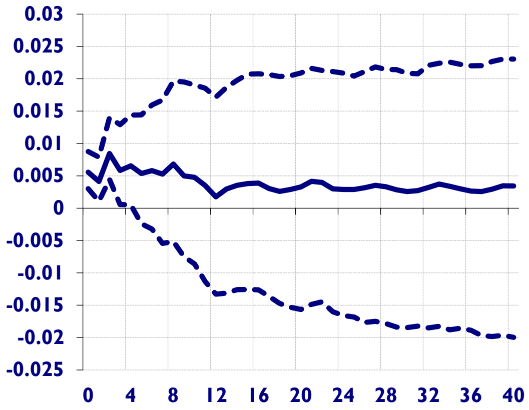

(c) Italy

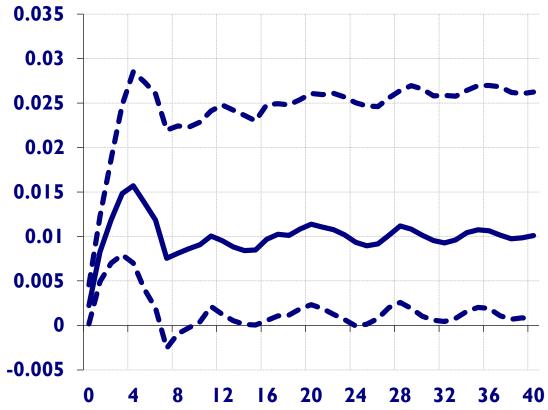

(d) Germany

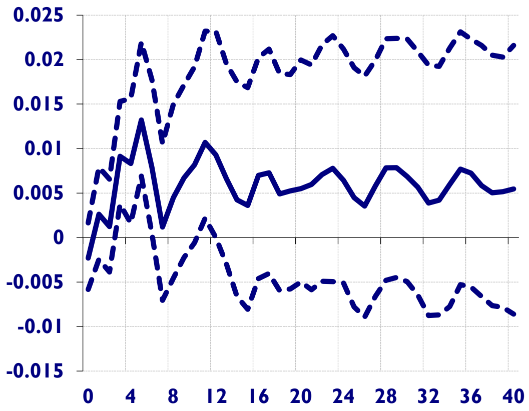

(e) France

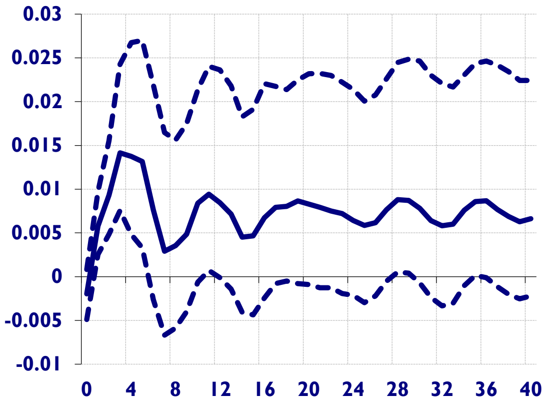

(f) The United Kingdom

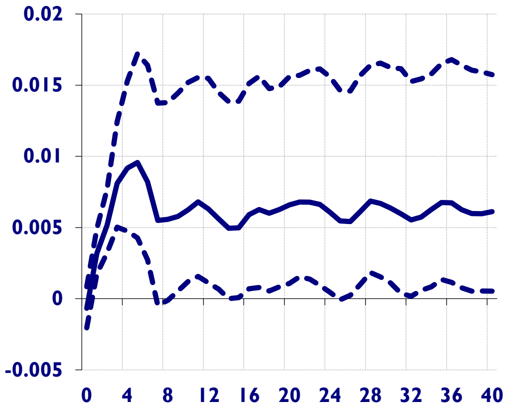

(g) The rest of the EU

Figure S4. Generalized impulse response functions (GIRFs) of intra-EU cheese export prices after a negative one-standard-error shock to France's raw milk price

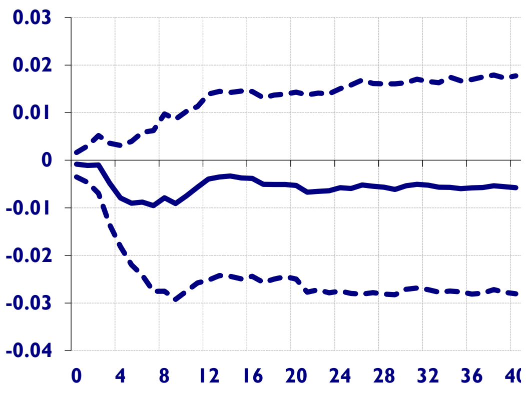

(a) Ireland

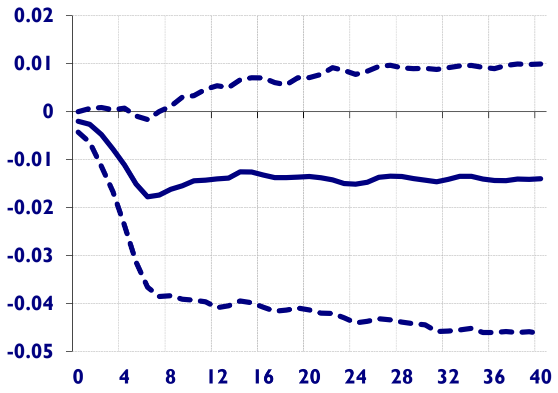

(b) The Netherlands

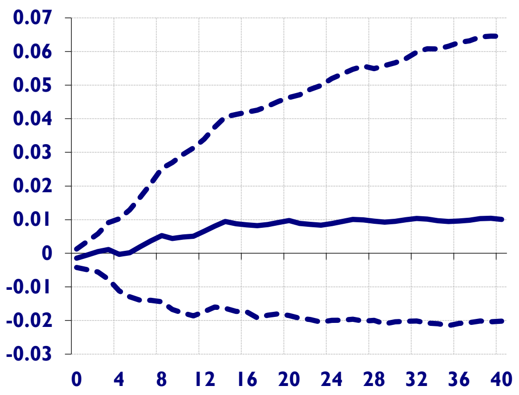

(c) Italy

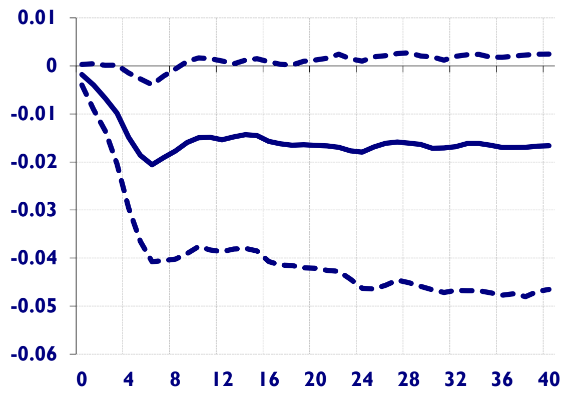

(d) Germany

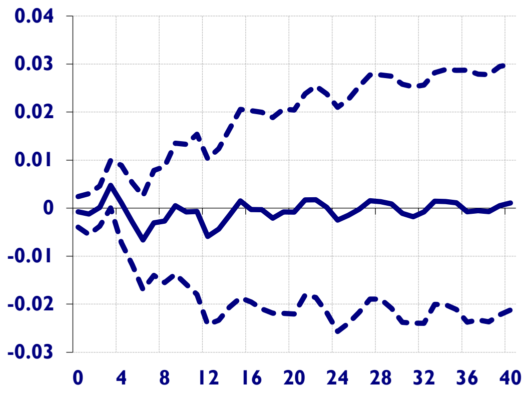

(e) France

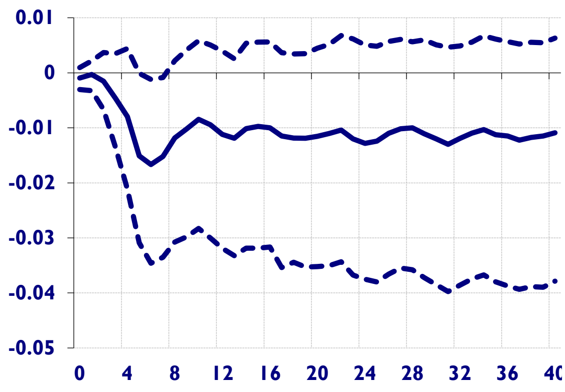

(f) The United Kingdom

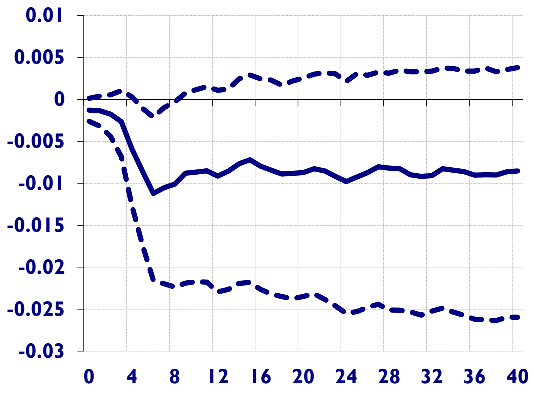

(g) The rest of the EU

Figure S5. Generalized impulse response functions (GIRFs) of intra-EU cheese export prices after a negative one-standard-error shock to Germany's cheese production

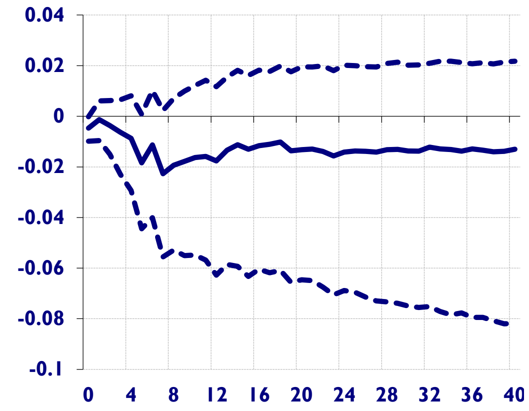

(a) Ireland

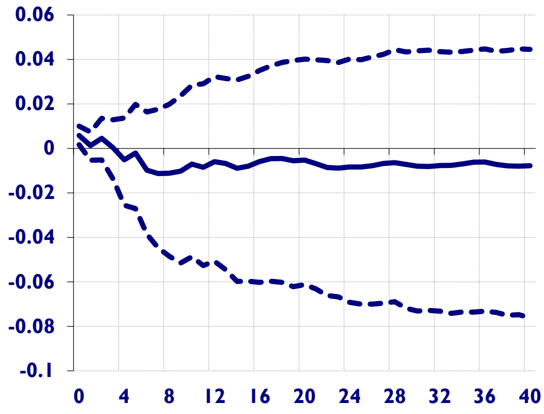

(b) The Netherlands

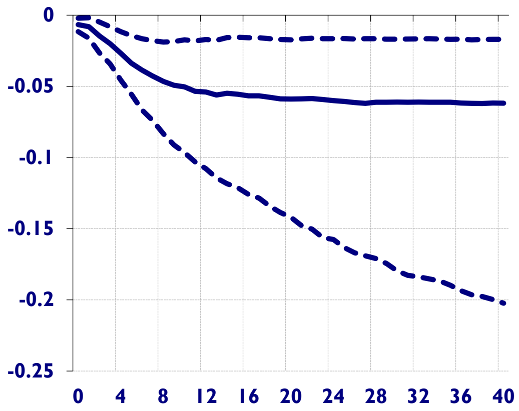

(c) Italy

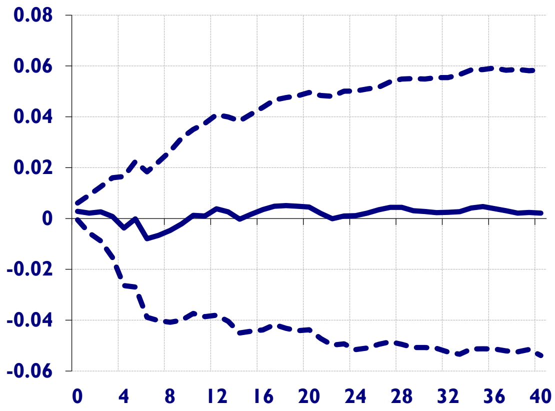

(d) Germany

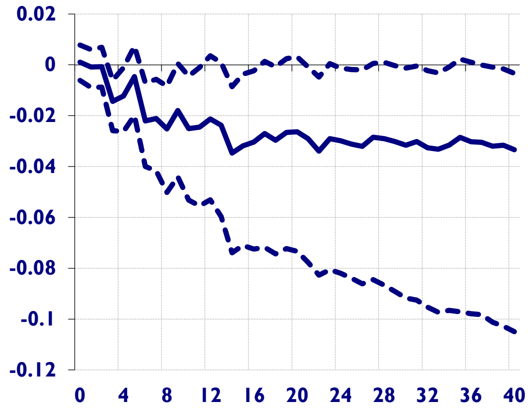

(e) France

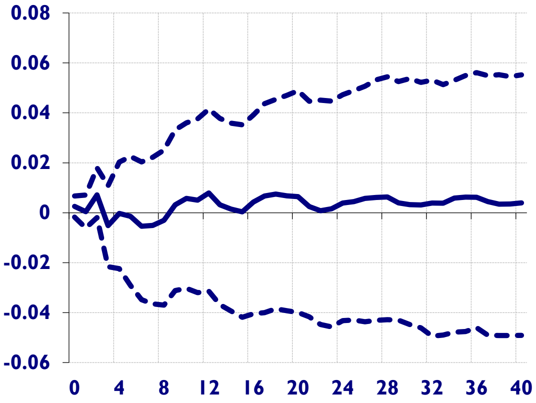

(f) The United Kingdom

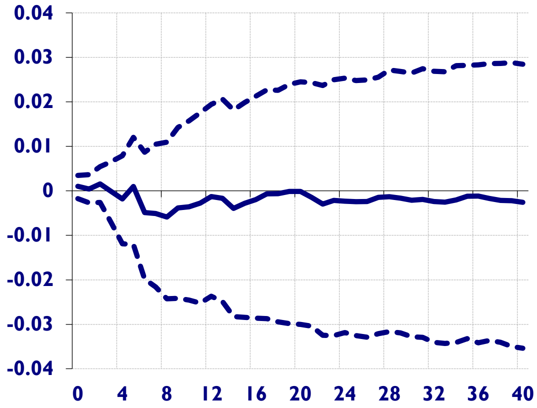

(g) The rest of the EU

Figure S6. Generalized impulse response functions (GIRFs) of intra-EU cheese export prices after a negative one-standard-error shock to Germany's HICP

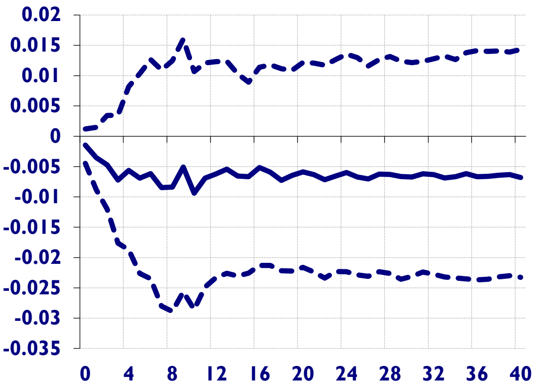

(a) Ireland

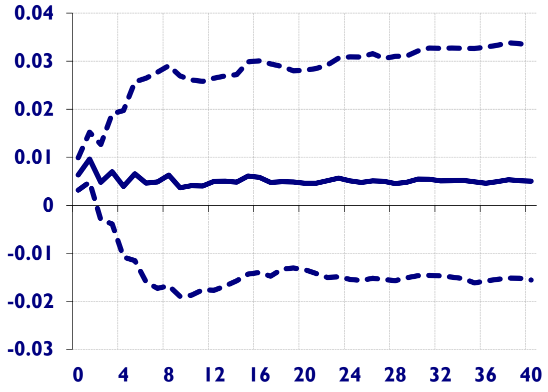

(b) The Netherlands

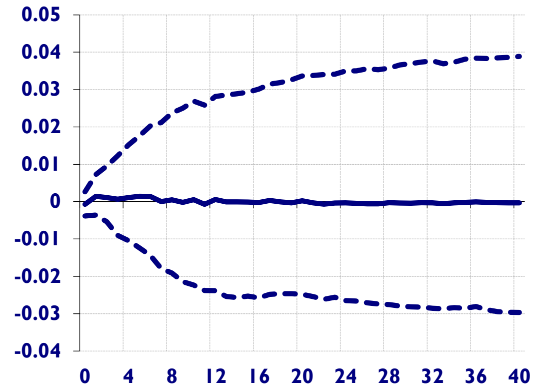

(c) Italy

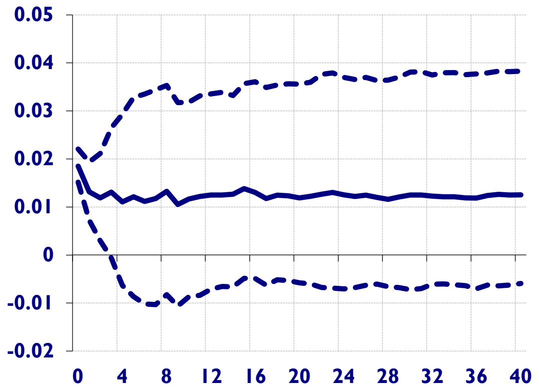

(d) Germany

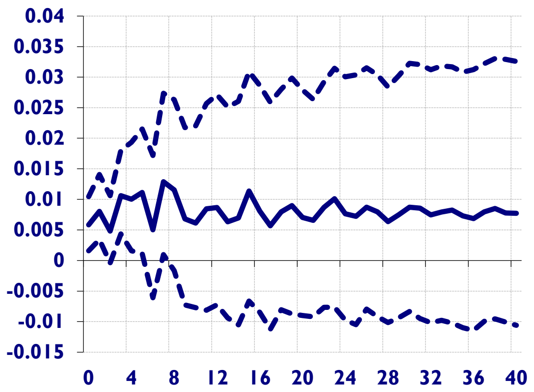

(e) France

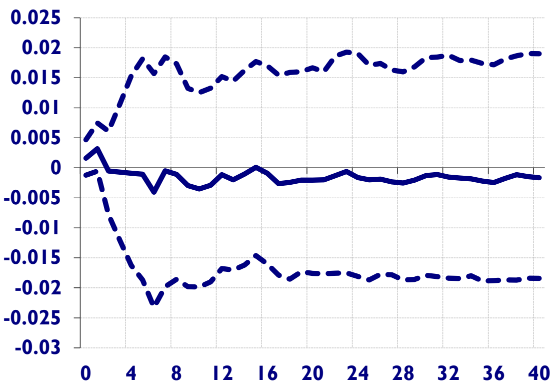

(f) The United Kingdom

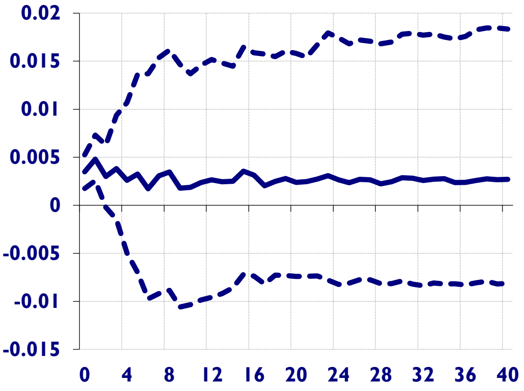

(g) The rest of the EU

Figure S7. Generalized impulse response functions (GIRFs) of intra-EU cheese export prices after a negative one-standard-error shock to Germany's intra EU cheese export prices

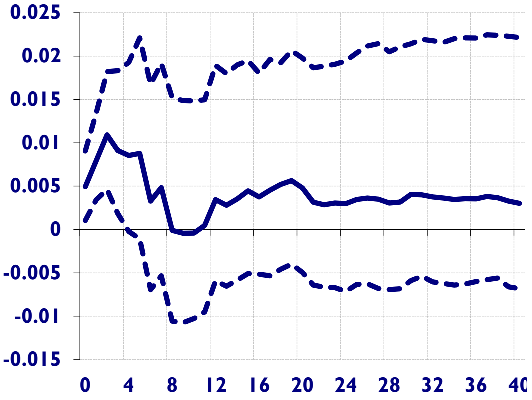

(a) Ireland

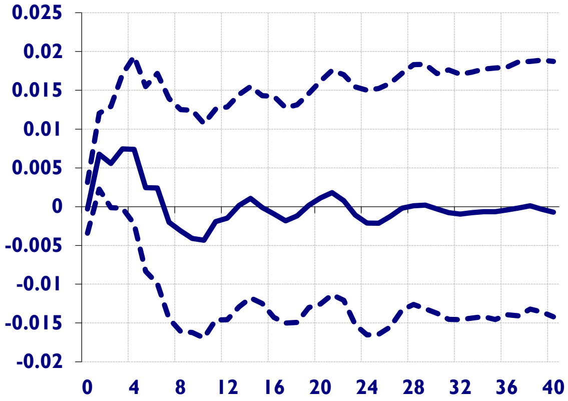

(b) The Netherlands

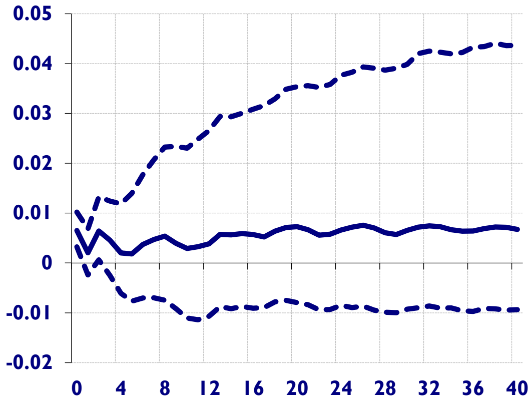

(c) Italy

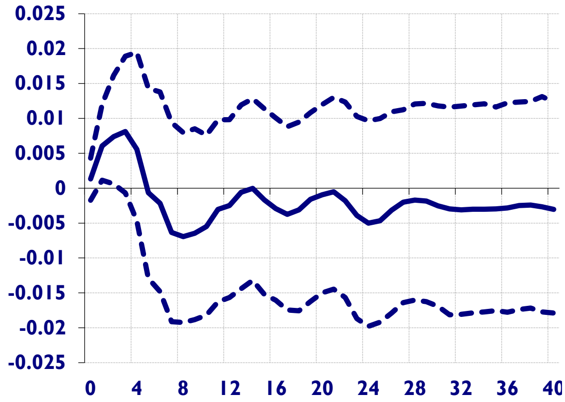

(d) Germany

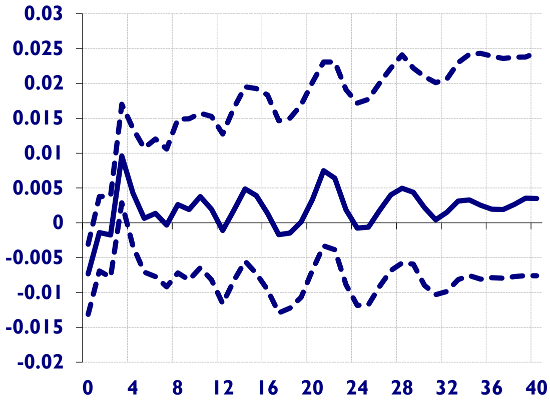

(e) France

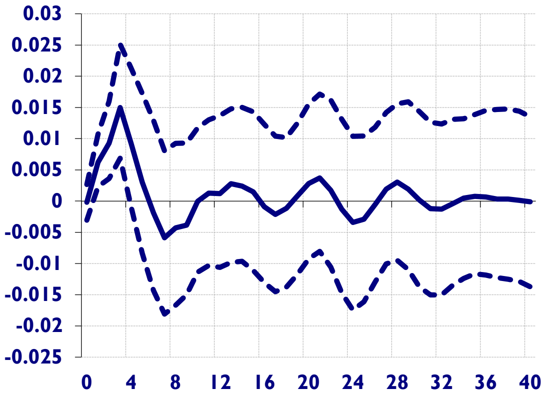

(f) The United Kingdom

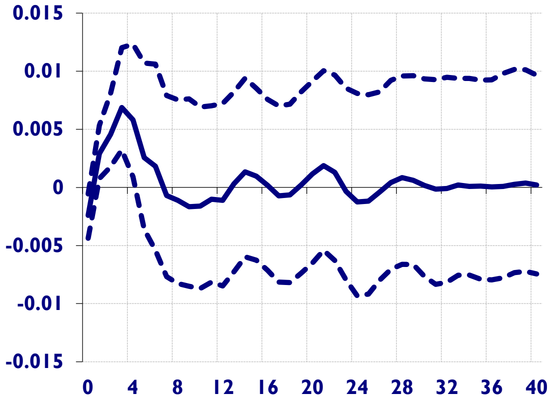

(g) The rest of the EU

Figure S8. Generalized impulse response functions (GIRFs) of intra-EU cheese export prices after a negative one-standard-error shock to Germany's raw milk price

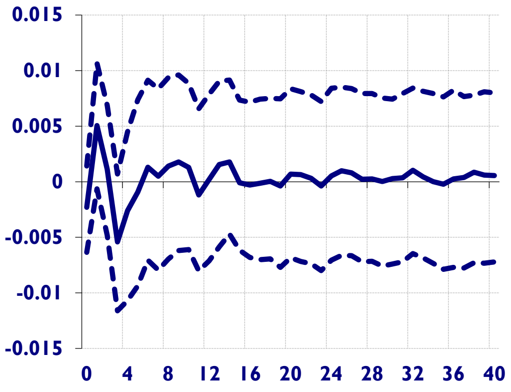

(a) Ireland

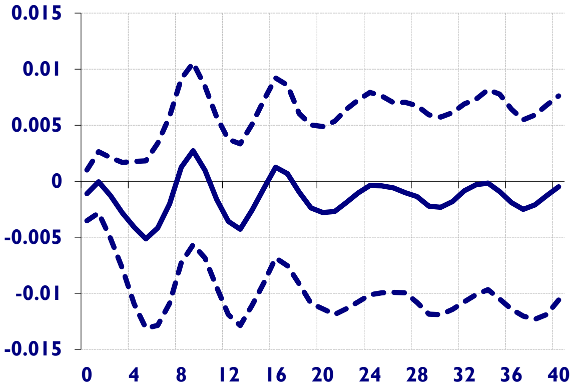

(b) The Netherlands

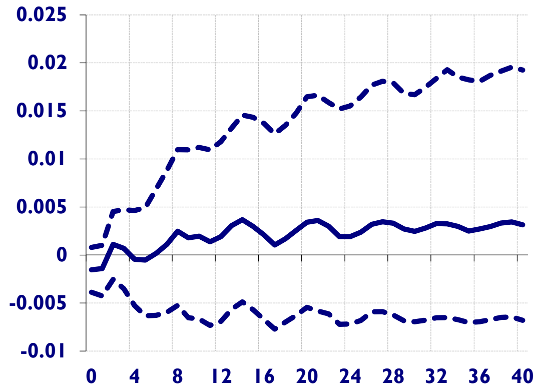

(c) Italy

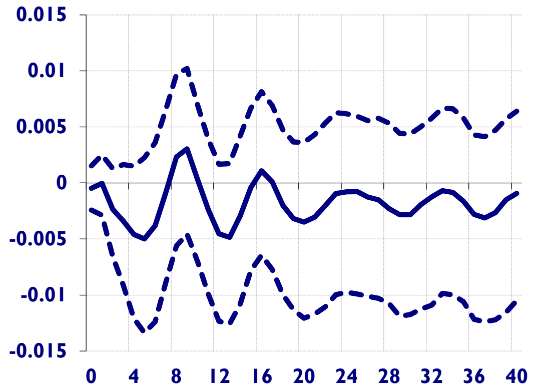

(d) Germany

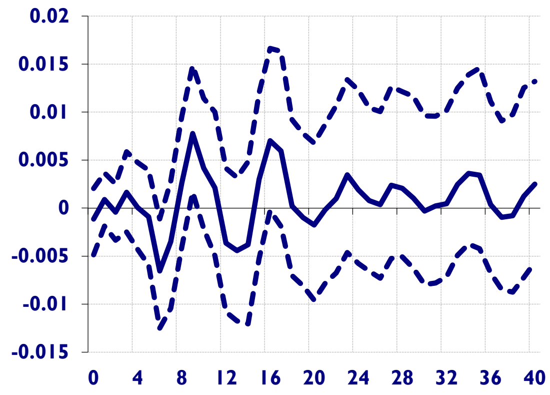

(e) France

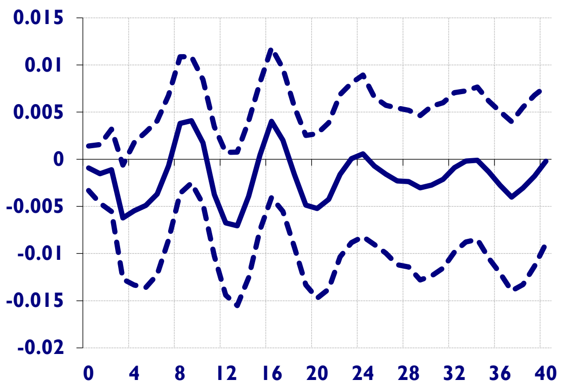

(f) The United Kingdom

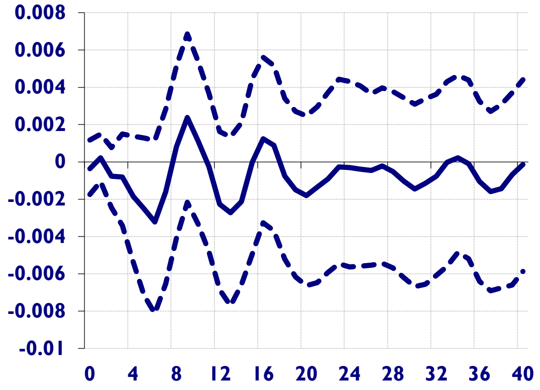

(g) The rest of the EU

Figure S9. Generalized impulse response functions (GIRFs) of intra-EU cheese export prices after a negative one-standard-error shock to Ireland's cheese production

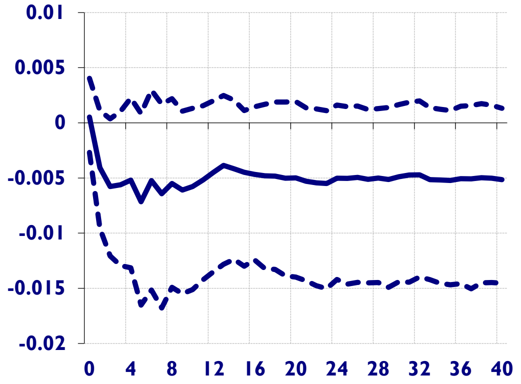

(a) Ireland

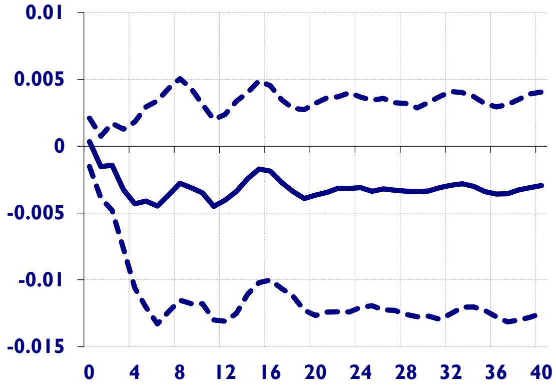

(b) The Netherlands

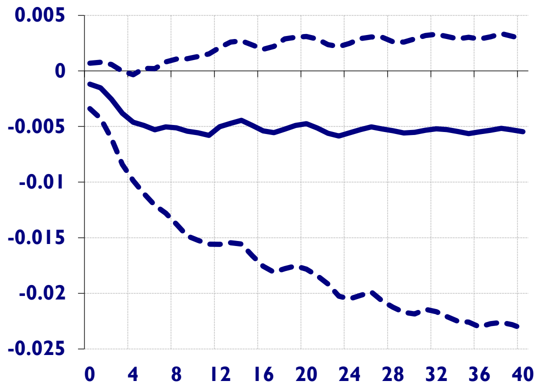

(c) Italy

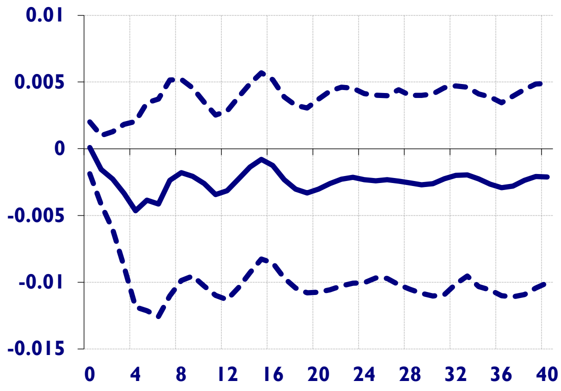

(d) Germany

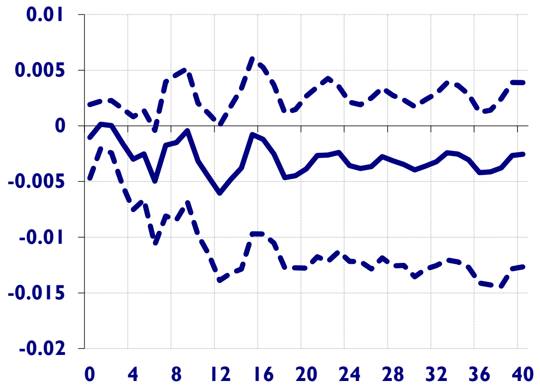

(e) France

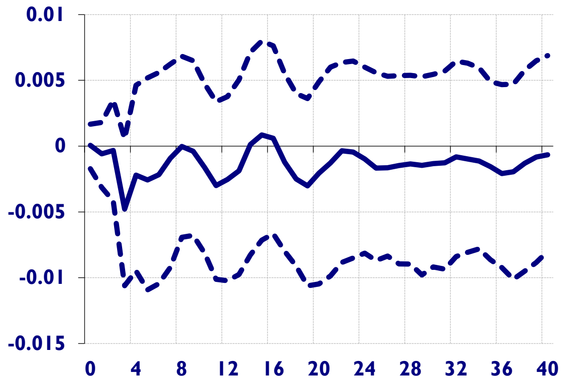

(f) The United Kingdom

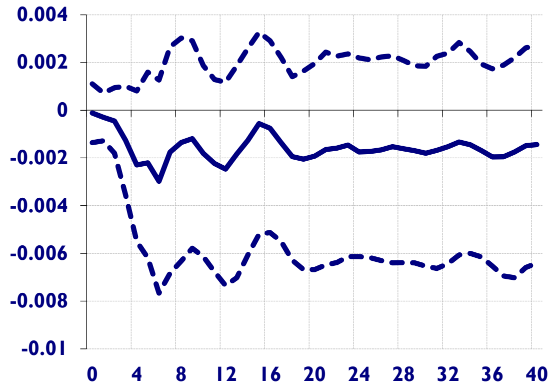

(g) The rest of the EU

Figure S10. Generalized impulse response functions (GIRFs) of intra-EU cheese export prices after a negative one-standard-error shock to Ireland's HICP

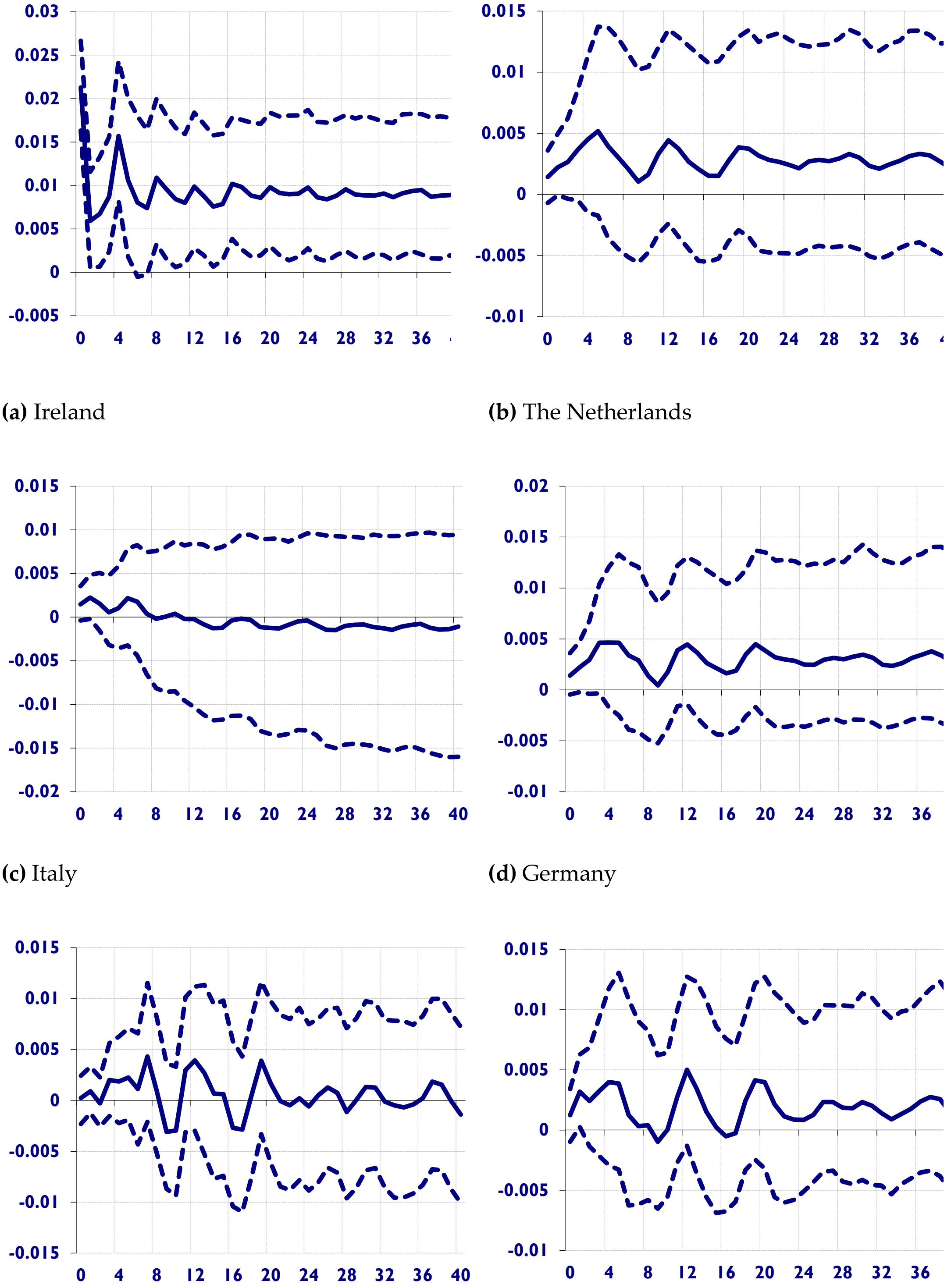

(a) Ireland

(b) The Netherlands

(c) Italy

(d) Germany

(e) France

(f) The United Kingdom

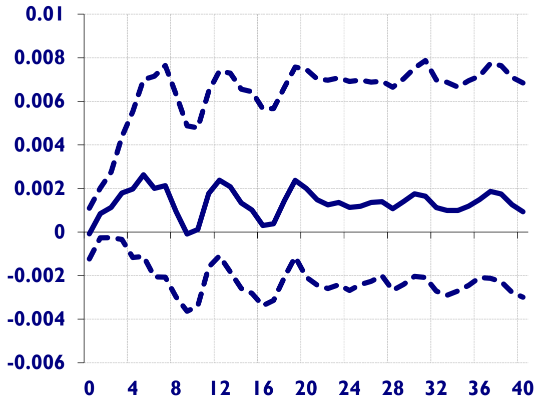

(g) The rest of the EU

Figure S11. Generalized impulse response functions (GIRFs) of intra-EU cheese export prices after a negative one-standard-error shock to Ireland's intra EU cheese export price

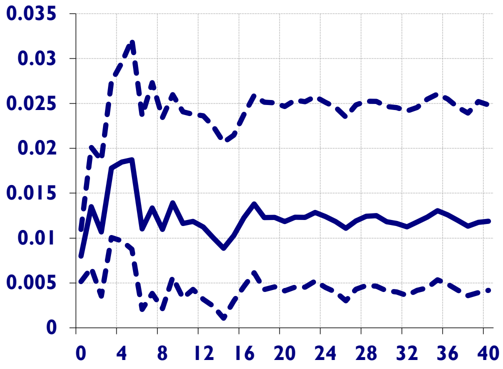

(a) Ireland

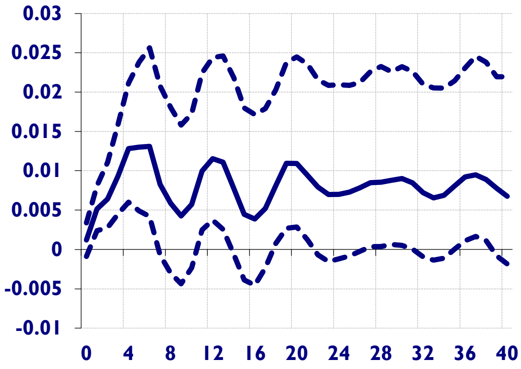

(b) The Netherlands

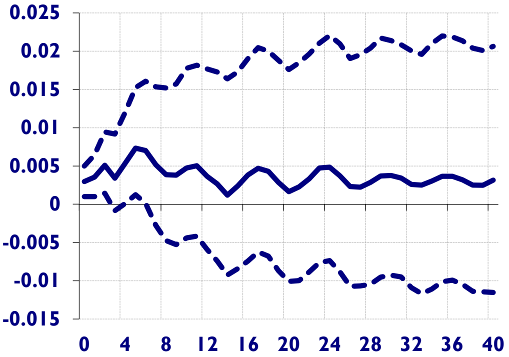

(c) Italy

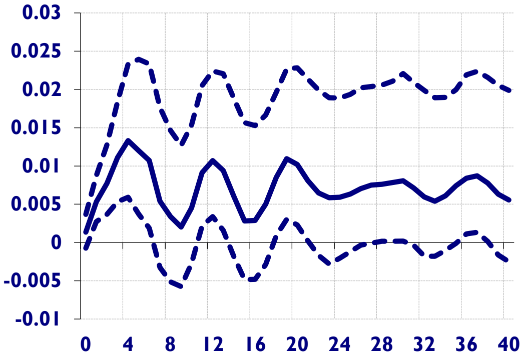

(d) Germany

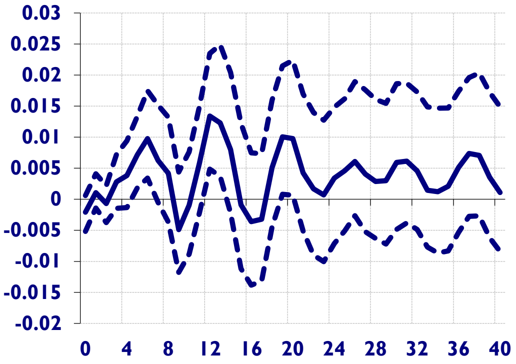

(e) France

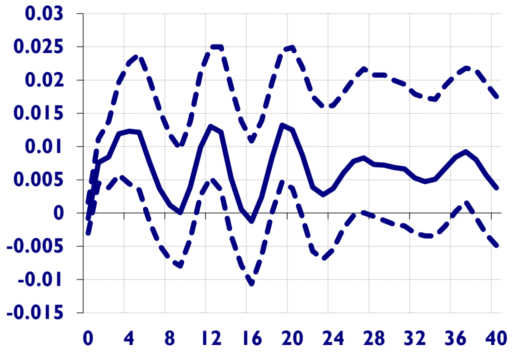

(f) The United Kingdom

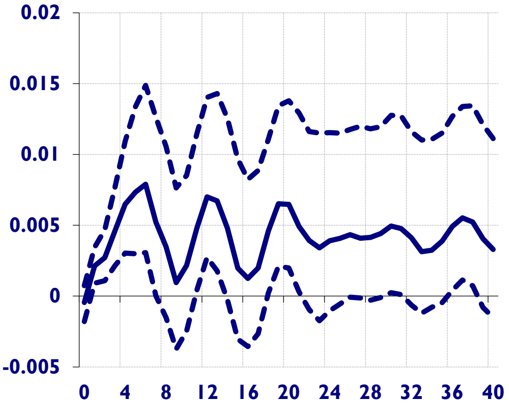

(g) The rest of the EU

Figure S12. Generalized impulse response functions (GIRFs) of intra-EU cheese export prices after a negative one-standard-error shock to Ireland's raw milk price

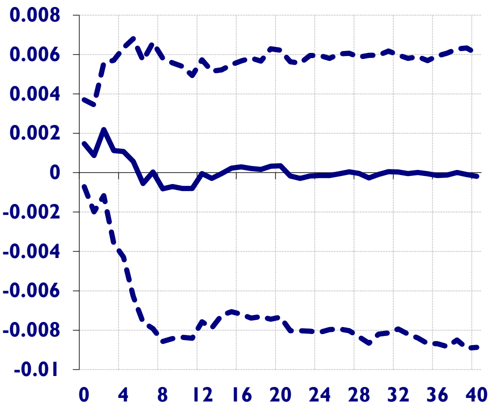

(a) Ireland

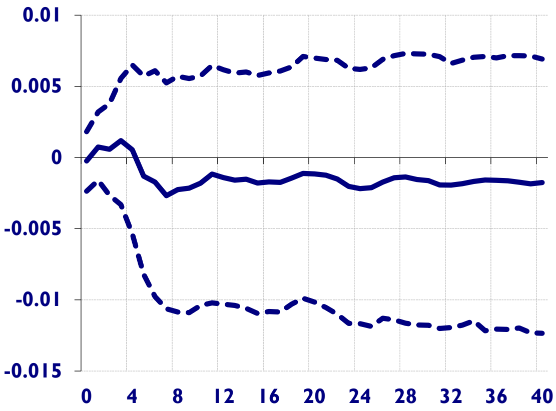

(b) The Netherlands

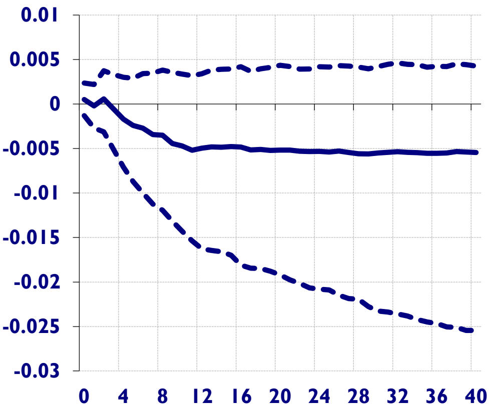

(c) Italy

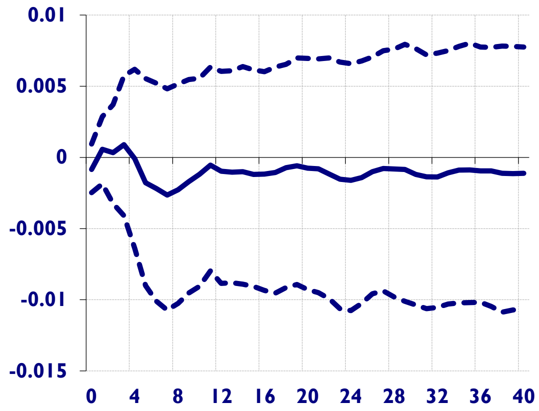

(d) Germany

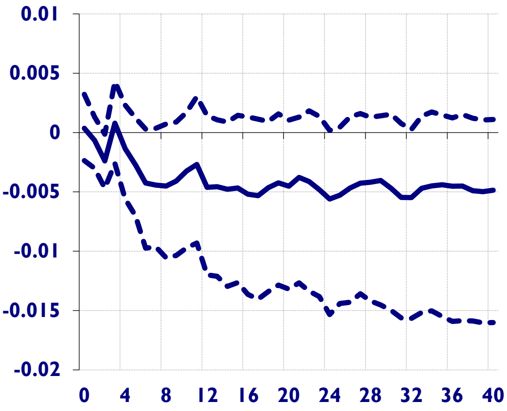

(e) France

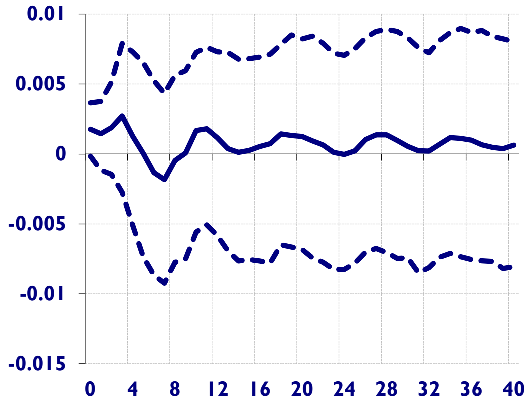

(f) The United Kingdom

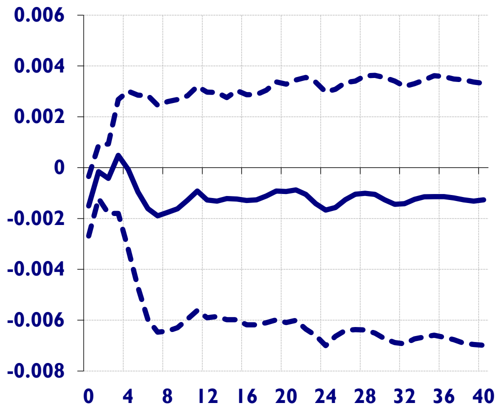

(g) The rest of the EU

Figure S13. Generalized impulse response functions (GIRFs) of intra-EU cheese export prices after a negative one-standard-error shock to Italy's cheese production

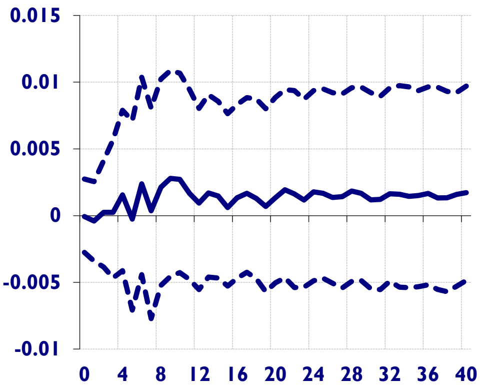

(a) Ireland

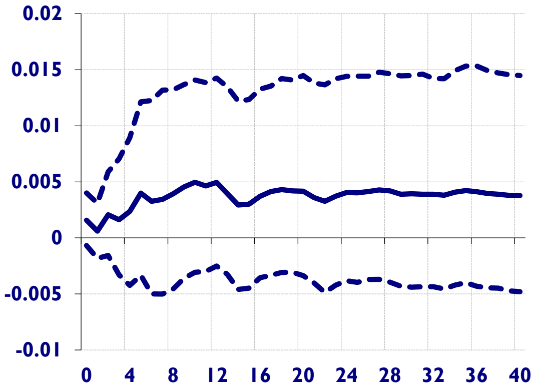

(b) The Netherlands

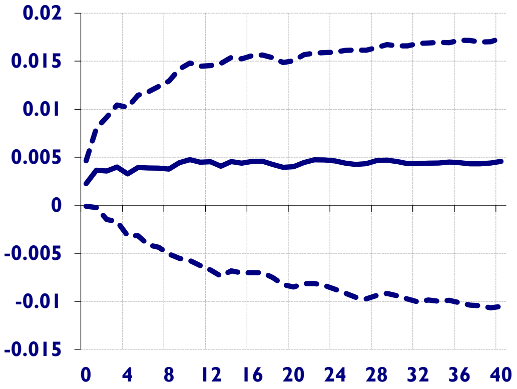

(c) Italy

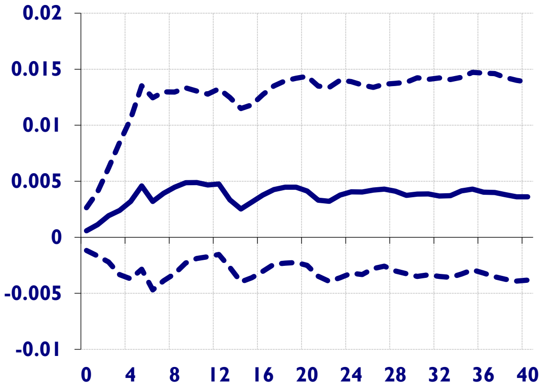

(d) Germany

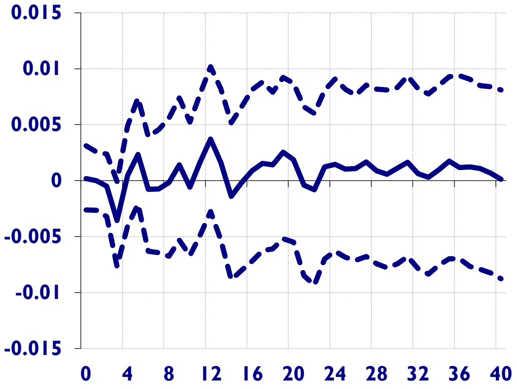

(e) France

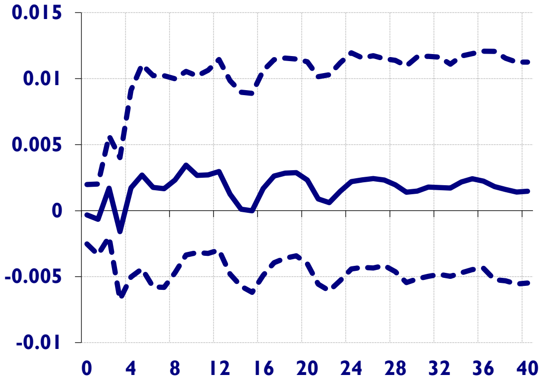

(f) The United Kingdom

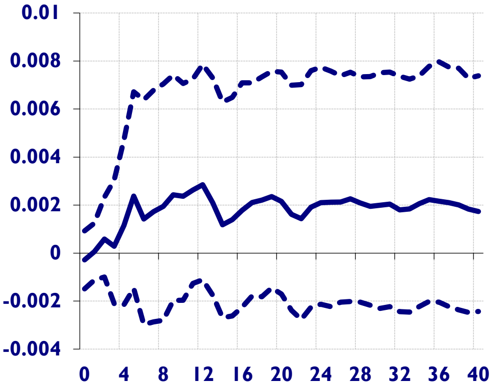

(g) The rest of the EU

Figure S14. Generalized impulse response functions (GIRFs) of intra-EU cheese export prices after a negative one-standard-error shock to Italy's HICP

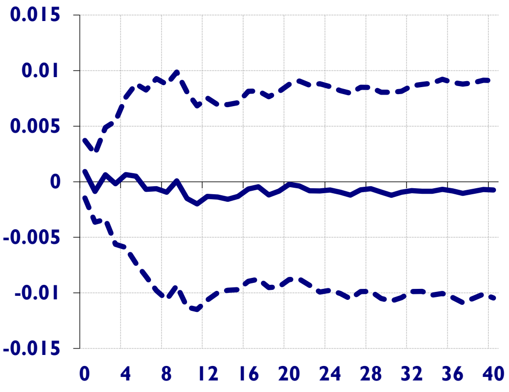

(a) Ireland

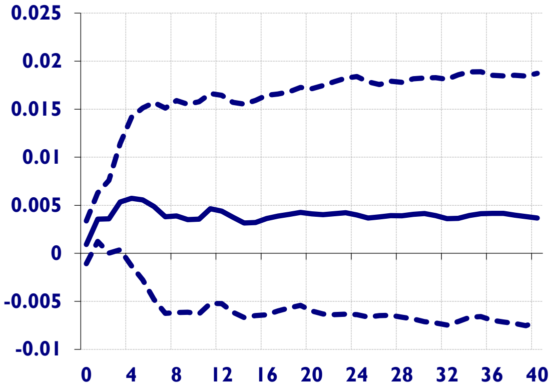

(b) The Netherlands

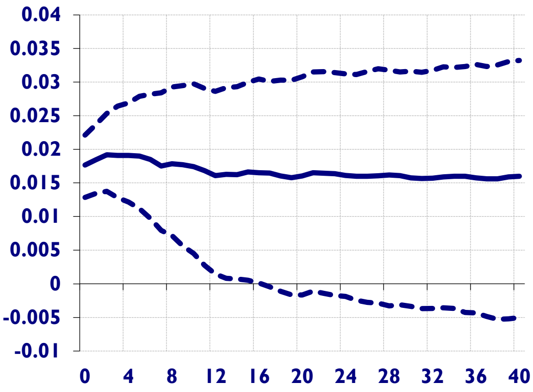

(c) Italy

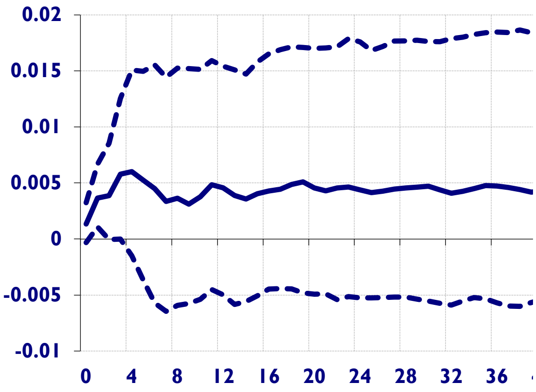

(d) Germany

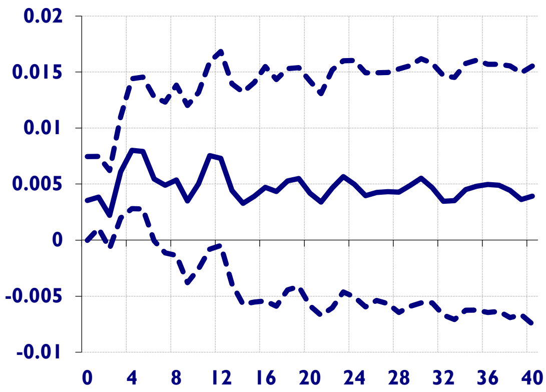

(e) France

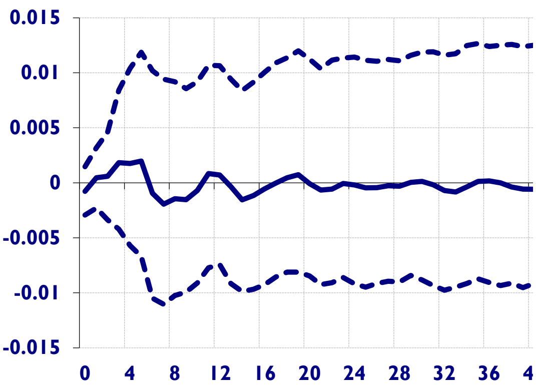

(f) The United Kingdom

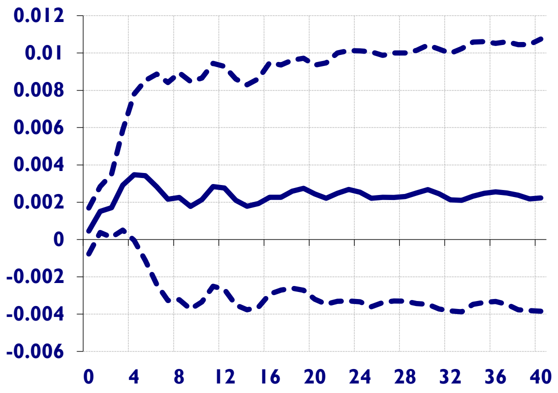

(g) The rest of the EU

Figure S15. Generalized impulse response functions (GIRFs) of intra-EU cheese export prices after a negative one-standard-error shock to Italy's intra EU cheese export price

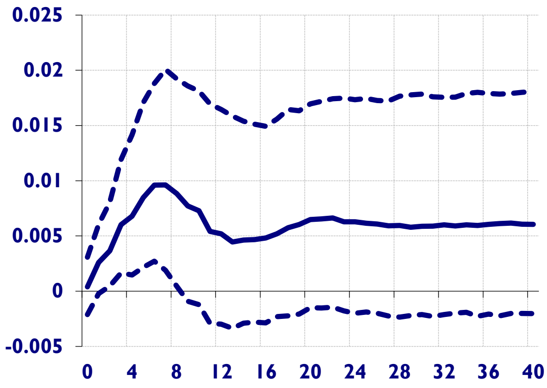

(a) Ireland

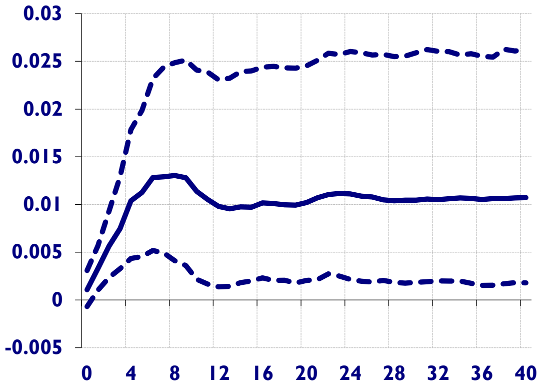

(b) The Netherlands

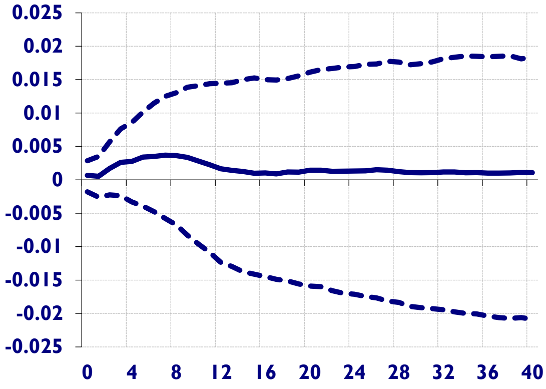

(c) Italy

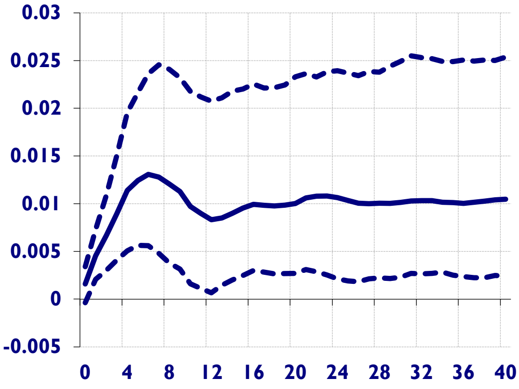

(d) Germany

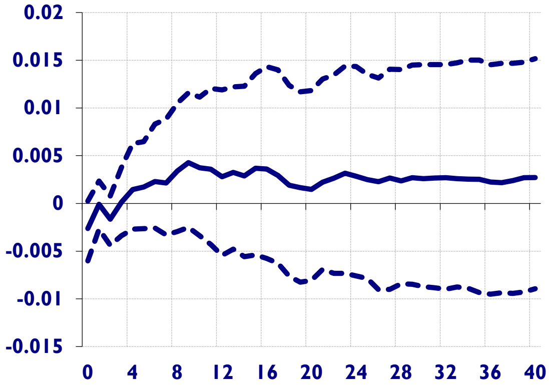

(e) France

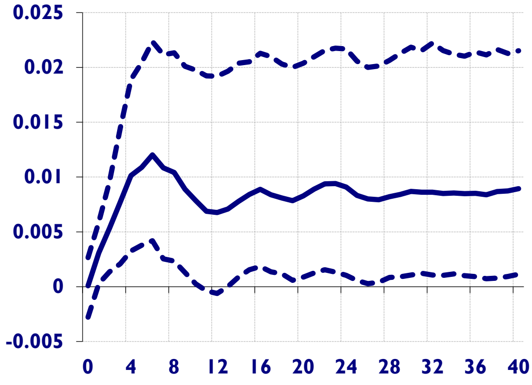

(f) The United Kingdom

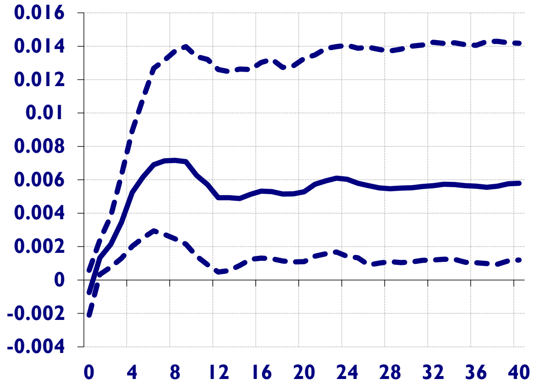

(g) The rest of the EU

Figure S16. Generalized impulse response functions (GIRFs) of intra-EU cheese export prices after a negative one-standard-error shock to Italy's raw milk price

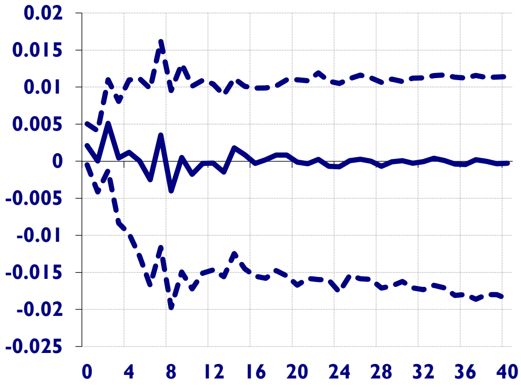

(a) Ireland

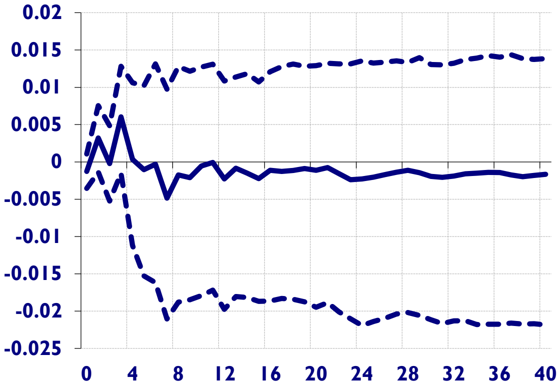

(b) The Netherlands

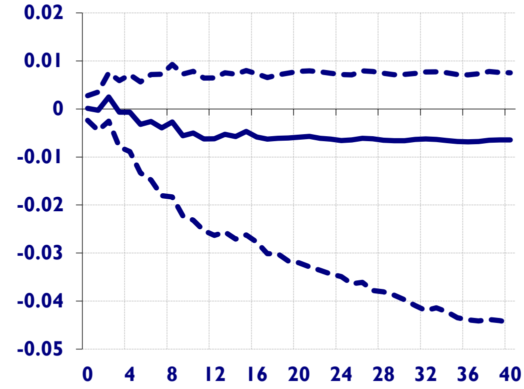

(c) Italy

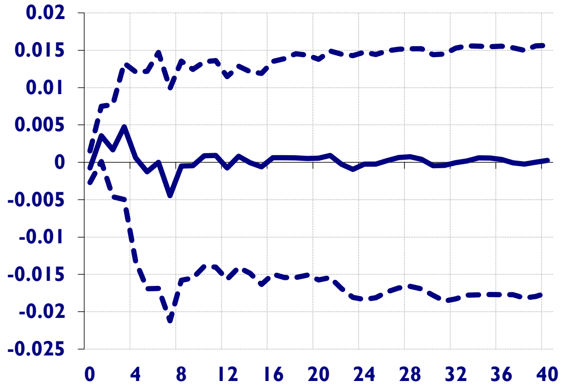

(d) Germany

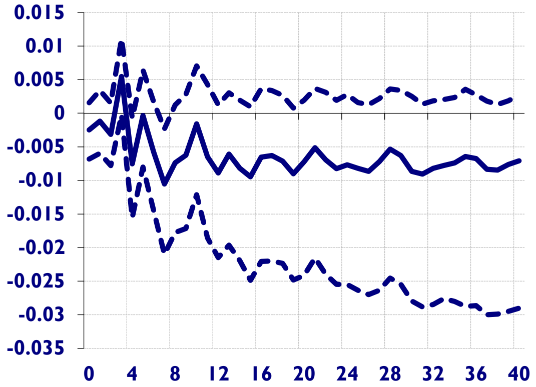

(e) France

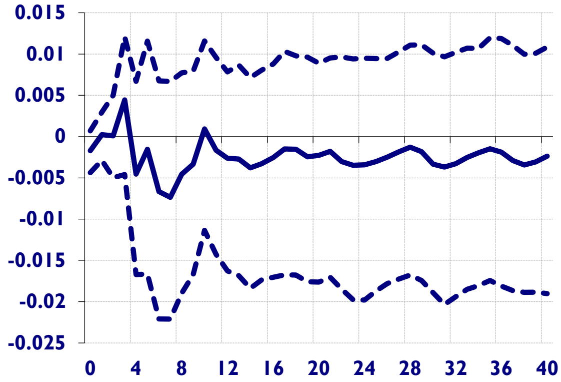

(f) The United Kingdom

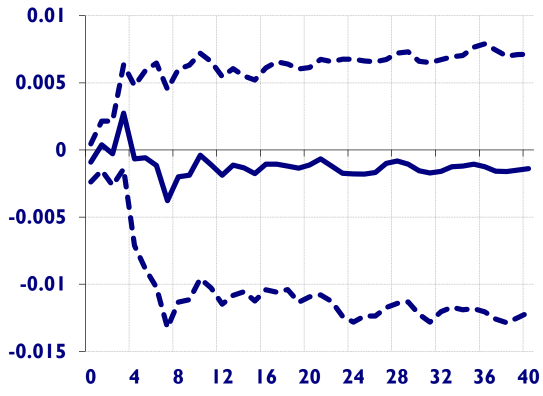

(g) The rest of the EU

Figure S17. Generalized impulse response functions (GIRFs) of intra-EU cheese export prices after a negative one-standard-error shock to the Netherlands' cheese production

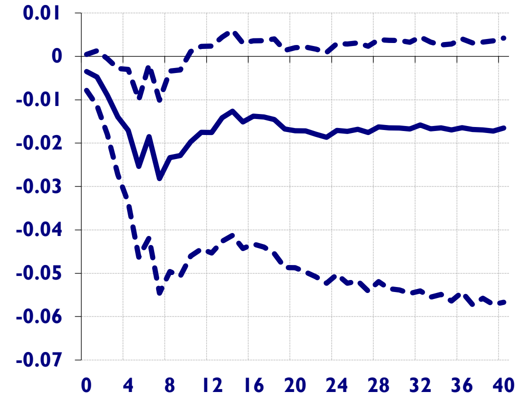

(a) Ireland

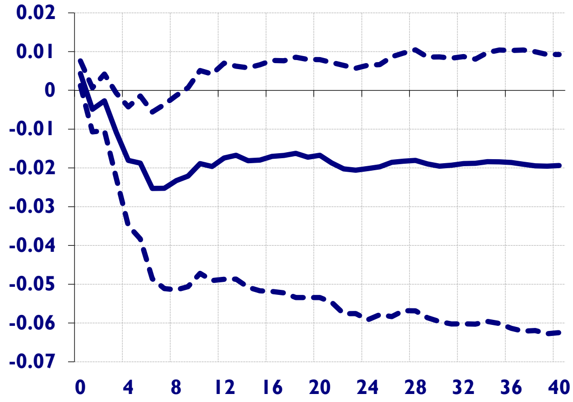

(b) The Netherlands

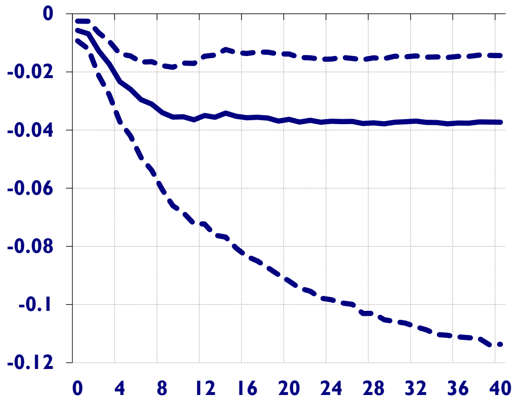

(c) Italy

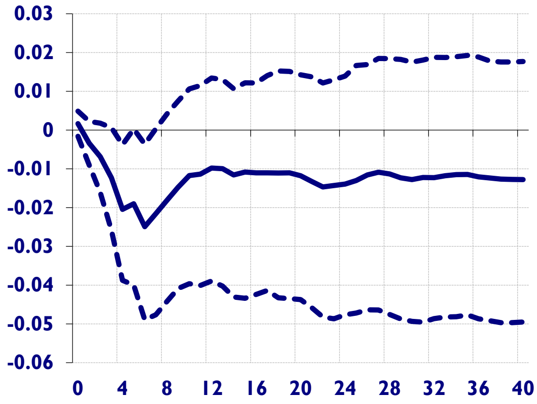

(d) Germany

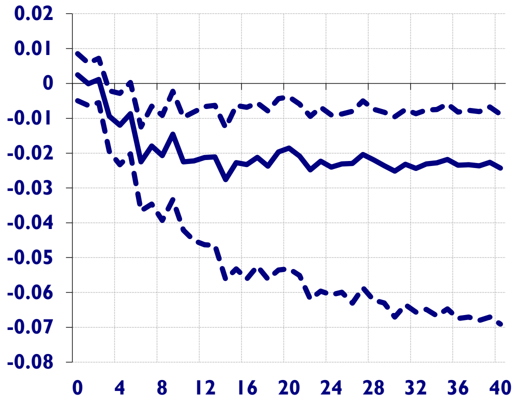

(e) France

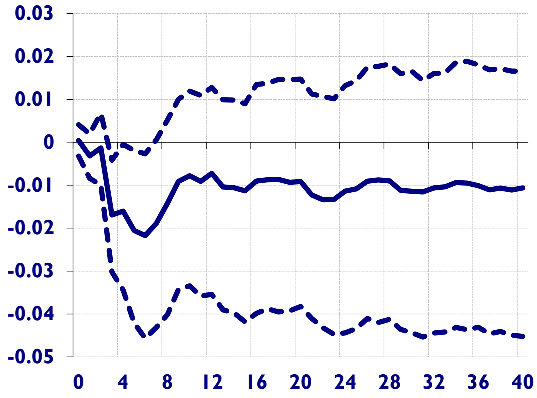

(f) The United Kingdom

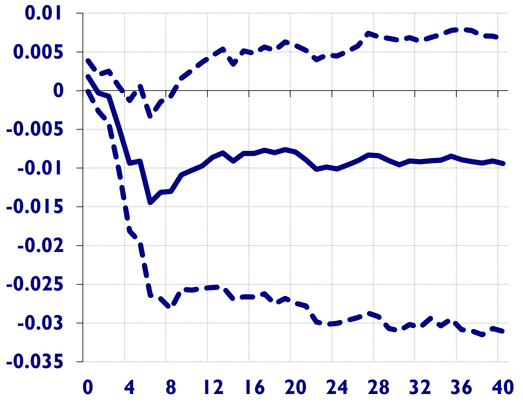

(g) The rest of the EU

Figure S18. Generalized impulse response functions (GIRFs) of intra-EU cheese export prices after a negative one-standard-error shock to the Netherlands' HICP

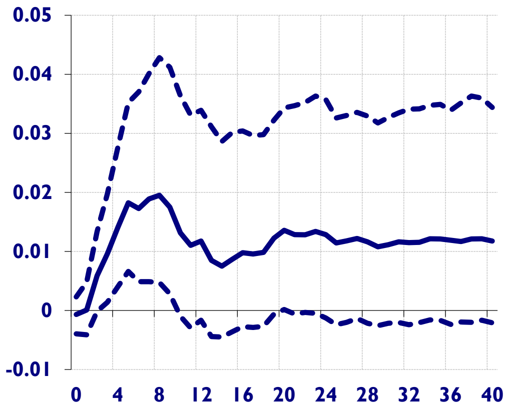

(a) Ireland

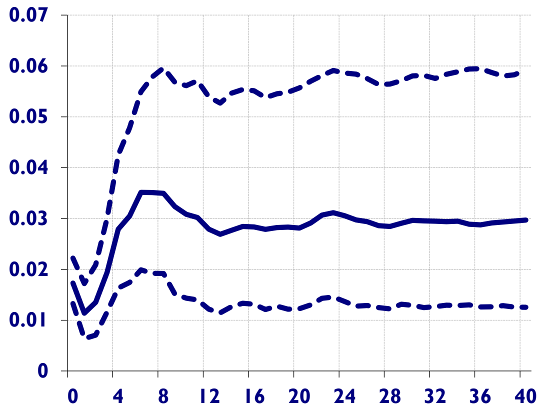

(b) The Netherlands

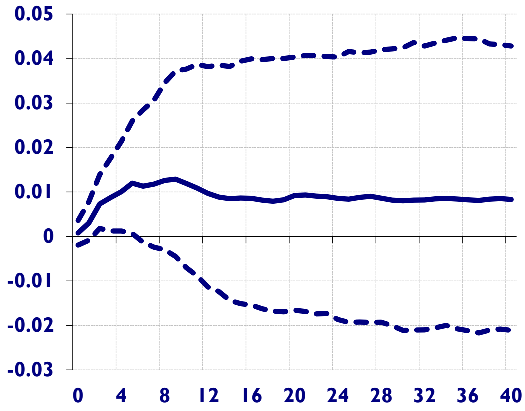

(c) Italy

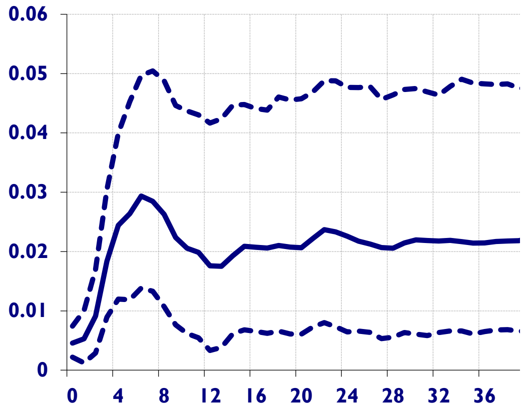

(d) Germany

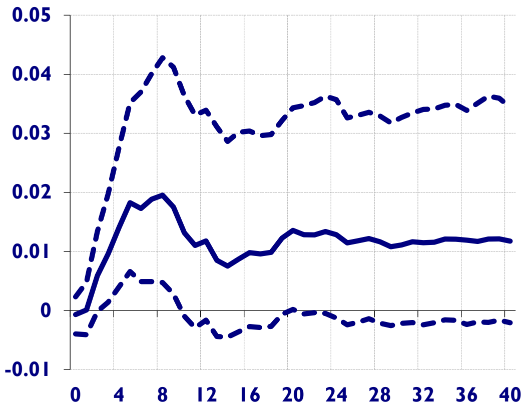

(e) France

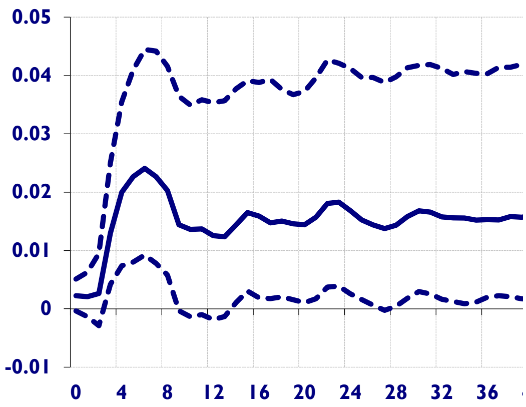

(f) The United Kingdom

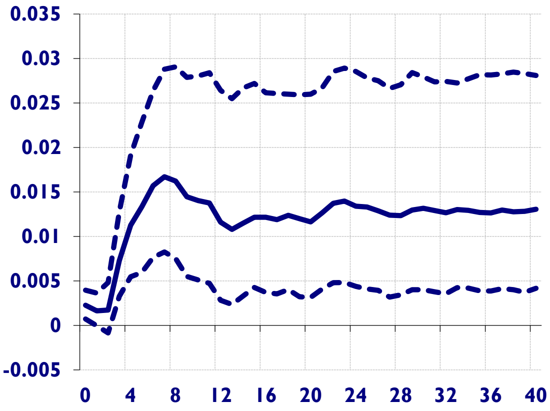

(g) The rest of the EU

Figure S19. Generalized impulse response functions (GIRFs) of intra-EU cheese export prices after a negative one-standard-error shock to the Netherlands' Intra EU export price

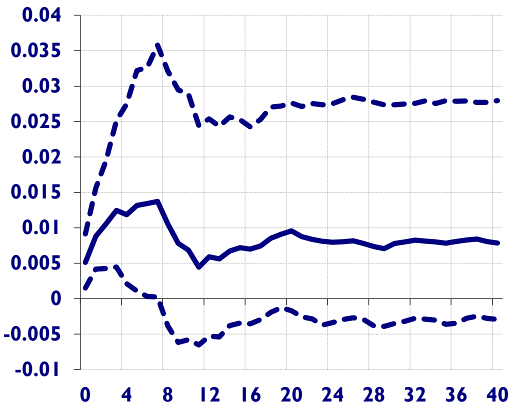

(a) Ireland

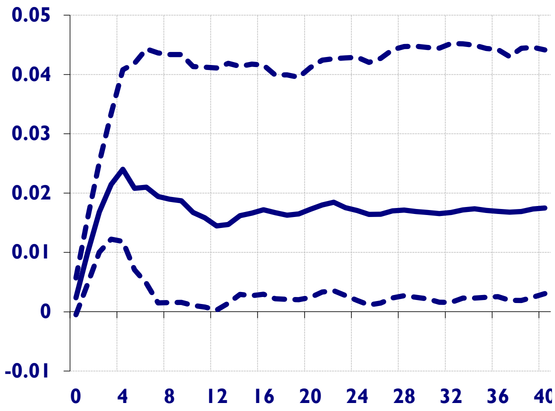

(b) The Netherlands

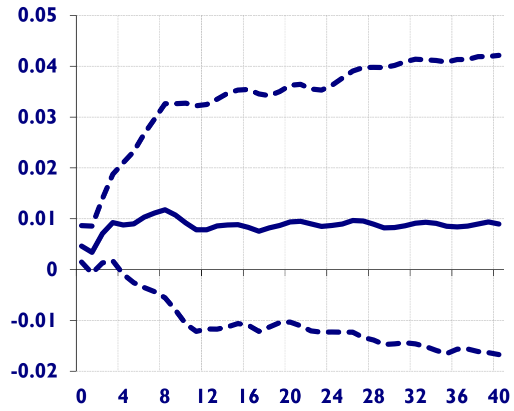

(c) Italy

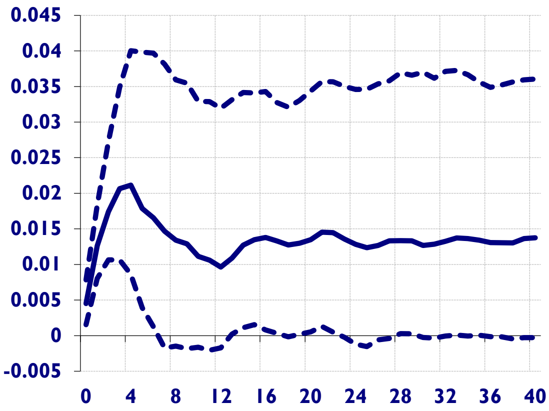

(d) Germany

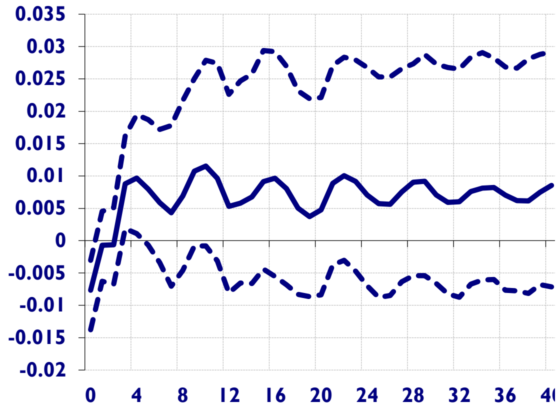

(e) France

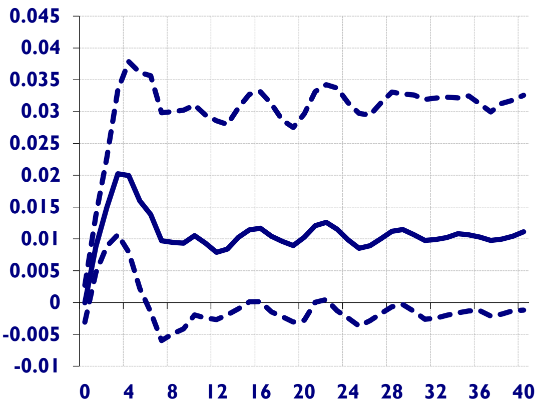

(f) The United Kingdom

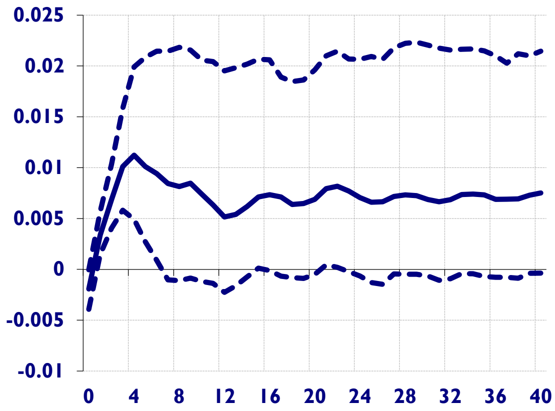

(g) The rest of the EU

Figure S20. Generalized impulse response functions (GIRFs) of intra-EU cheese export prices after a negative one-standard-error shock to the Netherlands' raw milk price

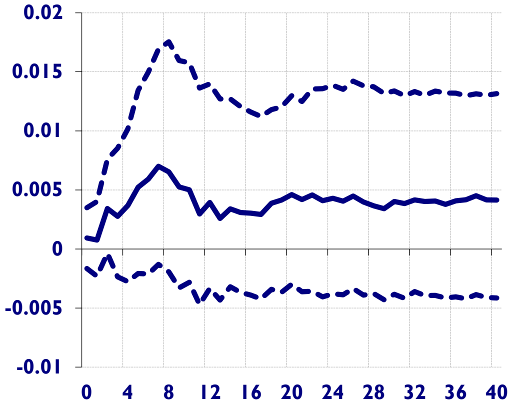

(a) Ireland

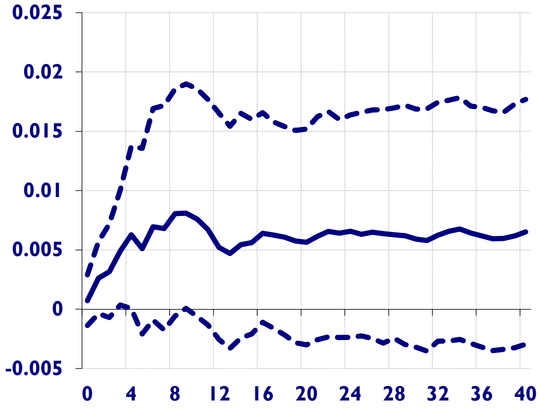

(b) The Netherlands

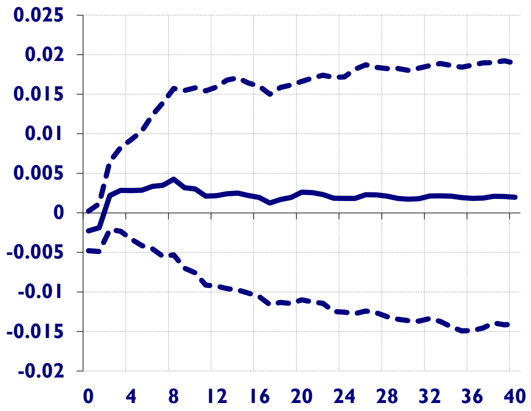

(c) Italy

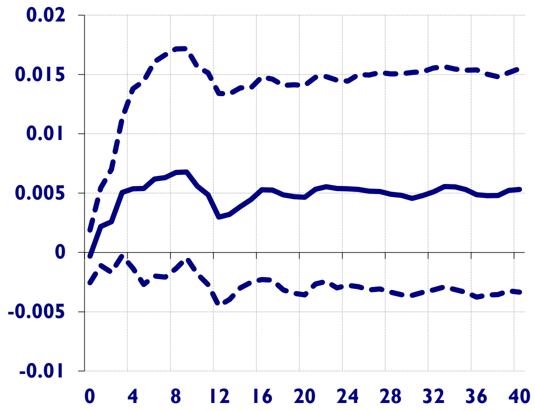

(d) Germany

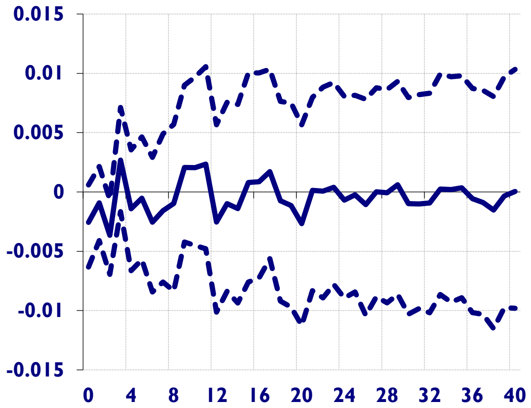

(e) France

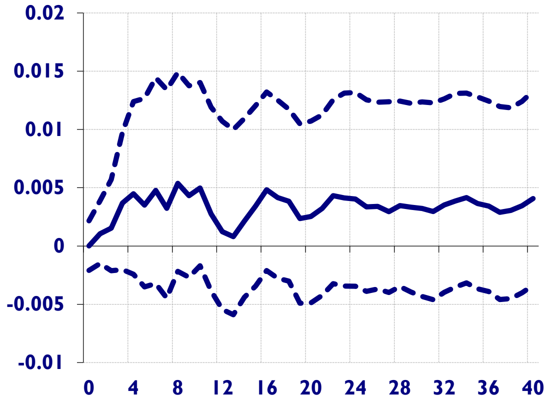

(f) The United Kingdom

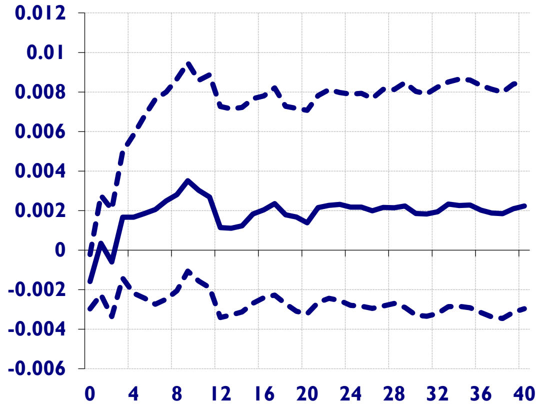

(g) The rest of the EU

Figure S21. Generalized impulse response functions (GIRFs) of intra-EU cheese export prices after a negative one-standard-error shock to the REU′ cheese production

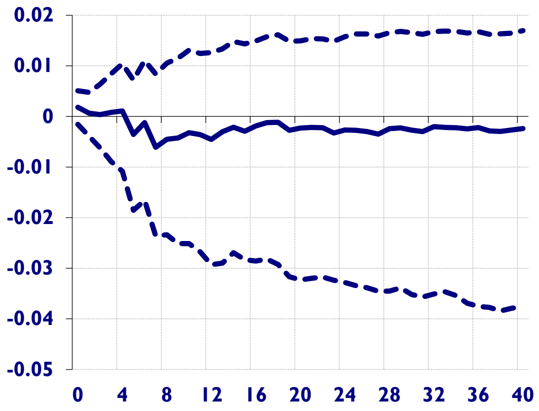

(a) Ireland

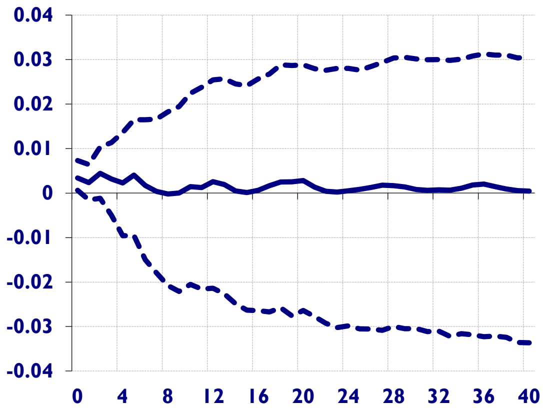

(b) The Netherlands

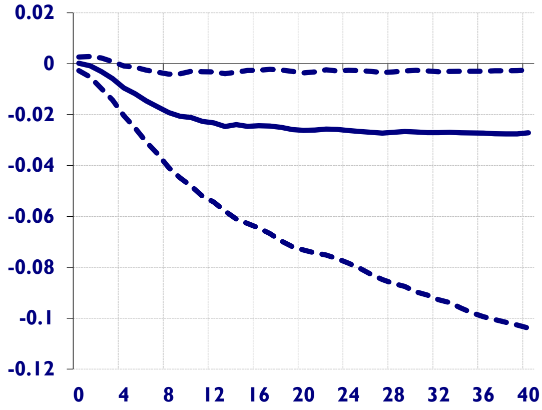

(c) Italy

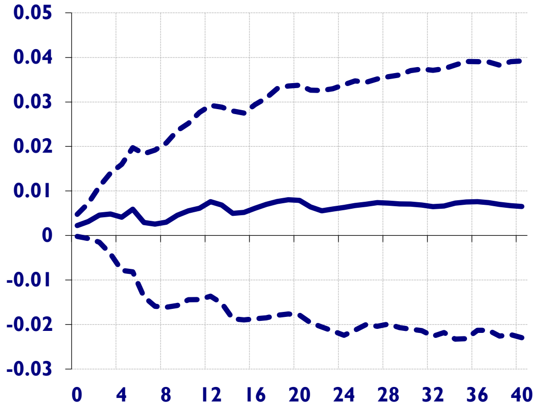

(d) Germany

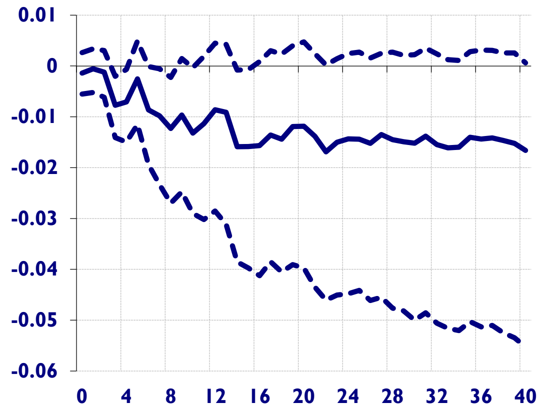

(e) France

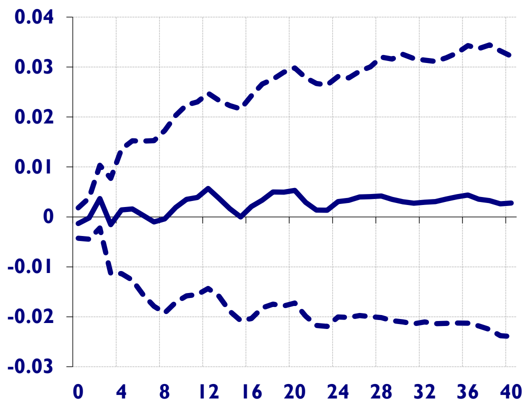

(f) The United Kingdom

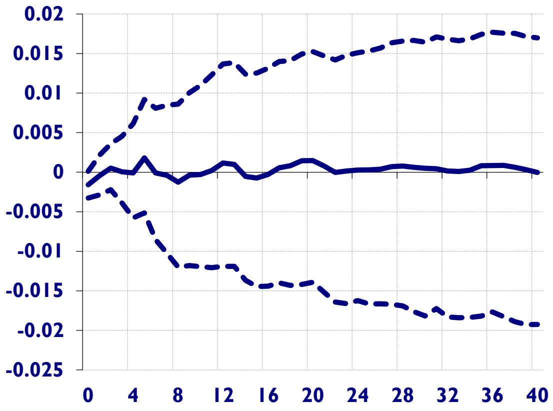

(g) The rest of the EU

Figure S22. Generalized impulse response functions (GIRFs) of intra-EU cheese export prices after a negative one-standard-error shock to the REU′ HICP

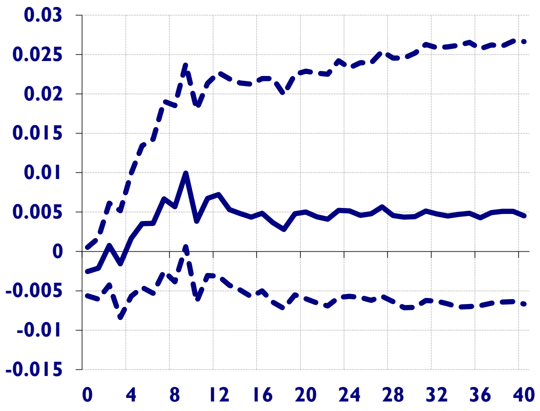

(a) Ireland

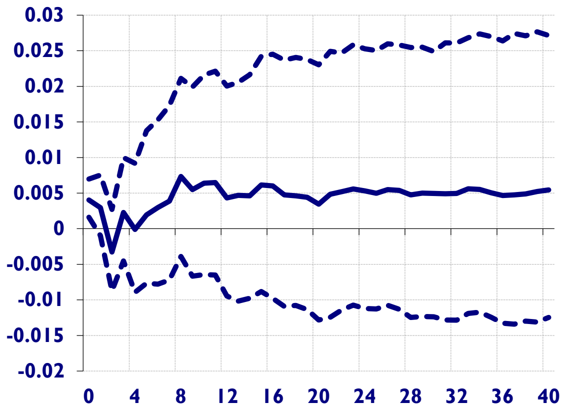

(b) The Netherlands

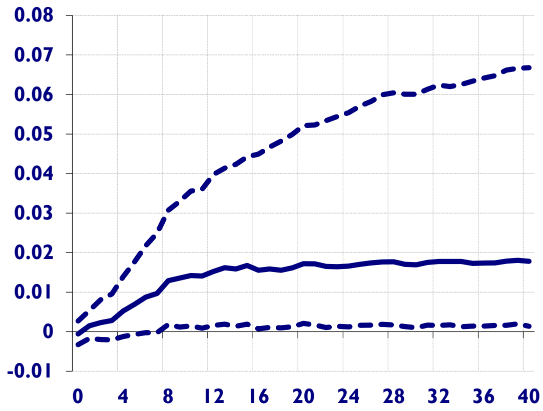

(c) Italy

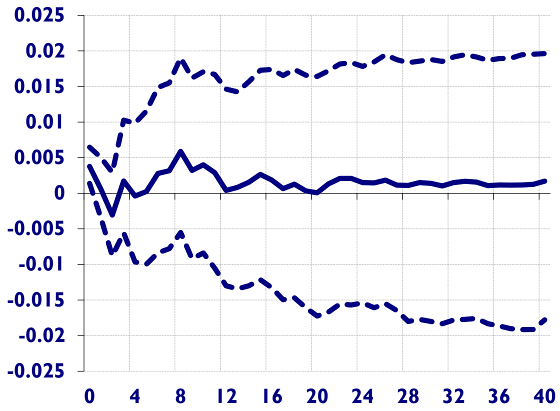

(d) Germany

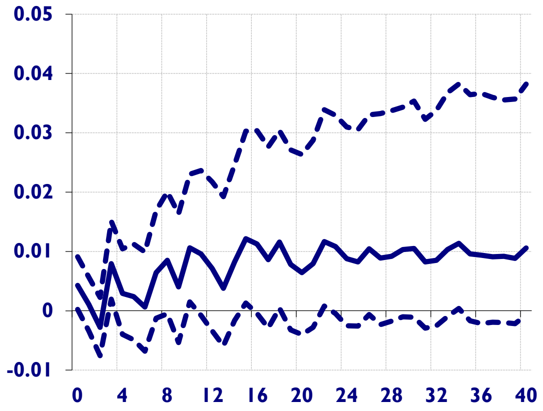

(e) France

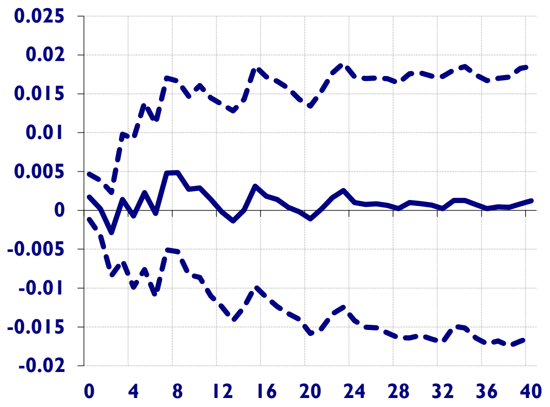

(f) The United Kingdom

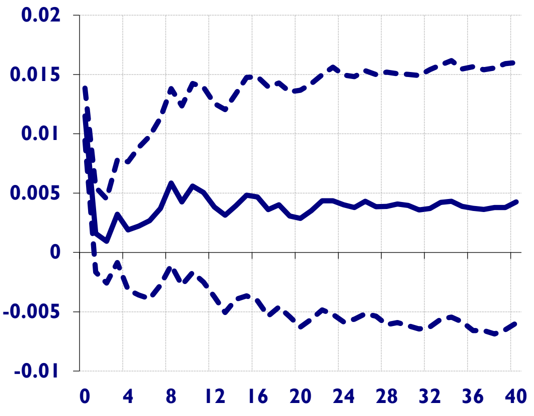

(g) The rest of the EU

Figure S23. Generalized impulse response functions (GIRFs) of intra-EU cheese export prices after a negative one-standard-error shock to the REU′ intra EU export price

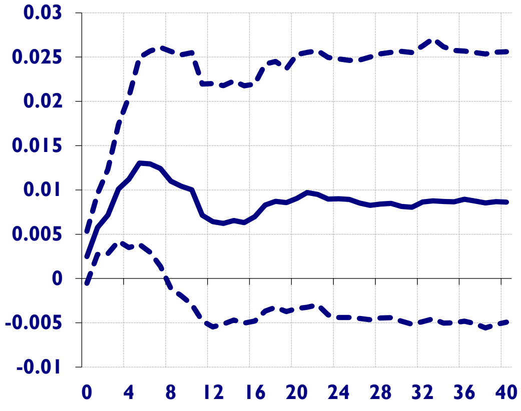

(a) Ireland

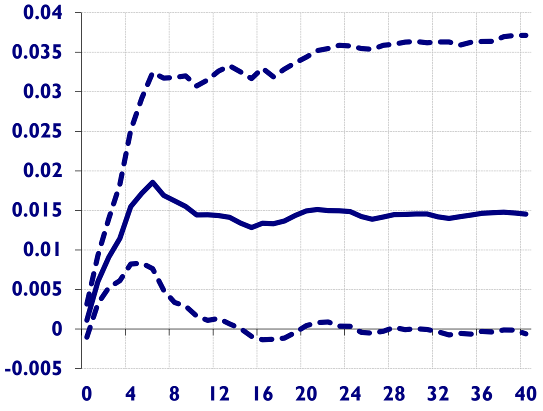

(b) The Netherlands

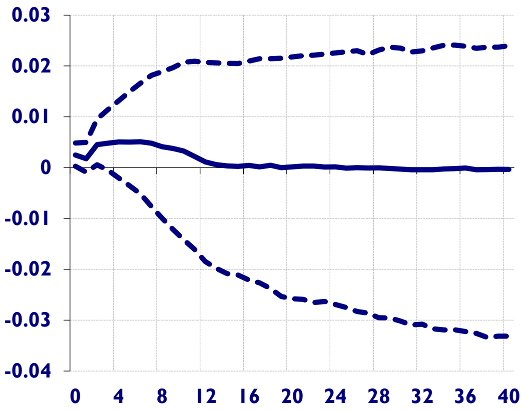

(c) Italy

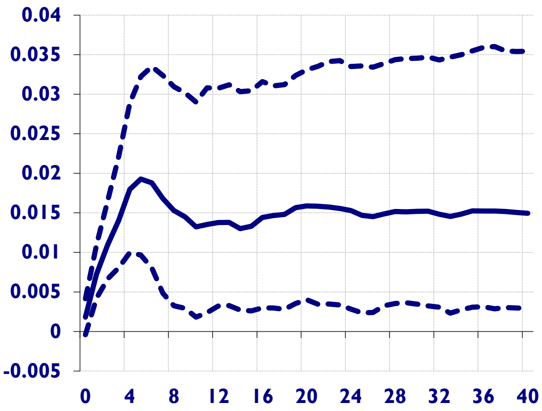

(d) Germany

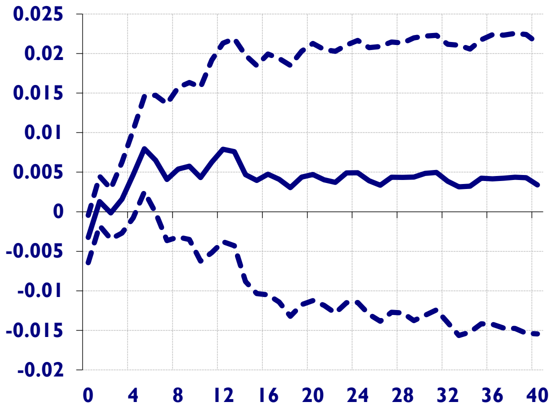

(e) France

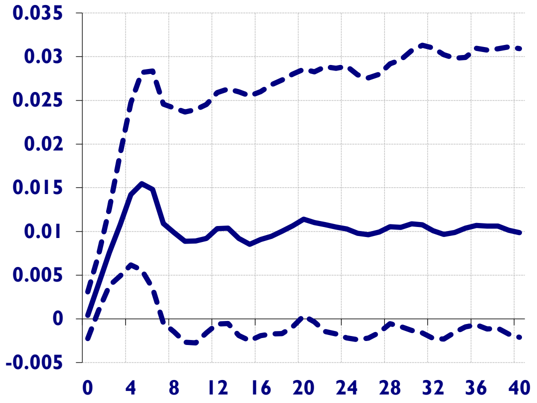

(f) The United Kingdom

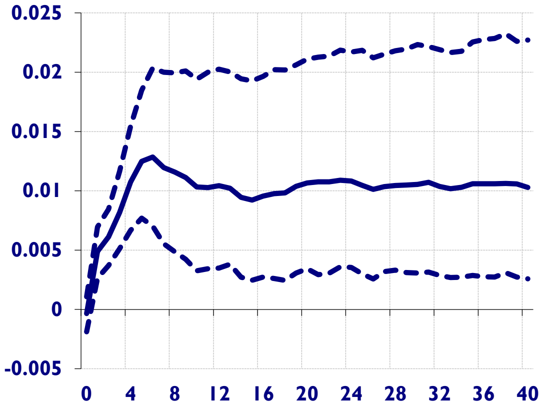

(g) The rest of the EU

Figure S24. Generalized impulse response functions (GIRFs) of intra-EU cheese export prices after a negative one-standard-error shock to the REU′ raw milk price

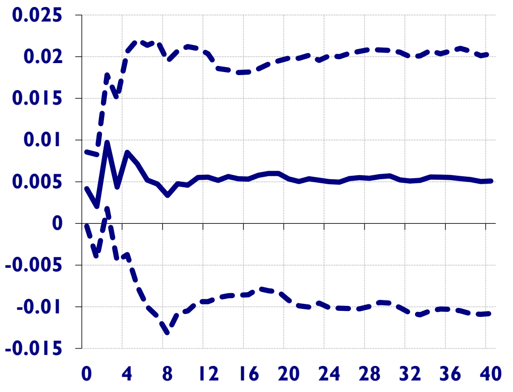

(a) Ireland

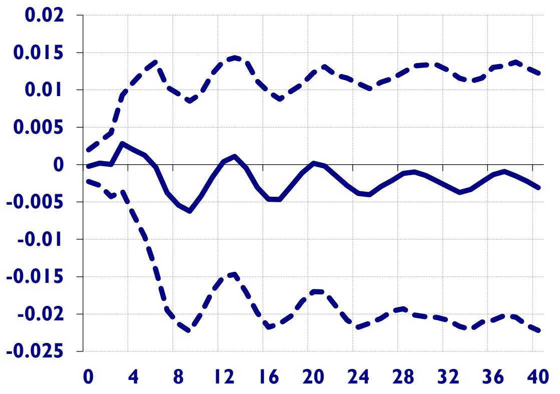

(b) The Netherlands

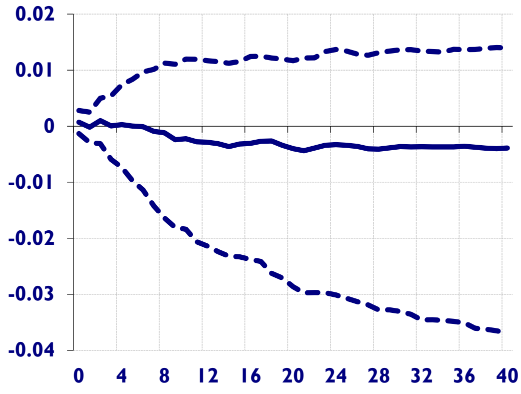

(c) Italy

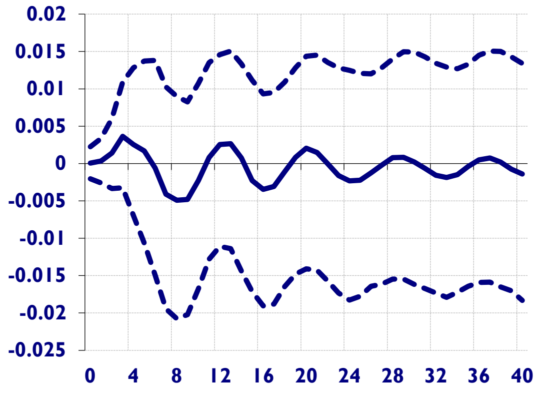

(d) Germany

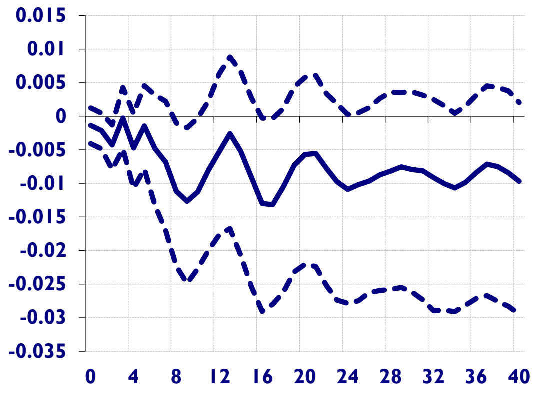

(e) France

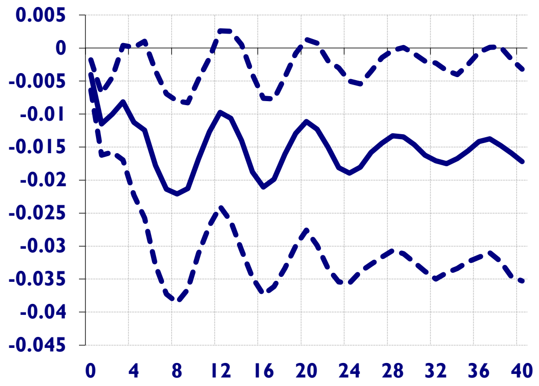

(f) The United Kingdom

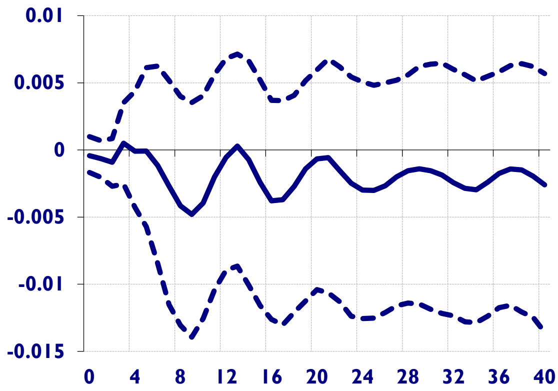

(g) The rest of the EU

Figure S25. Generalized impulse response functions (GIRFs) of intra-EU cheese export prices after a negative one-standard-error shock to the UK' cheese production

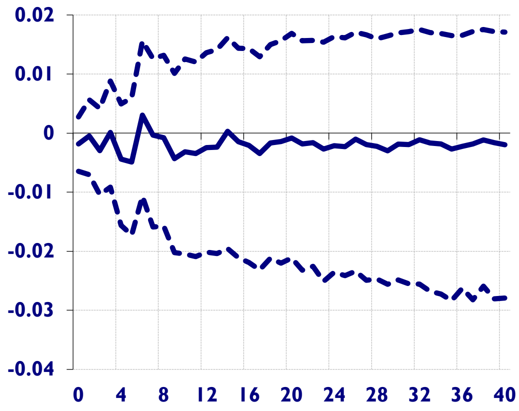

(a) Ireland

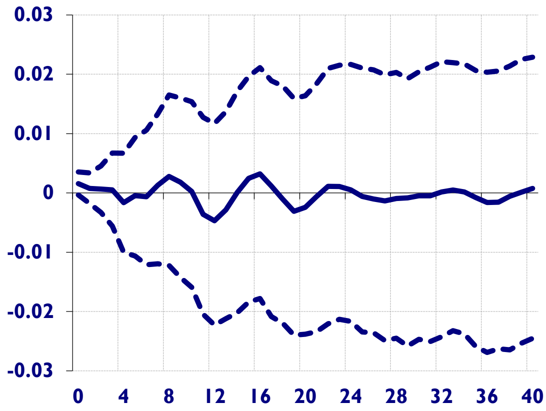

(b) The Netherlands

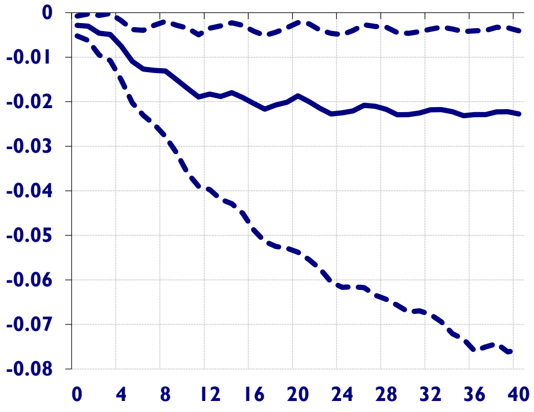

(c) Italy

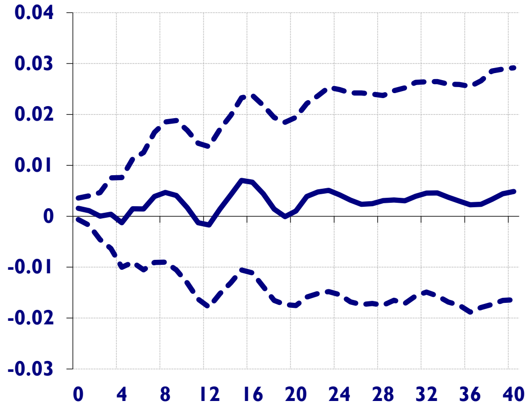

(d) Germany

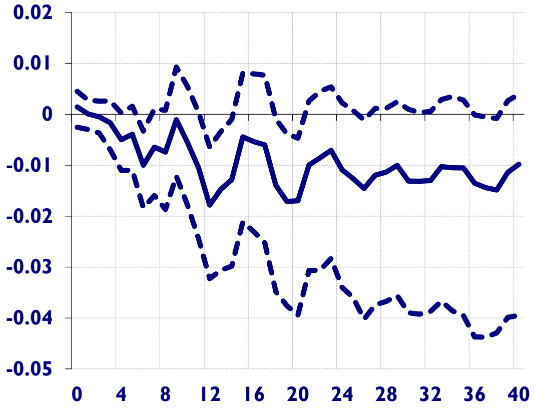

(e) France

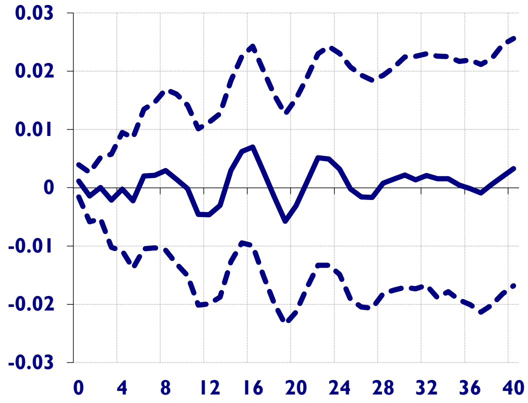

(f) The United Kingdom

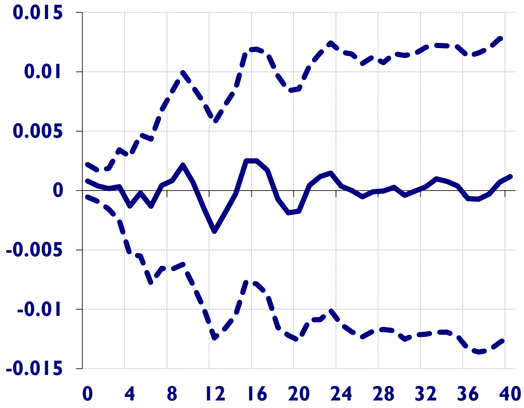

(g) The rest of the EU

Figure S26. Generalized impulse response functions (GIRFs) of intra-EU cheese export prices after a negative one-standard-error shock to the UK' HICP

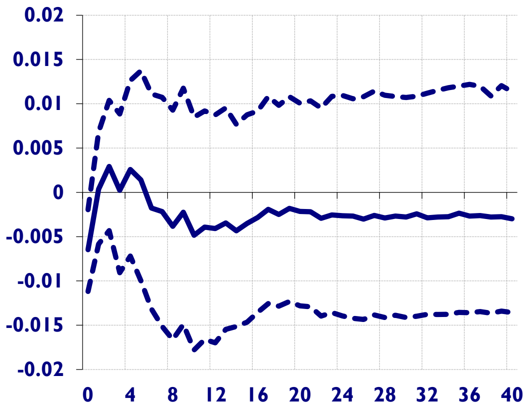

(a) Ireland

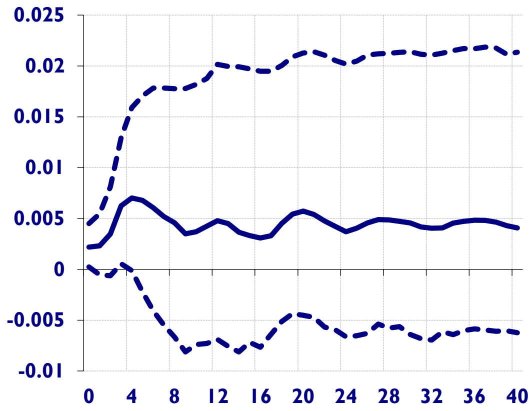

(b) The Netherlands

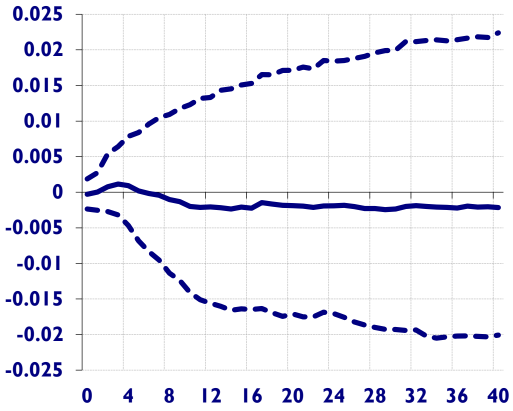

(c) Italy

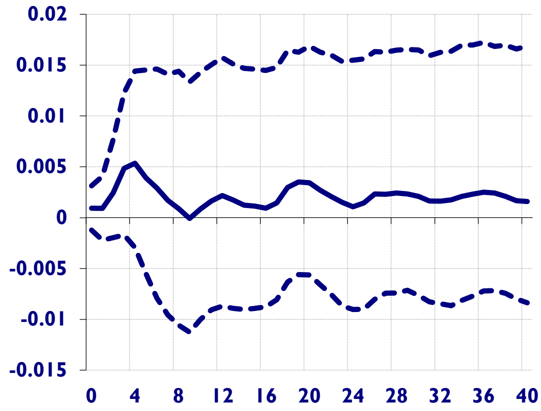

(d) Germany

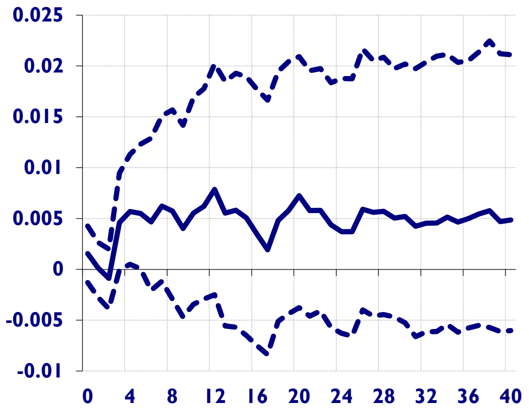

(e) France

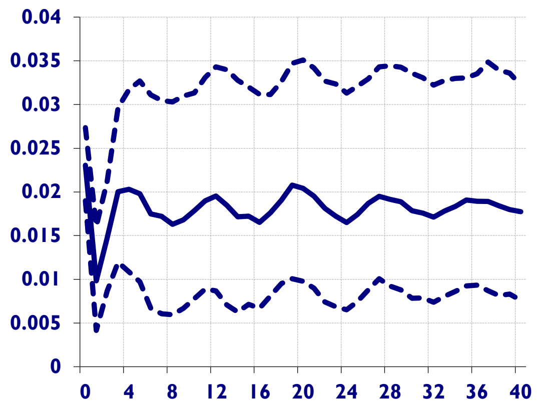

(f) The United Kingdom

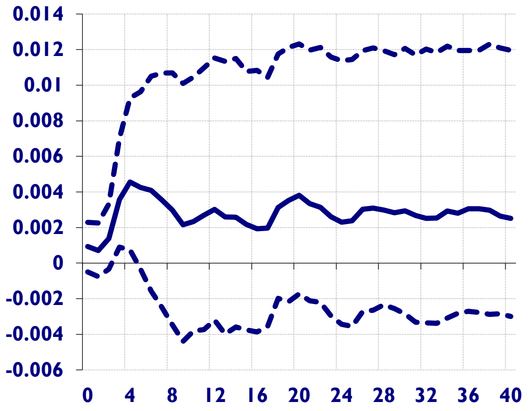

(g) The rest of the EU

Figure S27. Generalized impulse response functions (GIRFs) of intra-EU cheese export prices after a negative one-standard-error shock to the UK' intra EU cheese export price

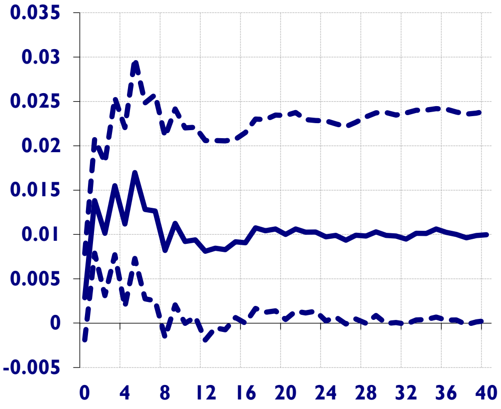

(a) Ireland

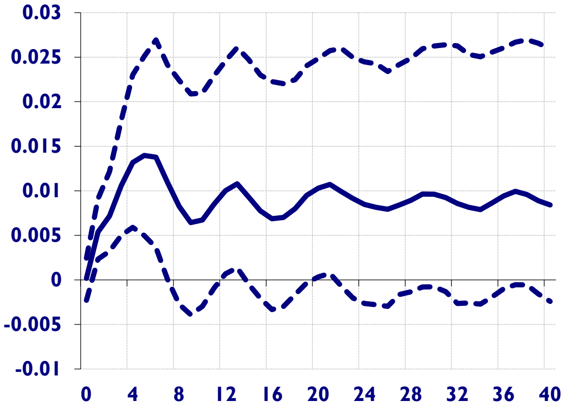

(b) The Netherlands

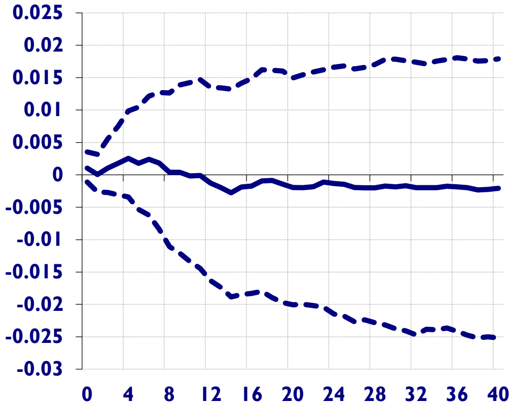

(c) Italy

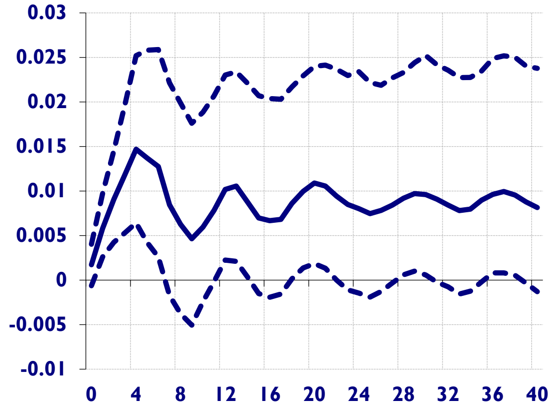

(d) Germany

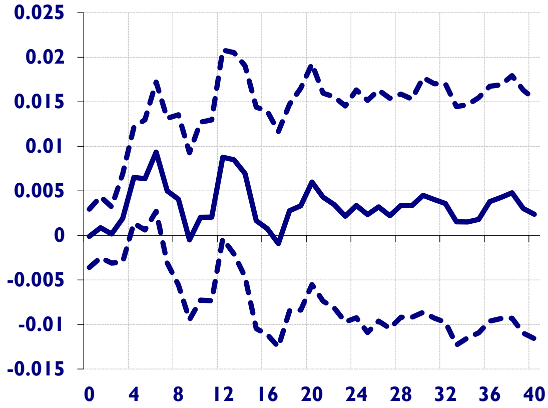

(e) France

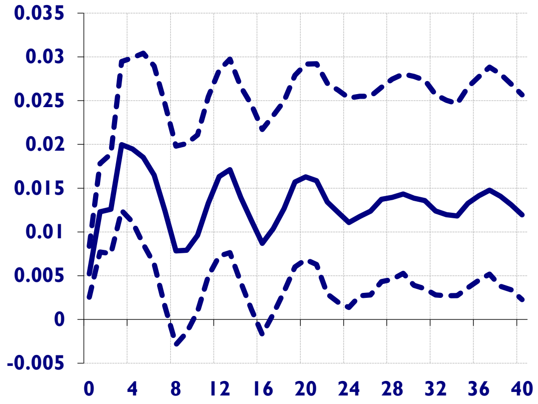

(f) The United Kingdom

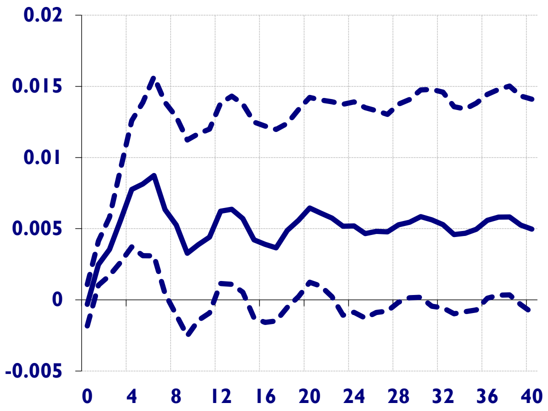

(g) The rest of the EU

Figure S28. Generalized impulse response functions (GIRFs) of intra-EU cheese export prices after a negative one-standard-error shock to the UK' raw milk price

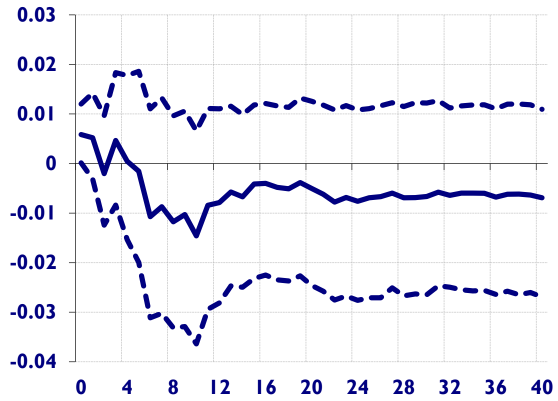

(a) Ireland

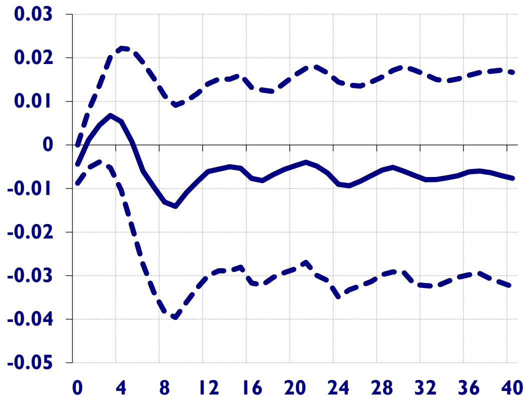

(b) The Netherlands

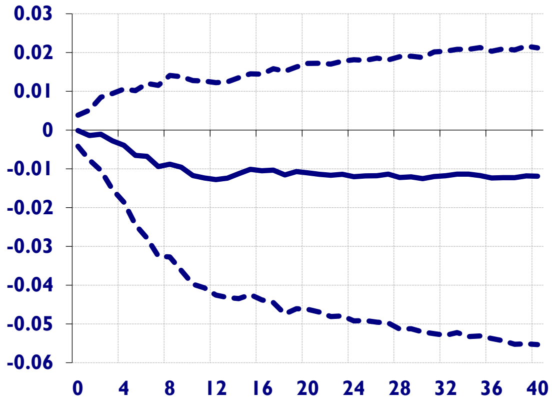

(c) Italy

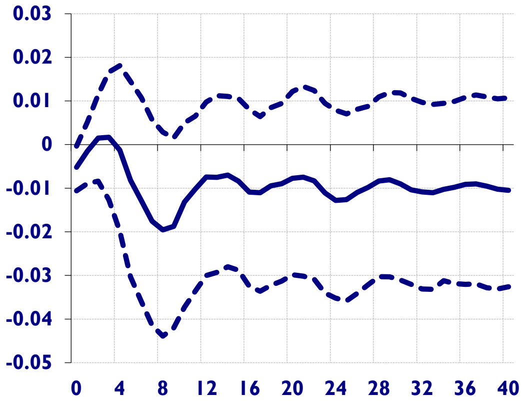

(d) Germany

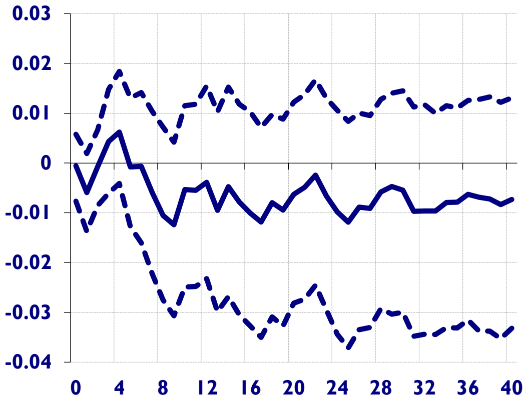

(e) France

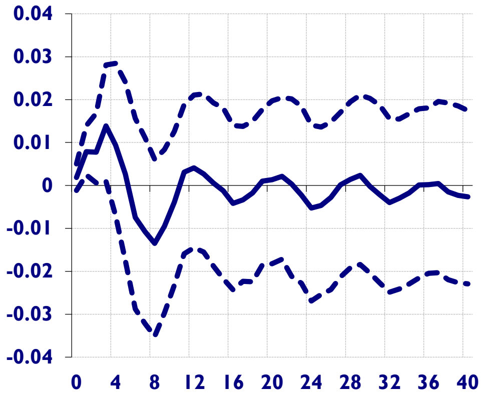

(f) The United Kingdom

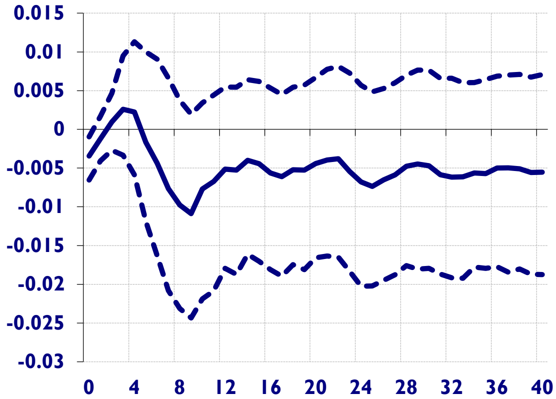

(g) The rest of the EU

Figure S29. Generalized impulse response functions (GIRFs) of intra-EU cheese export prices after a negative one-standard-error shock to crude oil price

The following figures depict the GIRFs of extra-EU cheese export prices after simulated shocks (bootstrap median estimates with 90% bootstrap error bounds).

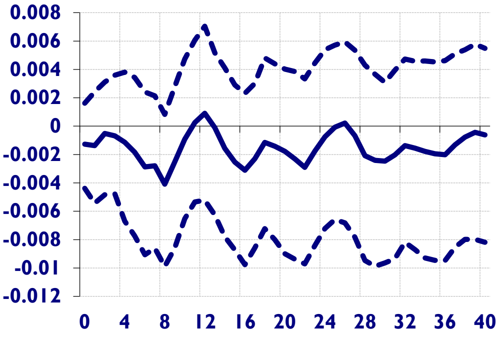

(a) Ireland

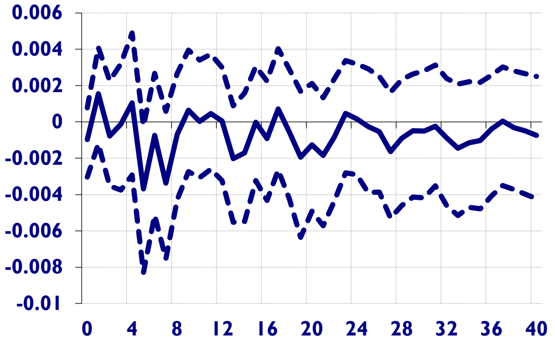

(b) The Netherlands

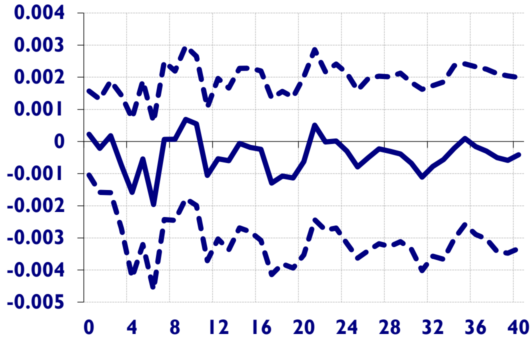

(c) Italy

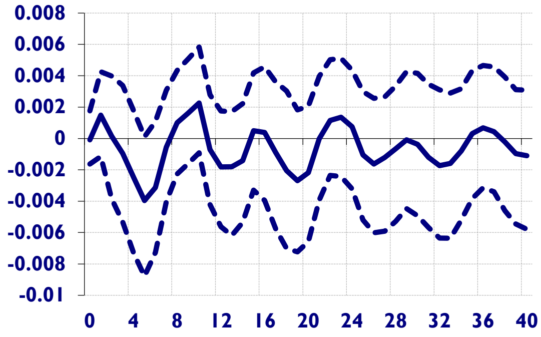

(d) Germany

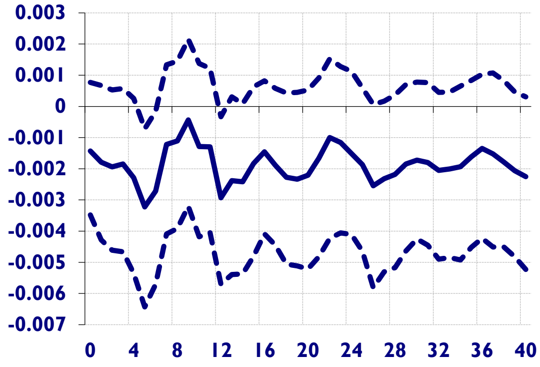

(e) France

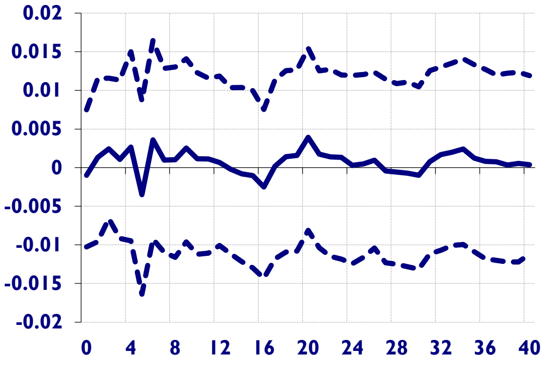

(f) The United Kingdom

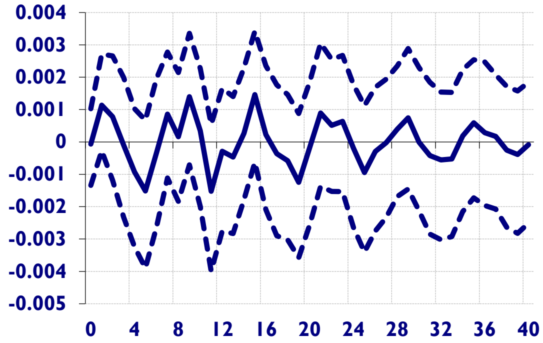

(g) France

Figure S30. Generalized impulse response functions (GIRFs) of extra-EU cheese export prices after a negative one-standard-error shock to France's cheese production

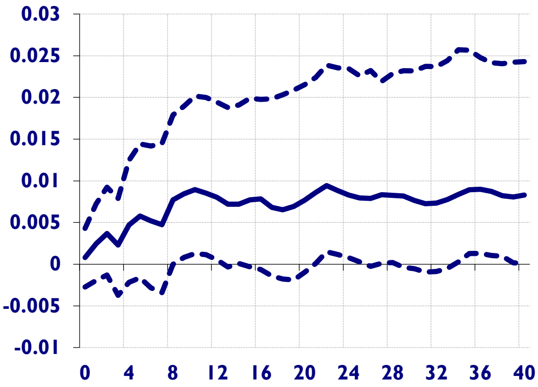

(a) Ireland

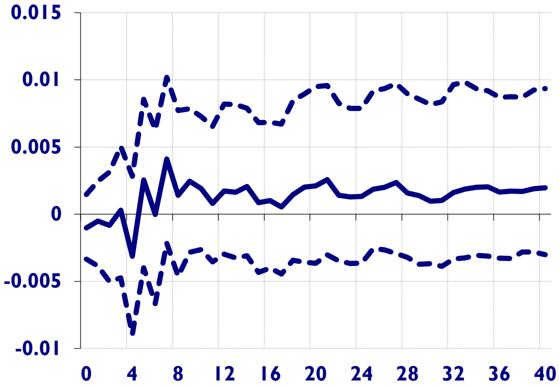

(b) The Netherlands

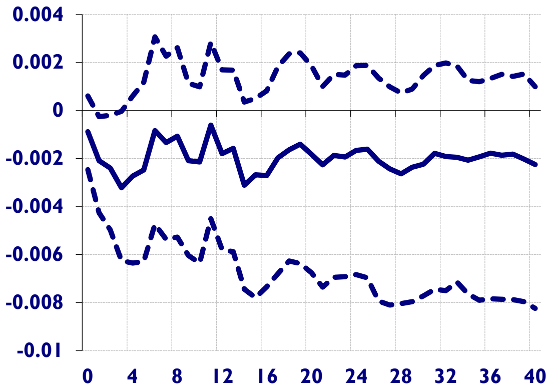

(c) Italy

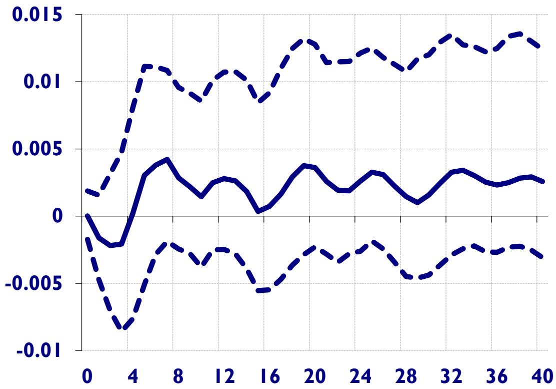

(d) Germany

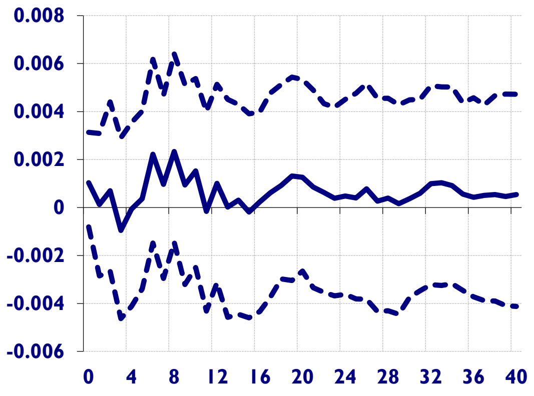

(e) France

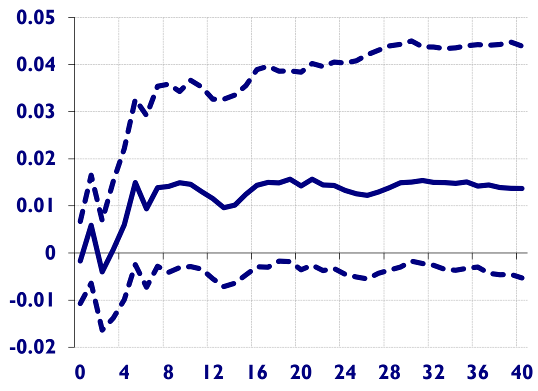

(f) The United Kingdom

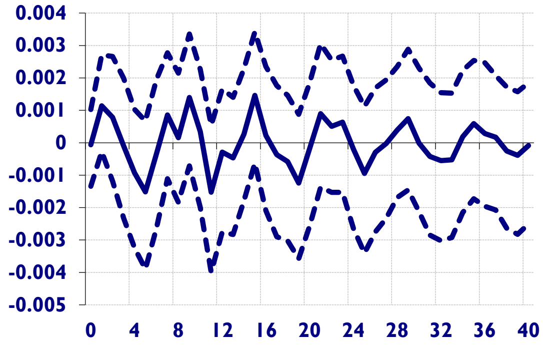

(g) The Rest of the EU

Figure S31. Generalized impulse response functions (GIRFs) of extra-EU cheese export prices after a positive one-standard-error shock to France's HICP

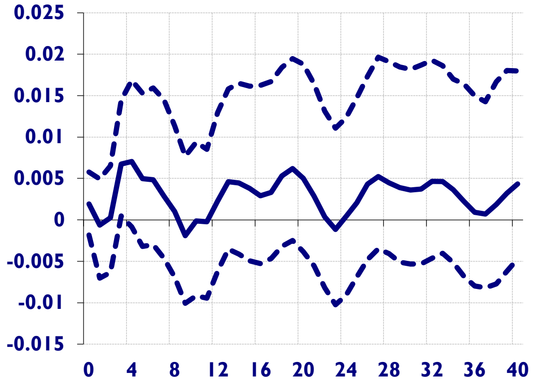

(a) Ireland

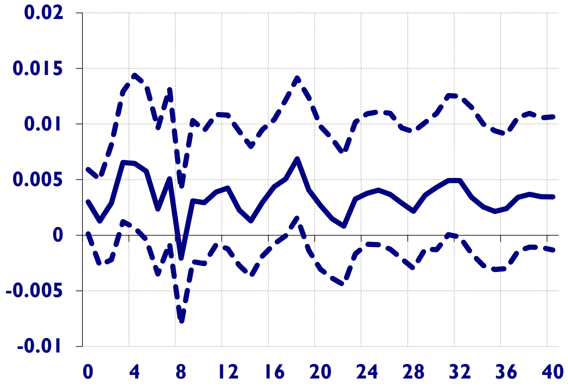

(b) The Netherlands

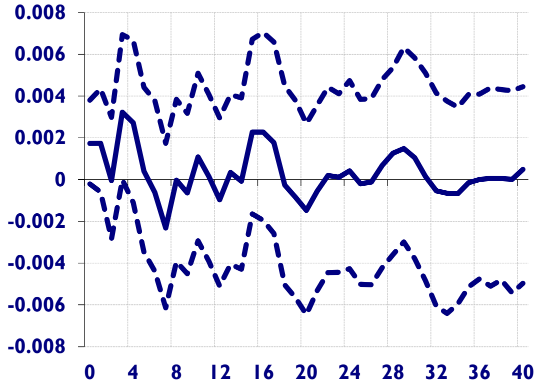

(c) Italy

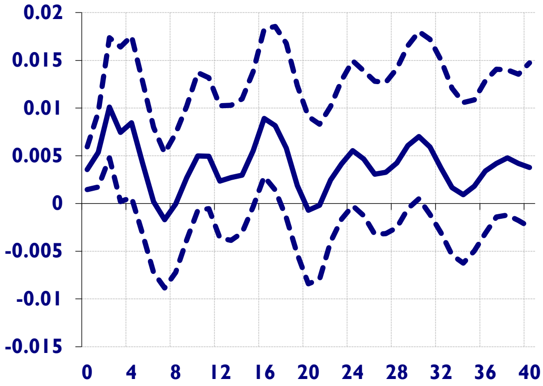

(d) Germany

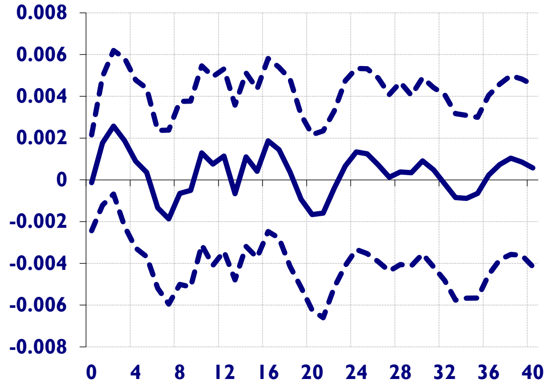

(e) France

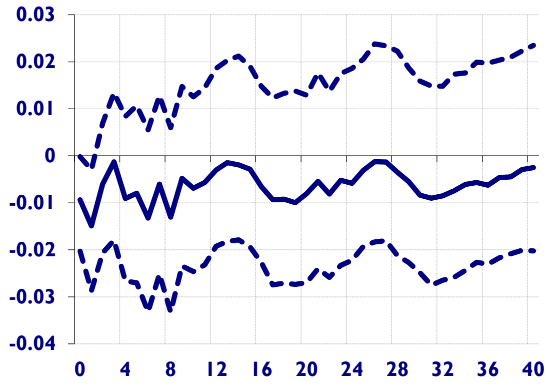

(f) The United Kingdom

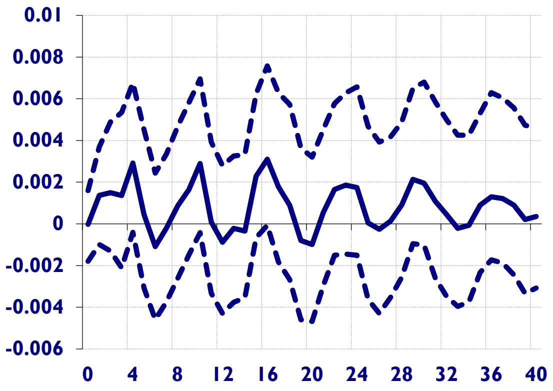

(g) The Rest of the EU

Figure S32. Generalized impulse response functions (GIRFs) of extra-EU cheese export prices after a positive one-standard-error shock to France's raw milk price

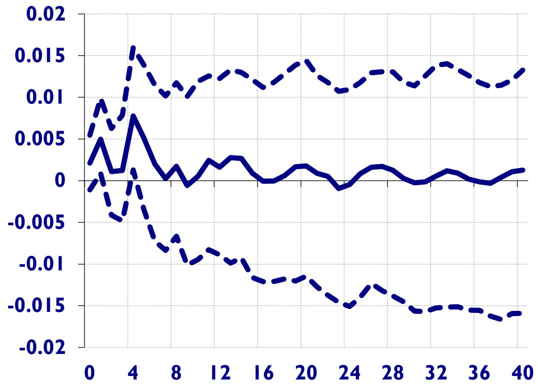

(a) Ireland

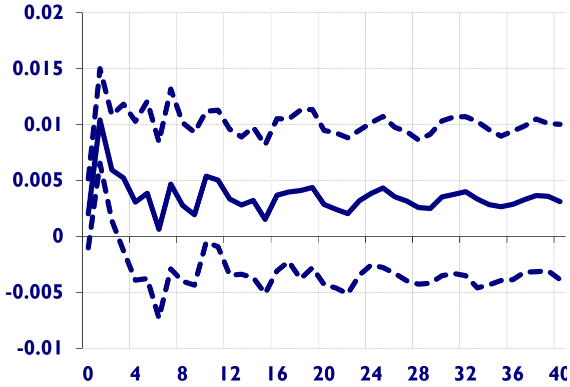

(b) The Netherlands

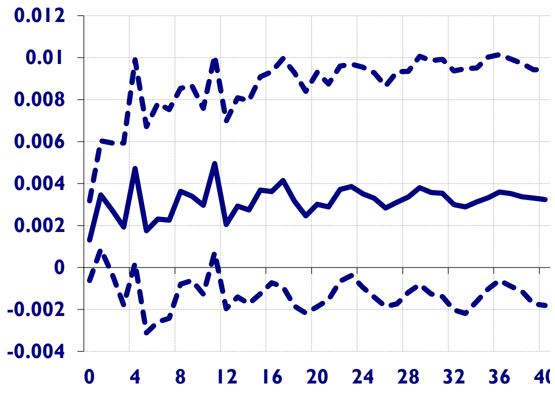

(c) Italy

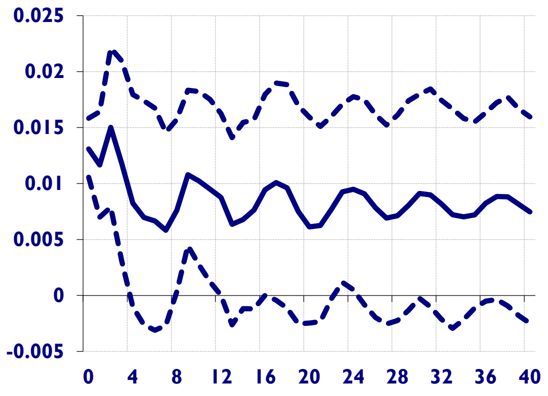

(d) Germany

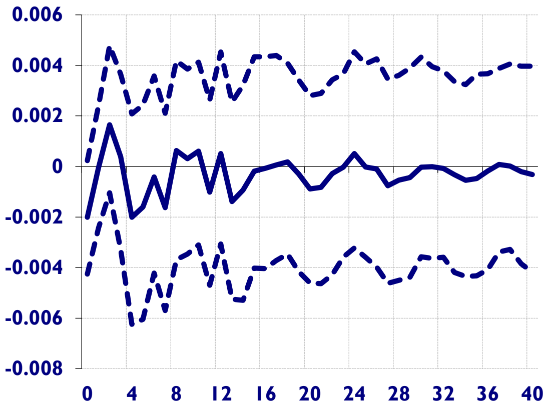

(e) France

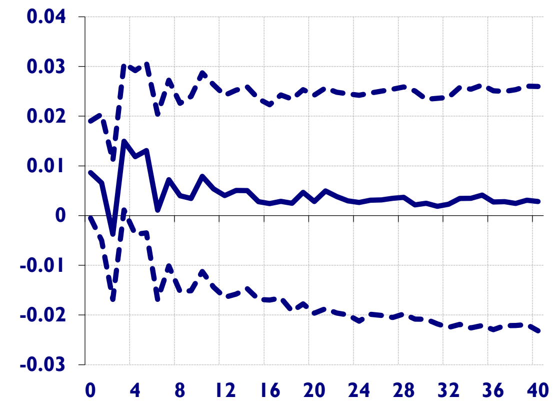

(f) The United Kingdom

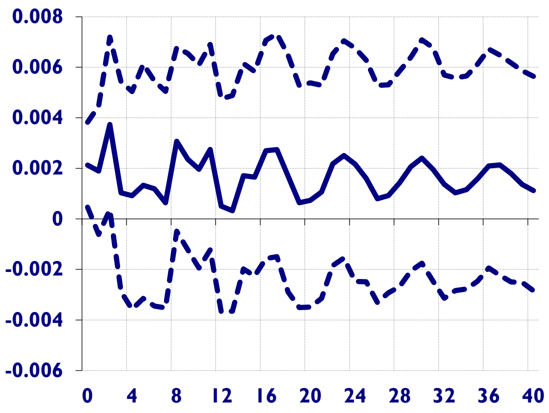

(g) The Rest of the EU

Figure S33. Generalized impulse response functions (GIRFs) of extra-EU cheese export prices after a positive one-standard-error shock to Germany's extra-EU export price

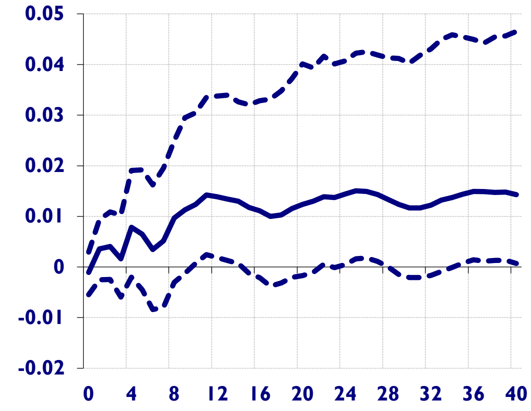

(a) Ireland

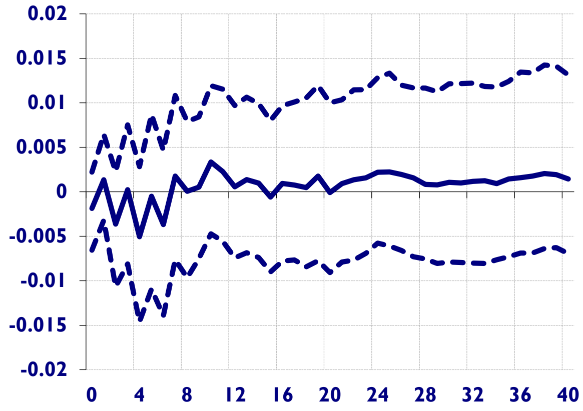

(b) The Netherlands

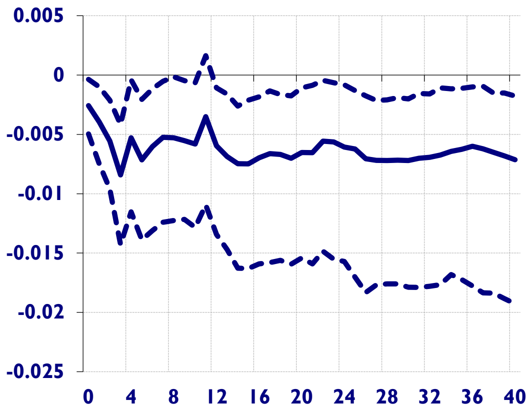

(c) Italy

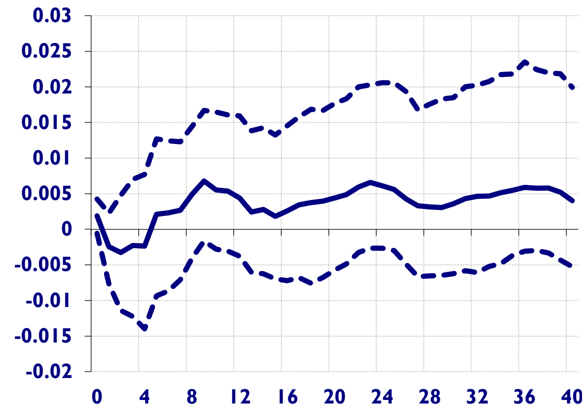

(d) Germany

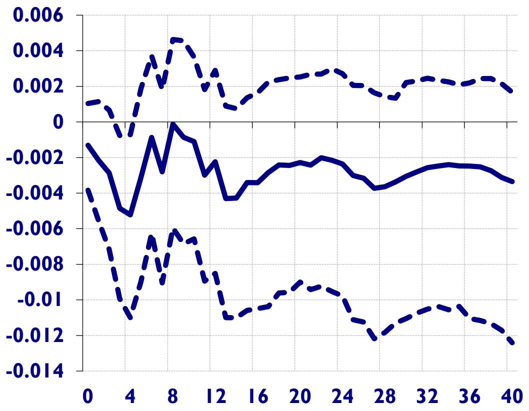

(e) France

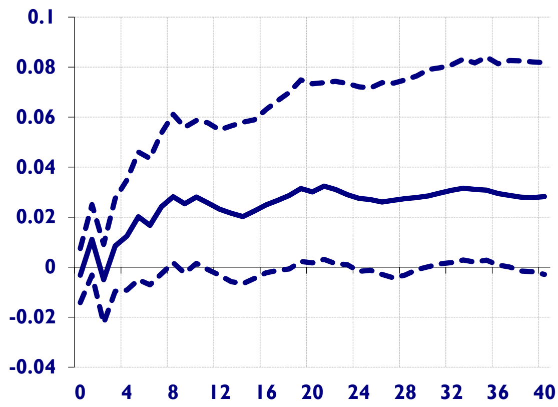

(f) The United Kingdom

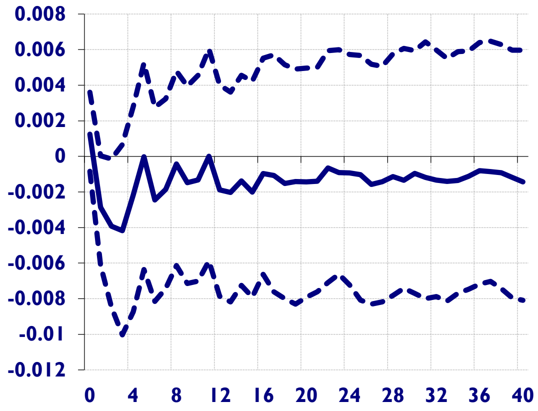

(g) The Rest of the EU

Figure S34. Generalized impulse response functions (GIRFs) of extra-EU cheese export prices after a positive one-standard-error shock to Germany's HICP

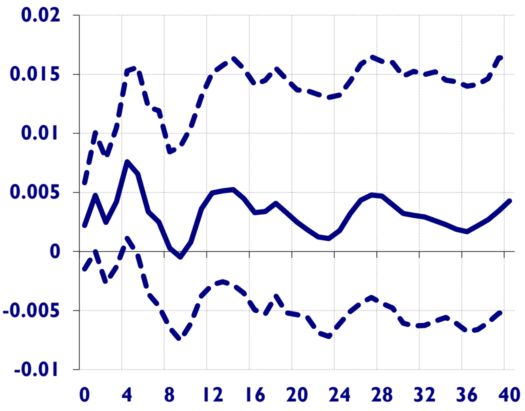

(a) Ireland

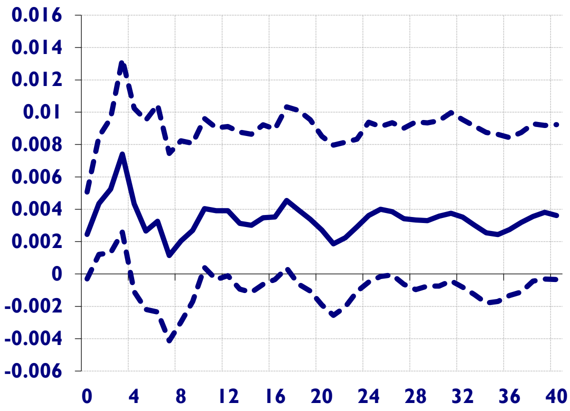

(b) The Netherlands

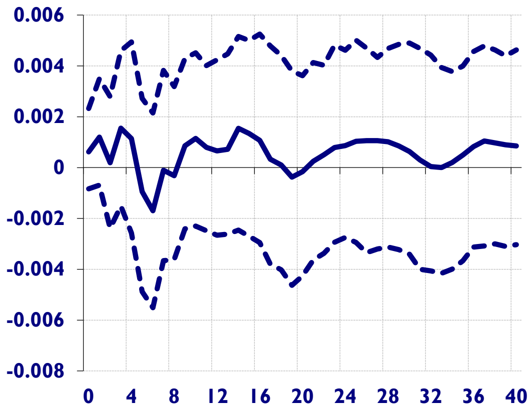

(c) Italy

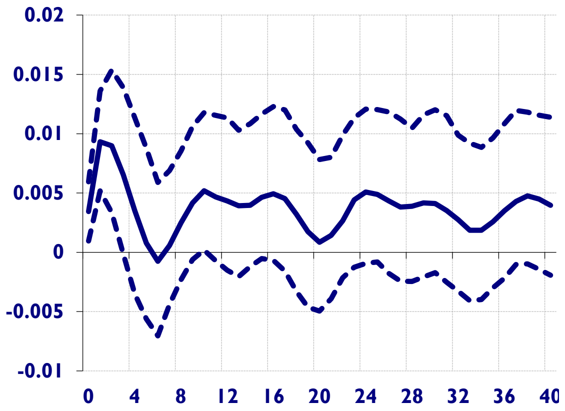

(d) Germany

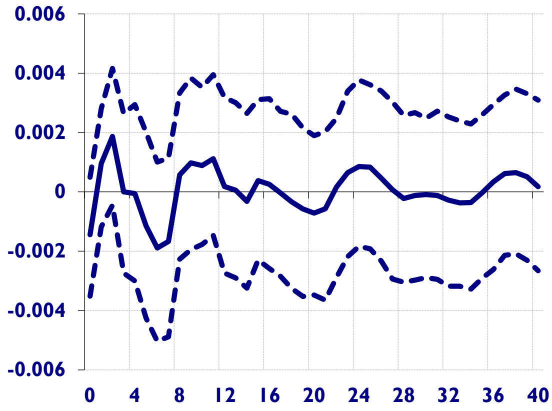

(e) France

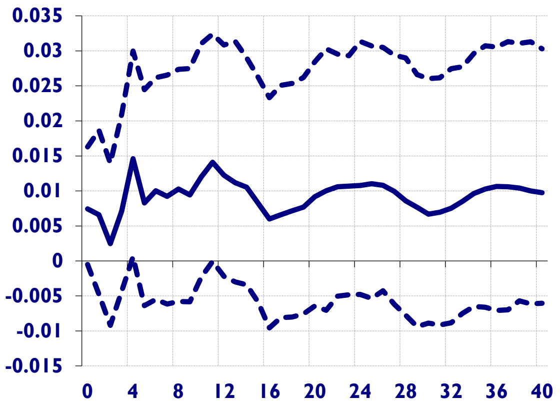

(f) The United Kingdom

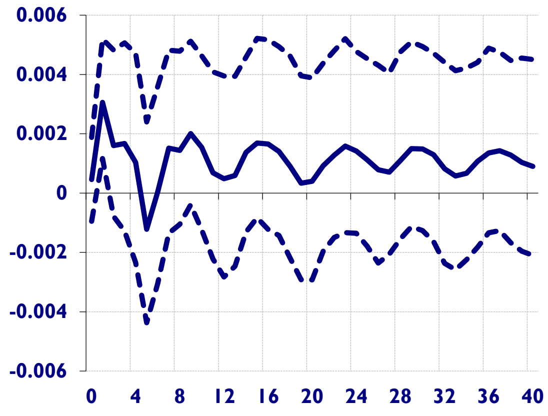

(g) The Rest of the EU

Figure S35. Generalized impulse response functions (GIRFs) of extra-EU cheese export prices after a positive one-standard-error shock to Germany's raw milk price

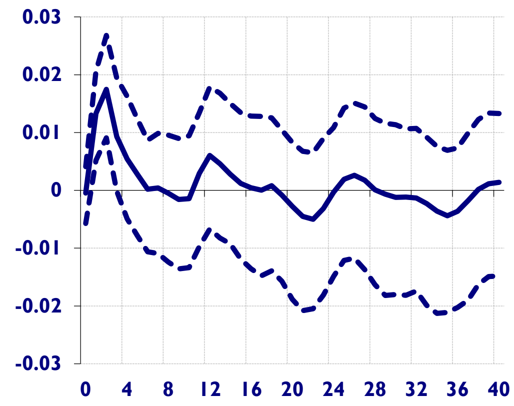

(a) Ireland

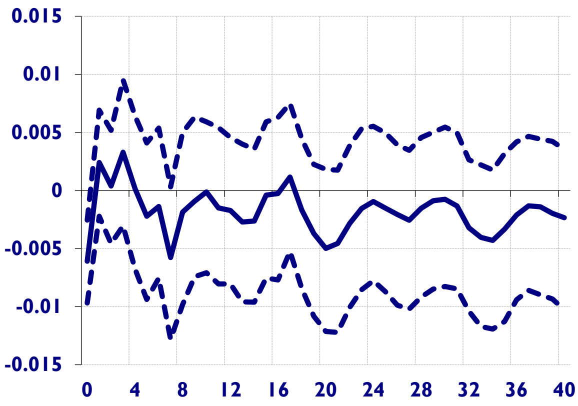

(b) The Netherlands

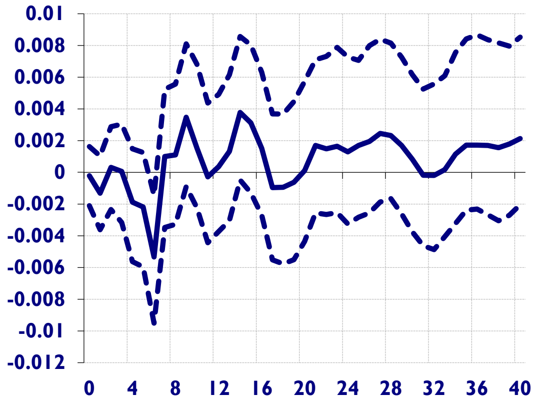

(c) Italy

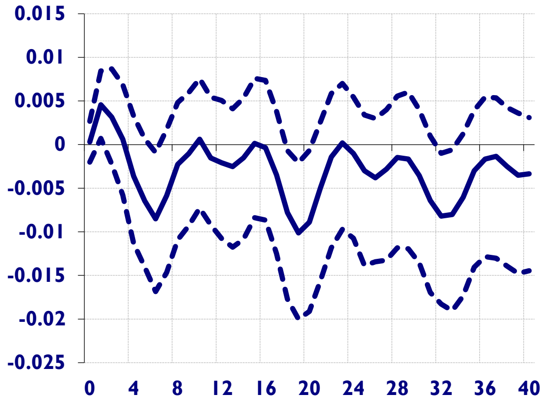

(d) Germany

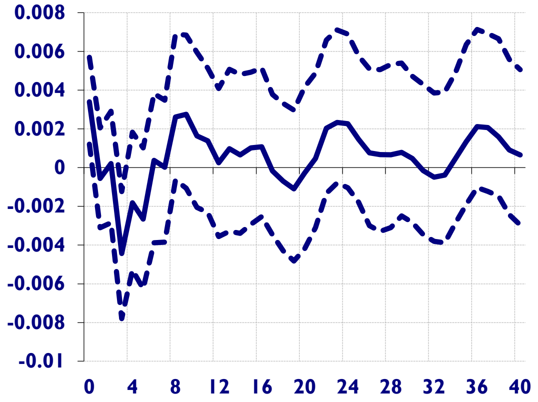

(e) France

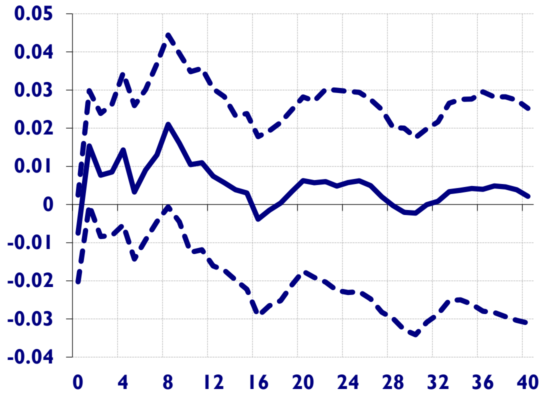

(f) The United Kingdom

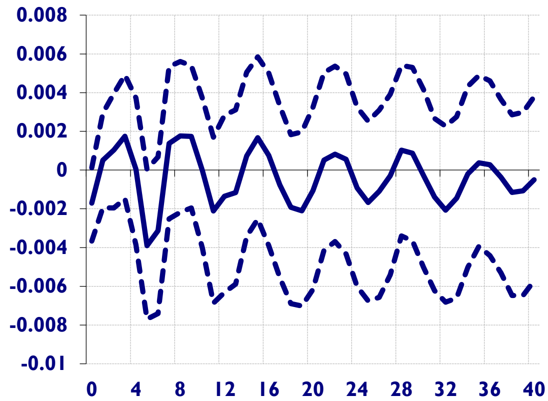

(g) The Rest of the EU

Figure S36. Generalized impulse response functions (GIRFs) of extra-EU cheese export prices after a negative one-standard-error shock to Ireland's cheese production

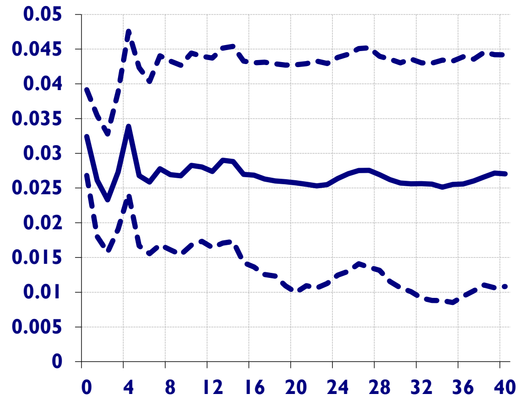

(a) Ireland

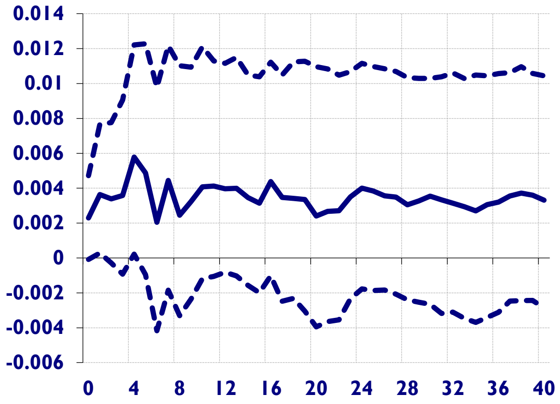

(b) The Netherlands

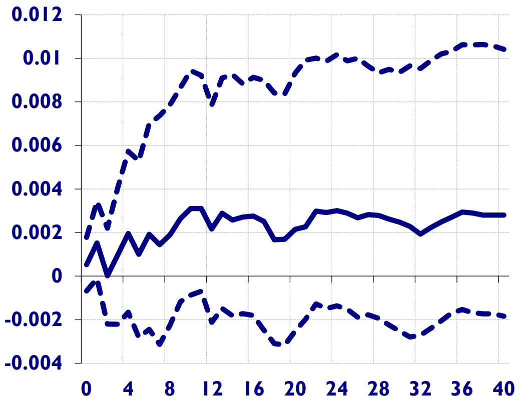

(c) Italy

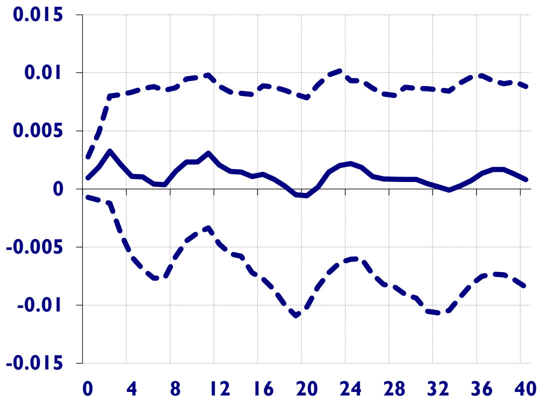

(d) Germany

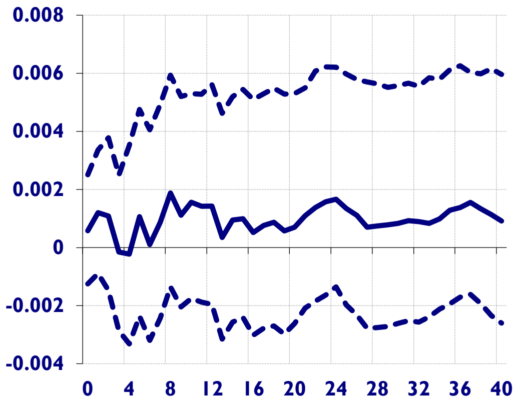

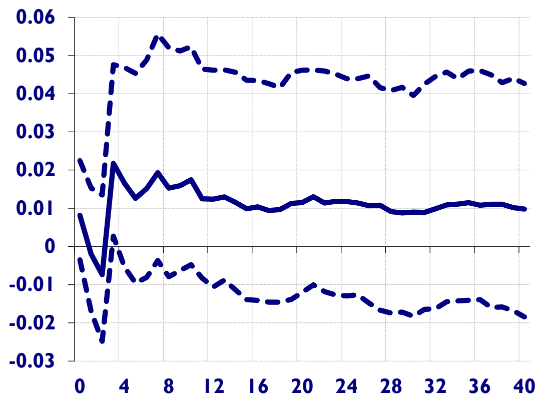

(e) France **(f)** The United Kingdom

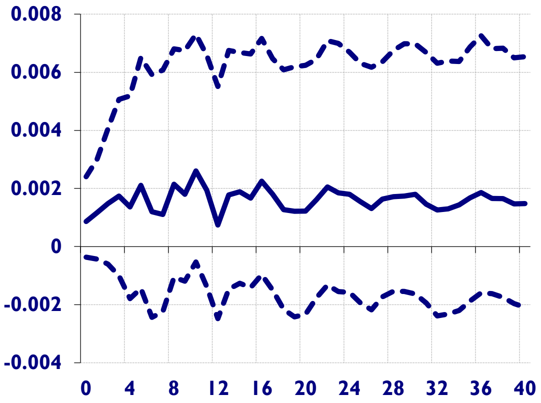

(g) The Rest of the EU

Figure S37. Generalized impulse response functions (GIRFs) of extra-EU cheese export prices after a positive one-standard-error shock to Ireland's extra EU cheese export price

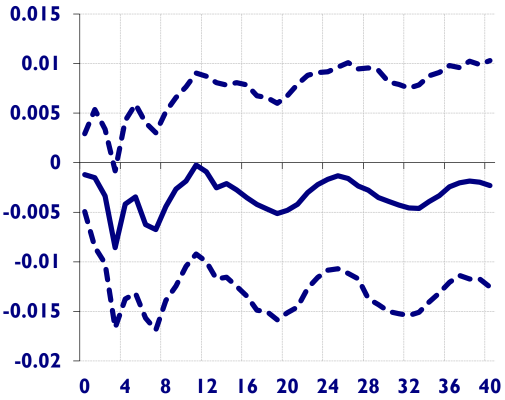

(a) Ireland

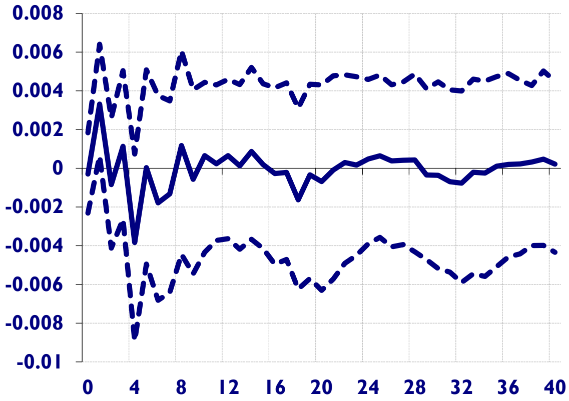

(b) The Netherlands

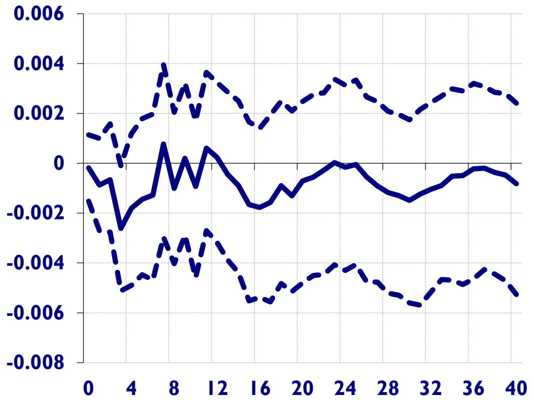

(c) Italy

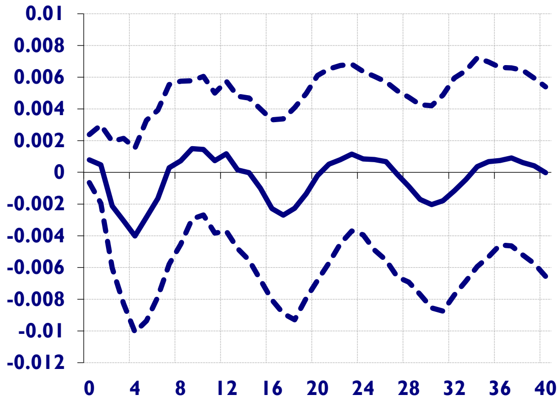

(d) Germany

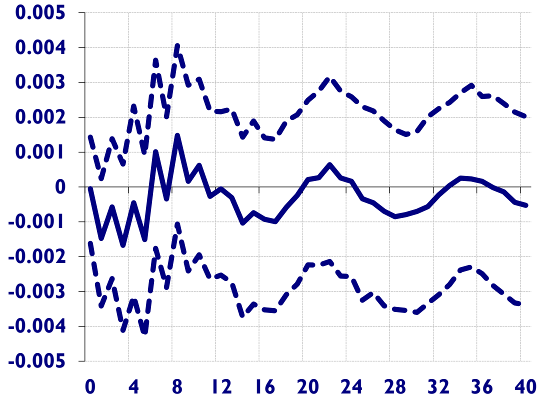

(e) France

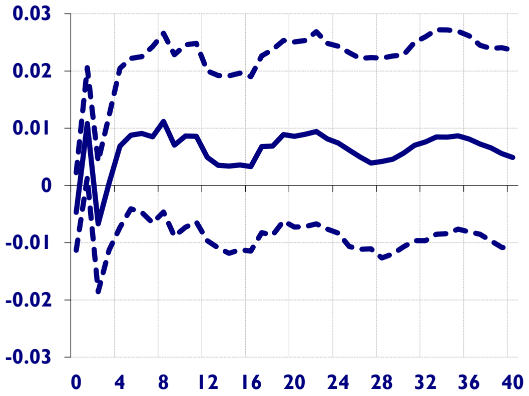

(f) The United Kingdom

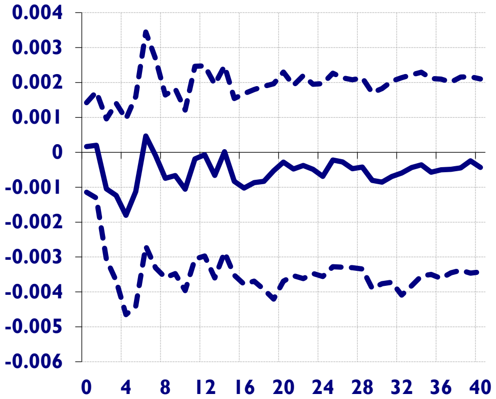

(g) The Rest of the EU

Figure S38. Generalized impulse response functions (GIRFs) of extra-EU cheese export prices after a positive one-standard-error shock to Ireland's HICP

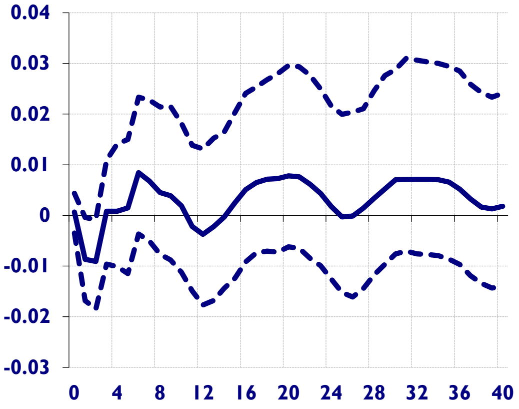

(a) Ireland

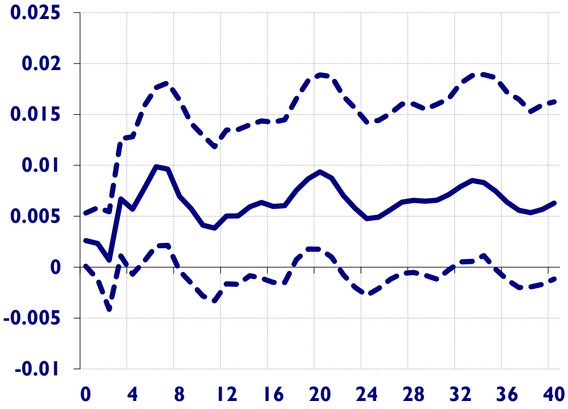

(b) The Netherlands

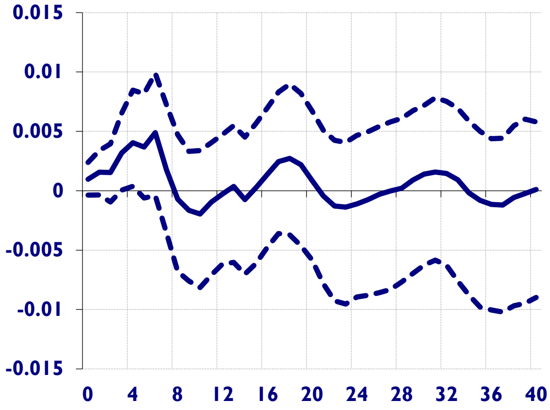

(c) Italy

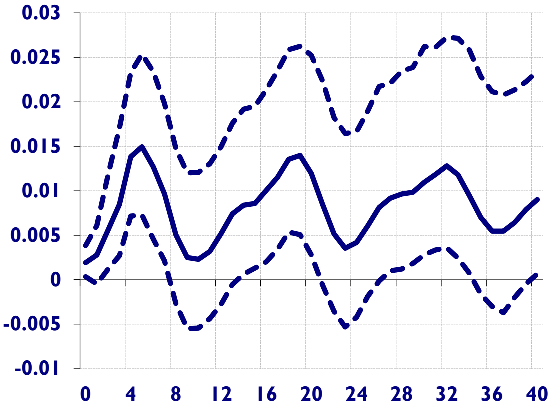

(d) Germany

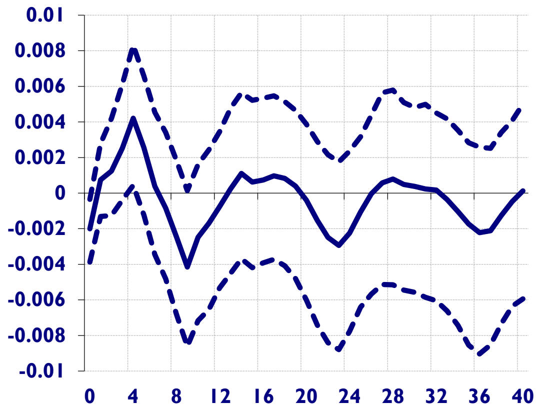

(e) France

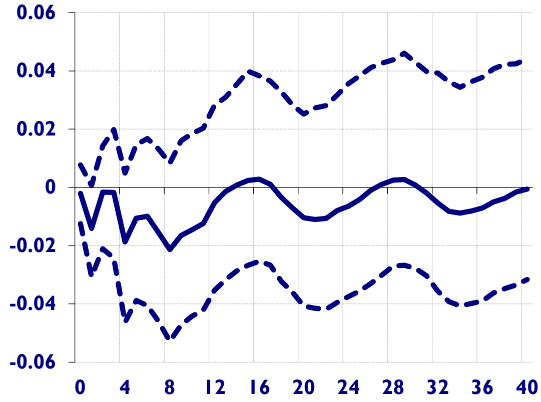

(f) The United Kingdom

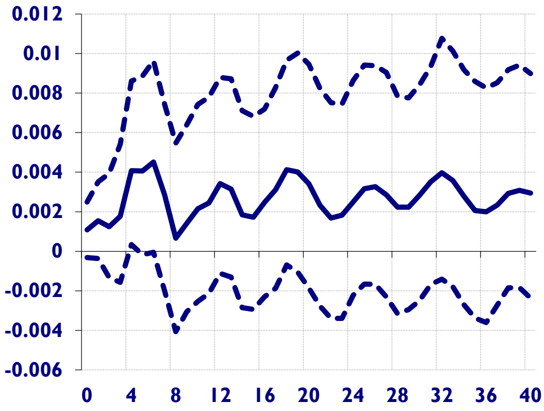

(g) The Rest of the EU

Figure S39. Generalized impulse response functions (GIRFs) of extra-EU cheese export prices after a positive one-standard-error shock to Ireland's raw milk price

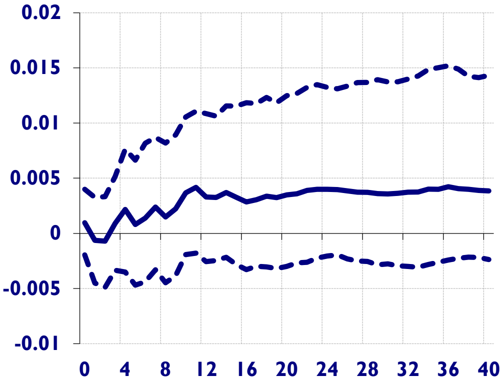

(a) Ireland

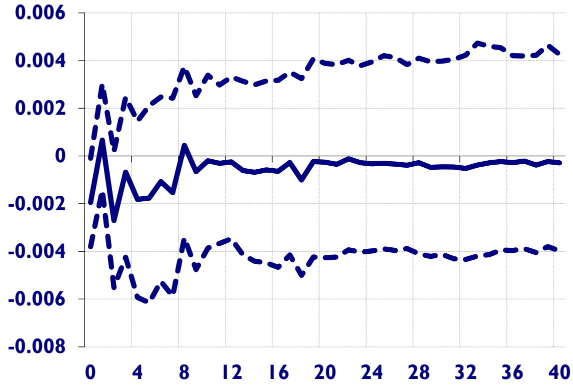

(b) The Netherlands

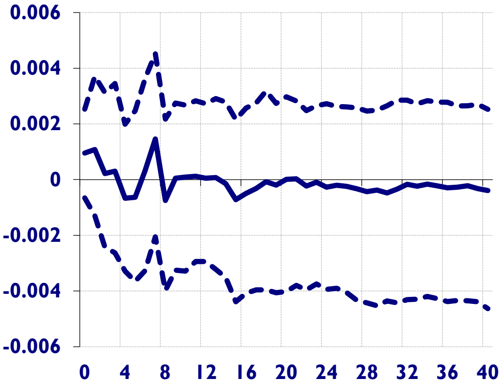

(c) Italy

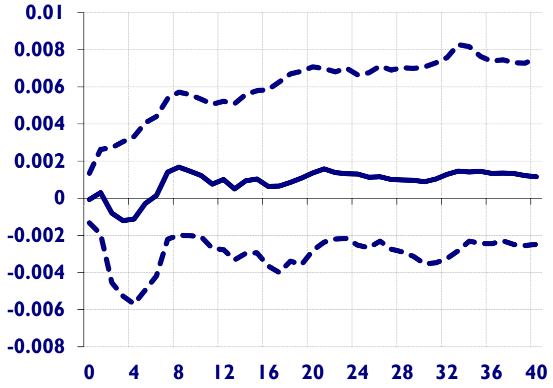

(d) Germany

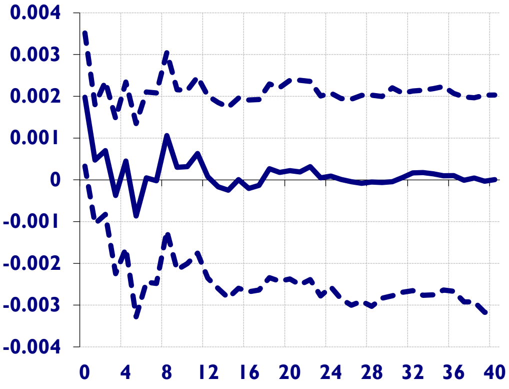

(e) France

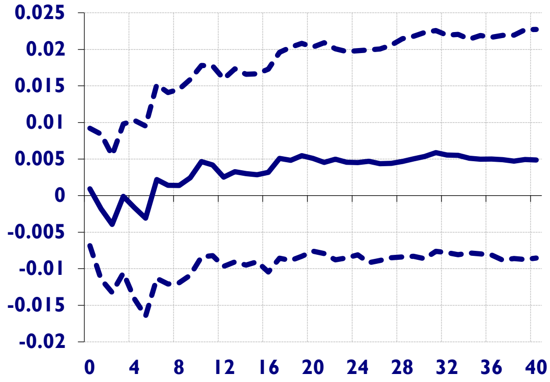

(f) The United Kingdom

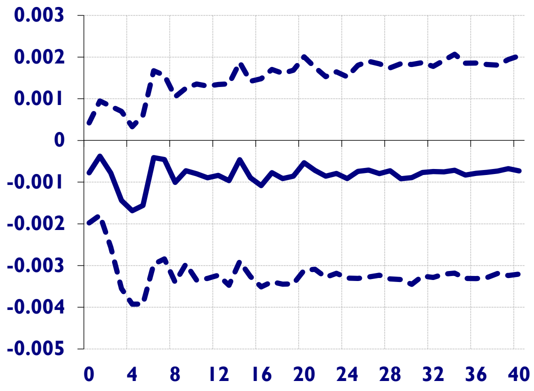

(g) The Rest of the EU

Figure S40. Generalized impulse response functions (GIRFs) of extra-EU cheese export prices after a negative one-standard-error shock to Italy's cheese production

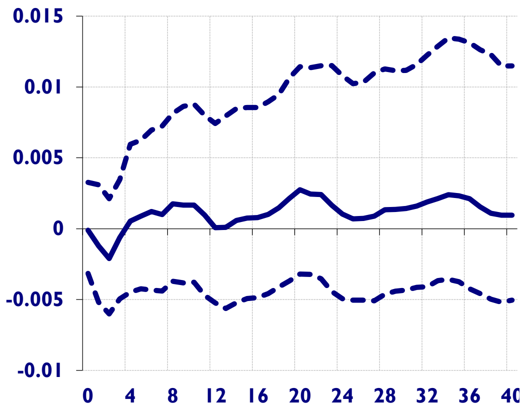

(a) Ireland

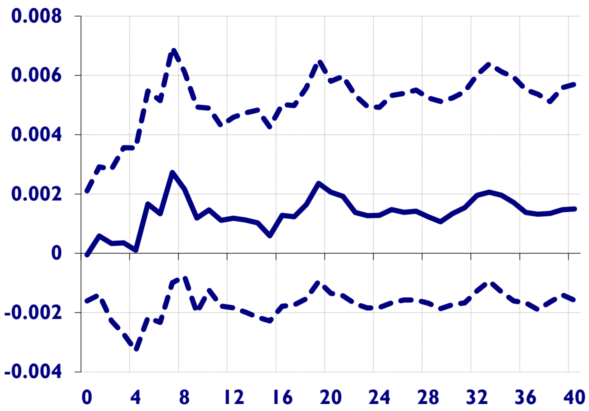

(b) The Netherlands

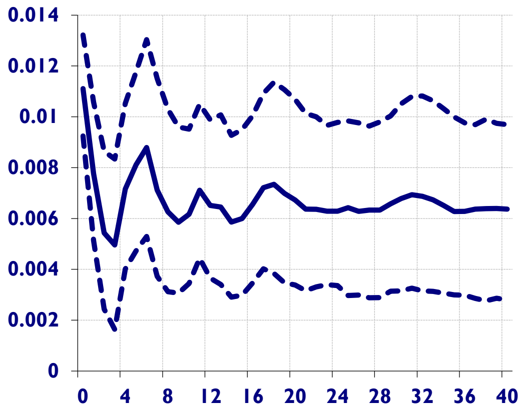

(c) Italy

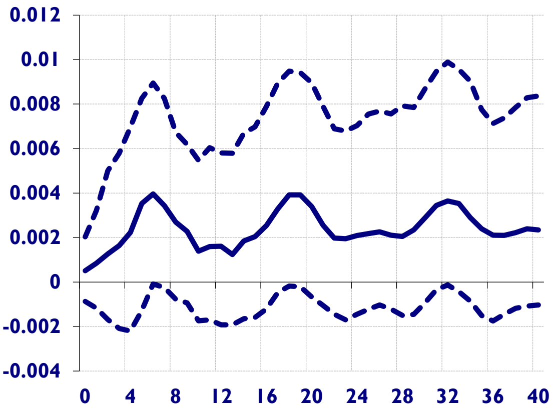

(d) Germany

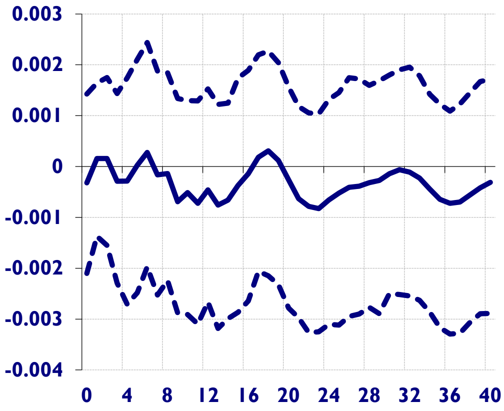

(e) France

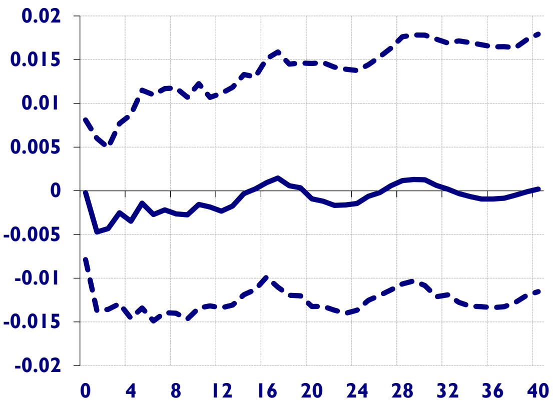

(f) The United Kingdom

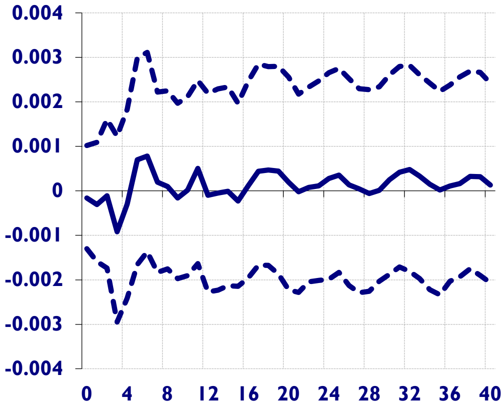

(g) The Rest of the EU

Figure S41. Generalized impulse response functions (GIRFs) of extra-EU cheese export prices after a positive one-standard-error shock to Italy's extra EU cheese export price

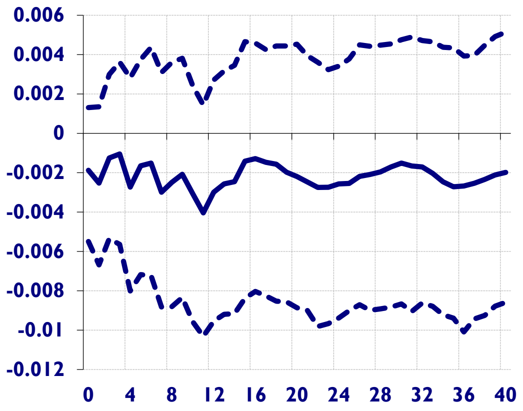

(a) Ireland

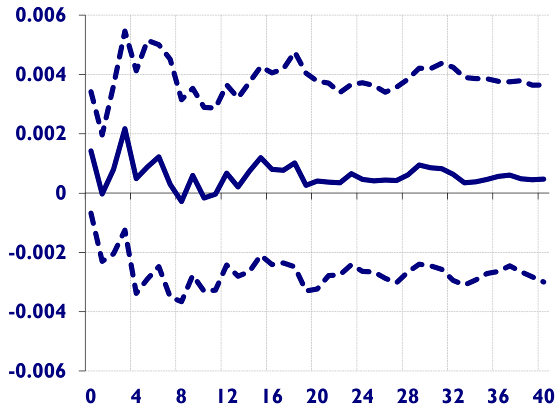

(b) The Netherlands

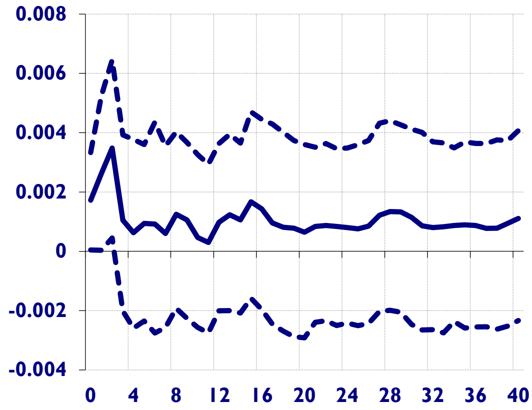

(c) Italy

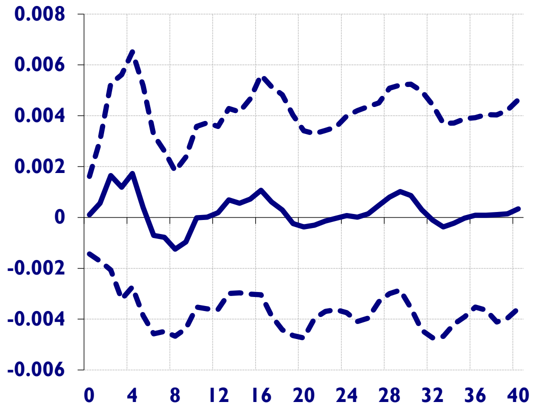

(d) Germany

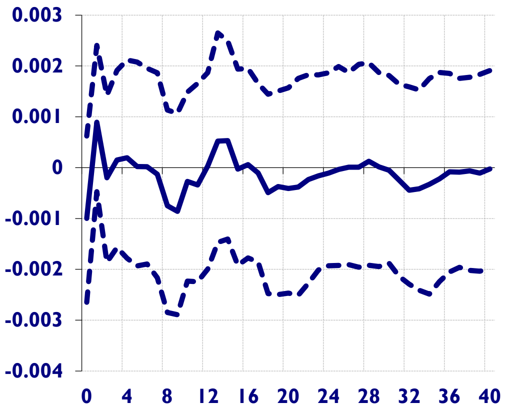

(e) France

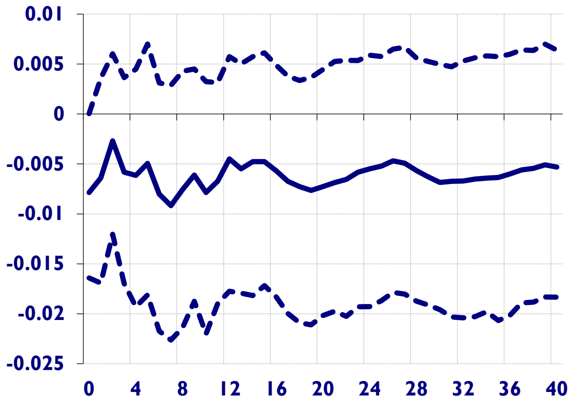

(f) The United Kingdom

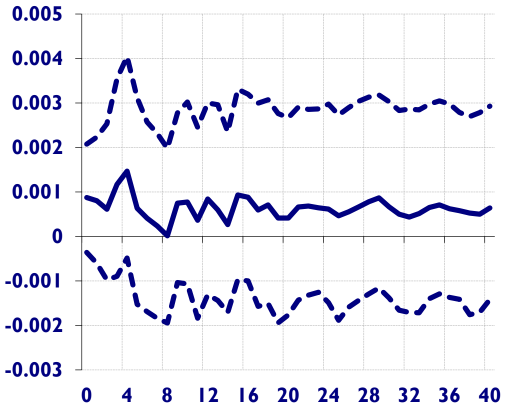

(g) The Rest of the EU

Figure S42. Generalized impulse response functions (GIRFs) of extra-EU cheese export prices after a positive one-standard-error shock to Italy's raw milk price

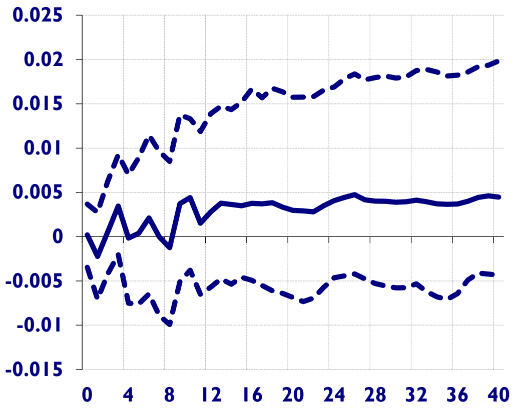

(a) Ireland

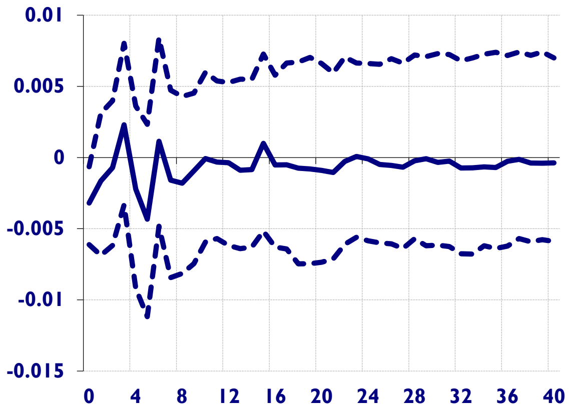

(b) The Netherlands

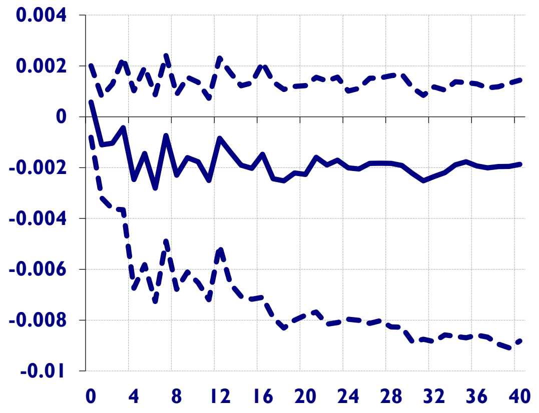

(c) Italy

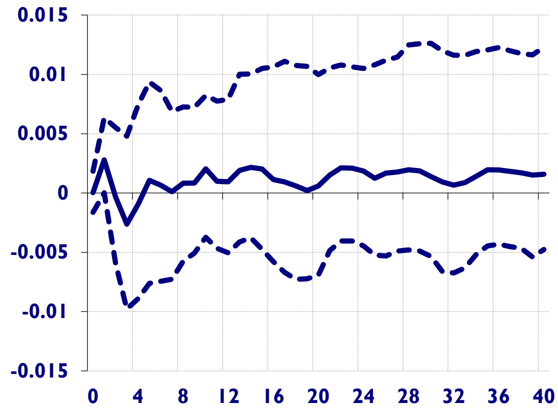

(d) Germany

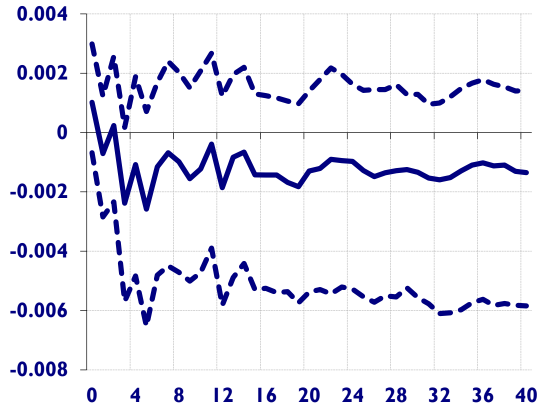

(e) France

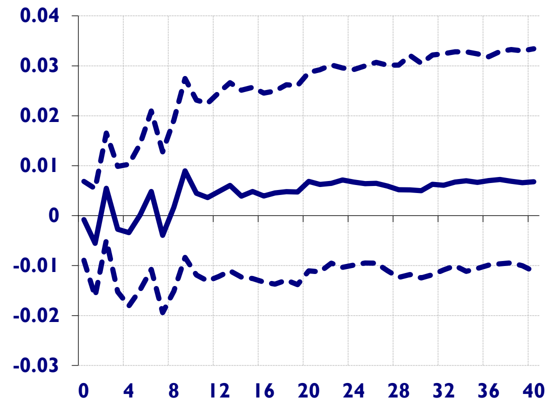

(f) The United Kingdom

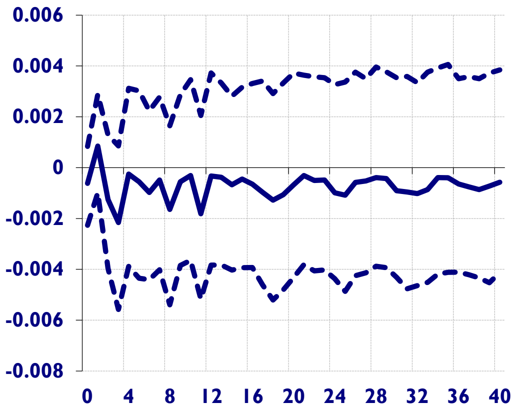

(g) The Rest of the EU

Figure S43. Generalized impulse response functions (GIRFs) of extra-EU cheese export prices after a negative one-standard-error shock to the Netherlands' cheese production

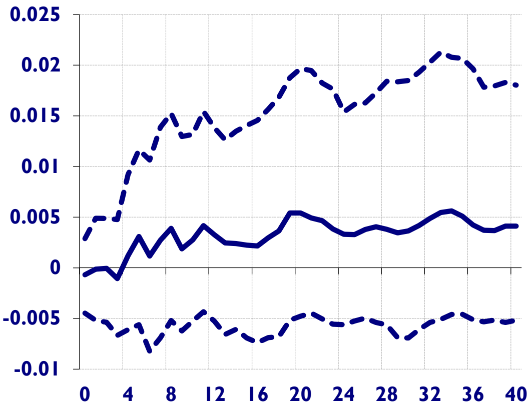

(a) Ireland

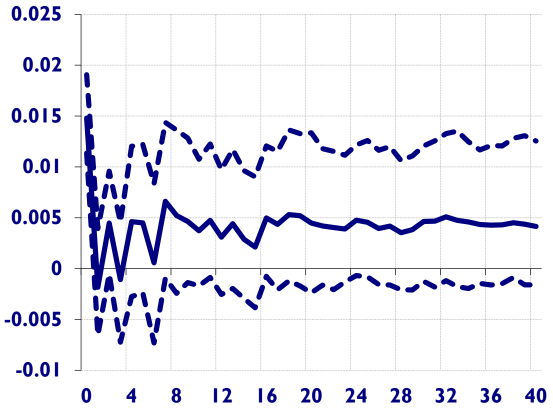

(b) The Netherlands

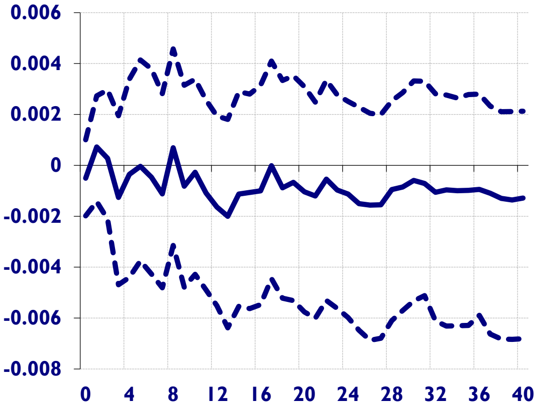

(c) Italy

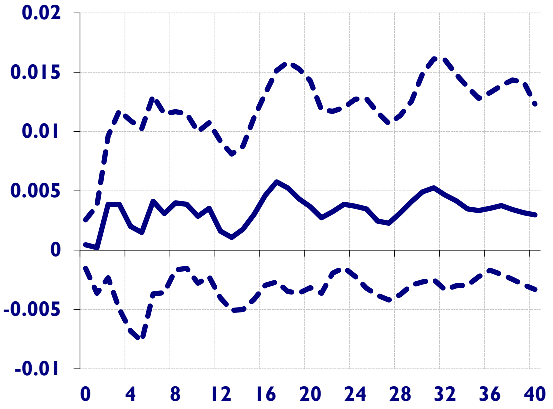

(d) Germany

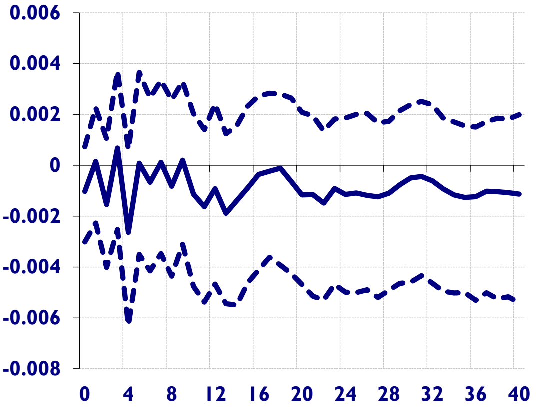

(e) France

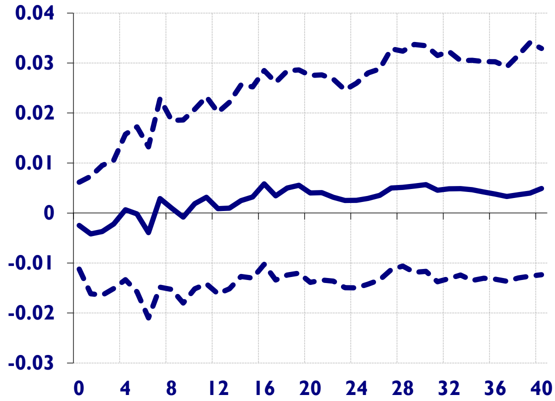

(f) The United Kingdom

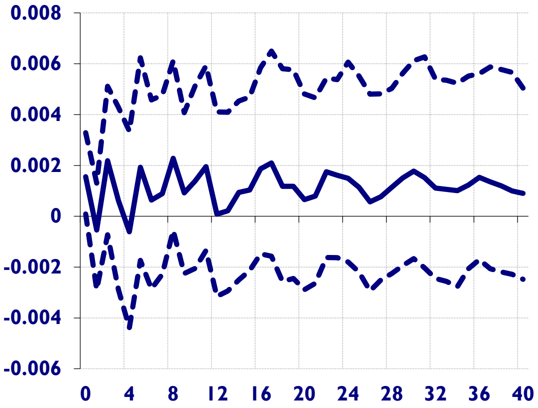

(g) The Rest of the EU

Figure S44. Generalized impulse response functions (GIRFs) of extra-EU cheese export prices after a positive one-standard-error shock to the Netherlands' extra EU cheese export price

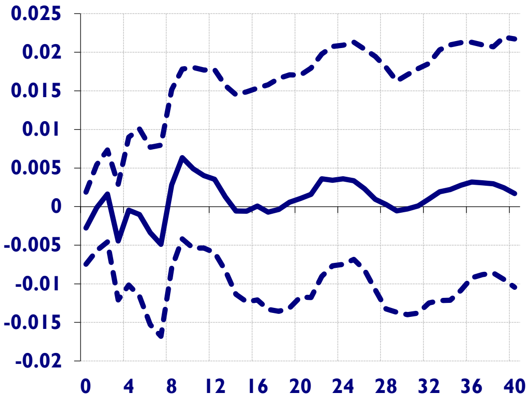

(a) Ireland

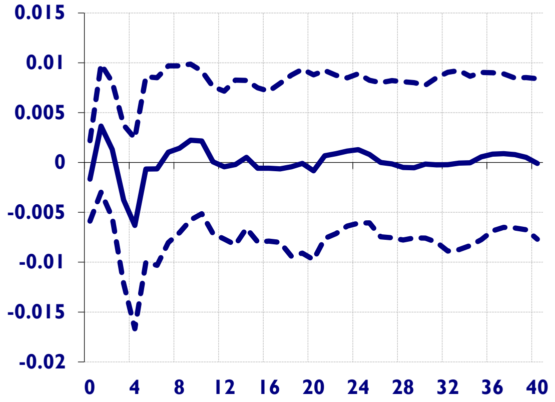

(b) The Netherlands

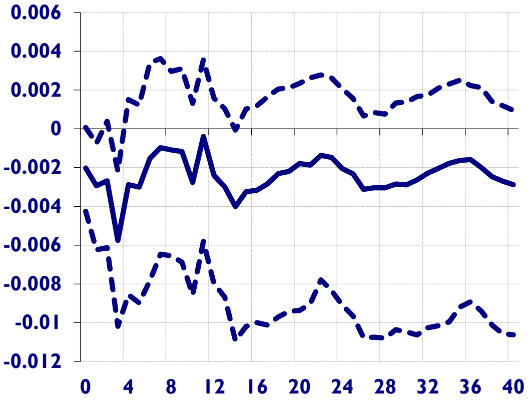

(c) Italy

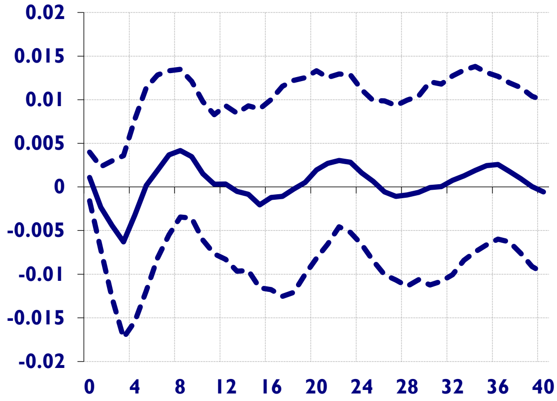

(d) Germany

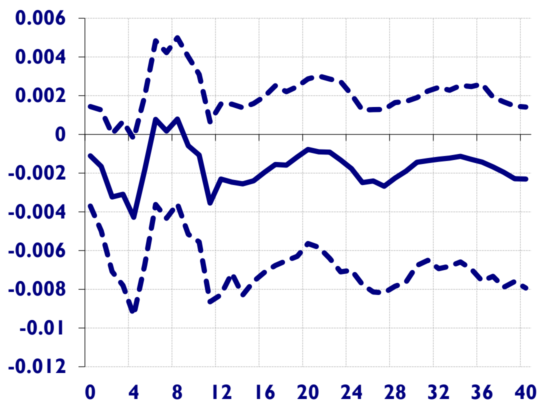

(e) France

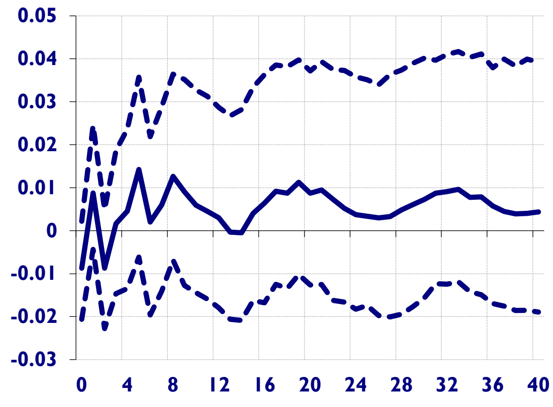

(f) The United Kingdom

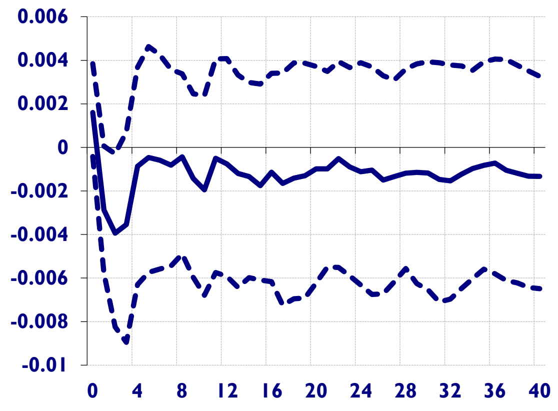

(g) The Rest of the EU

Figure S45. Generalized impulse response functions (GIRFs) of extra-EU cheese export prices after a positive one-standard-error shock to the Netherlands' HICP

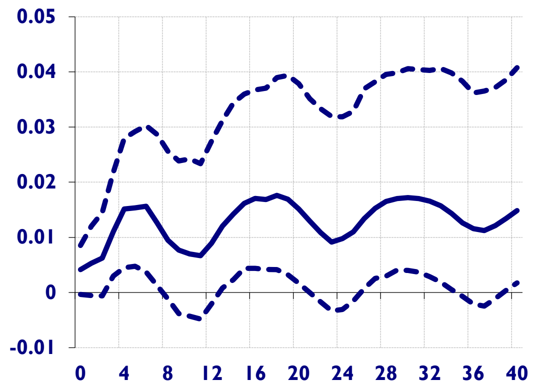

(a) Ireland

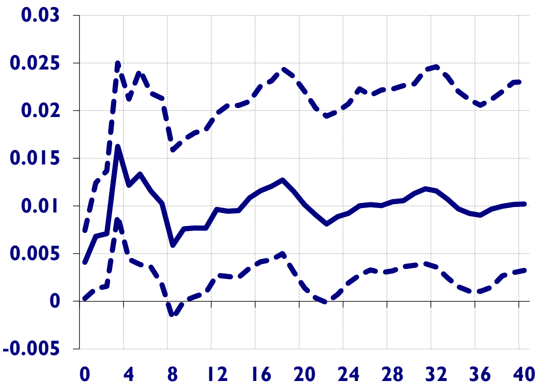

(b) The Netherlands

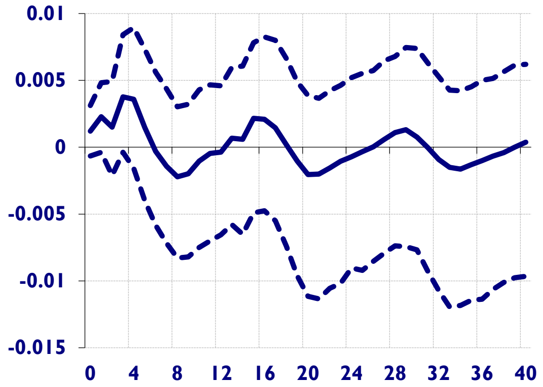

(c) Italy

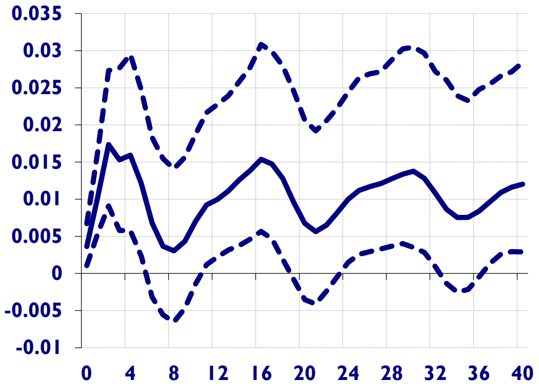

(d) Germany

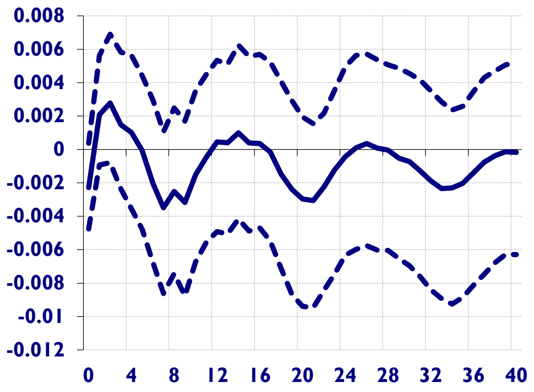

(e) France

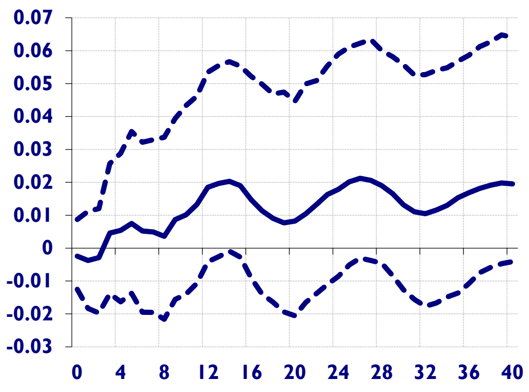

(f) The United Kingdom

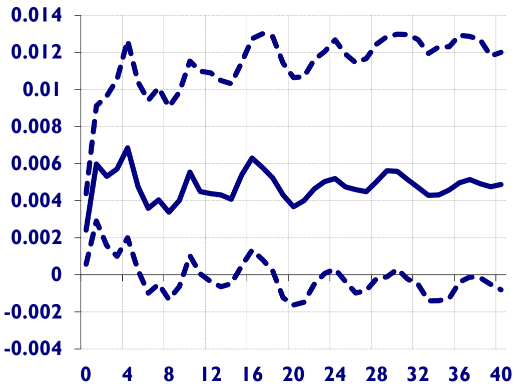

(g) The Rest of the EU

Figure S46. Generalized impulse response functions (GIRFs) of extra-EU cheese export prices after a positive one-standard-error shock to the Netherlands' raw milk price

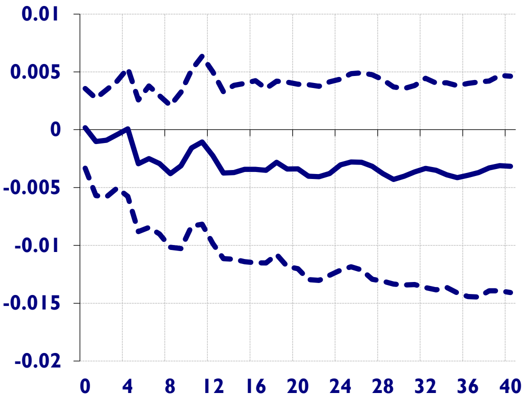

(a) Ireland

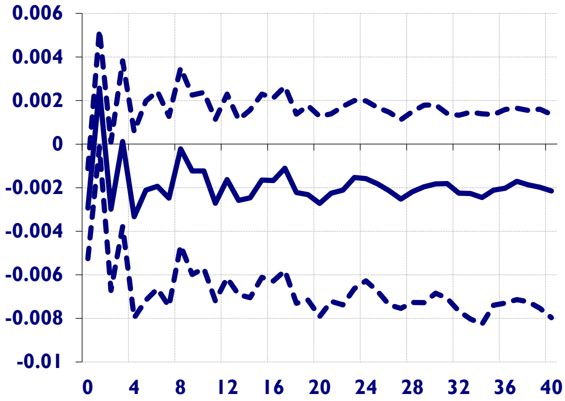

(b) The Netherlands

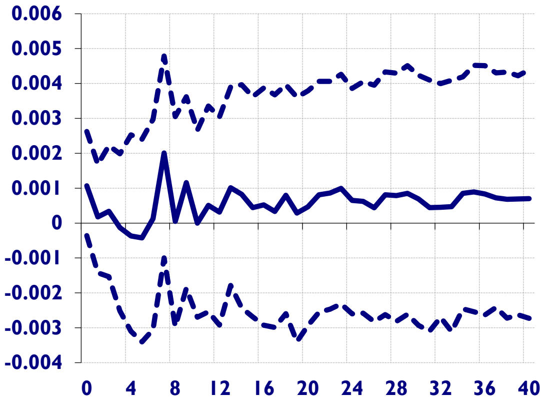

(c) Italy

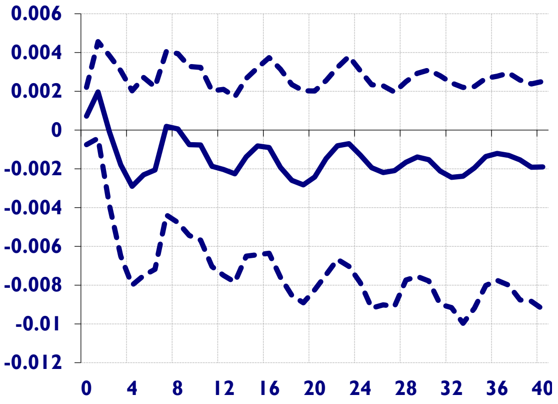

(d) Germany

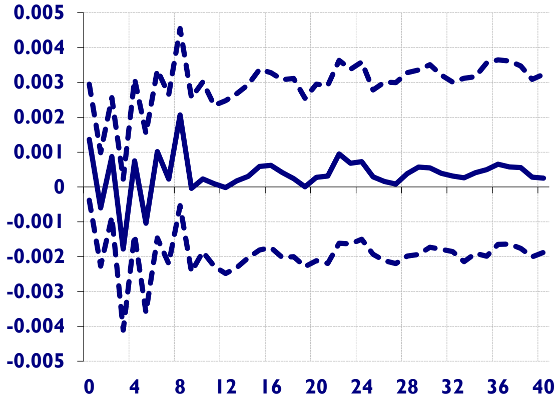

(e) France

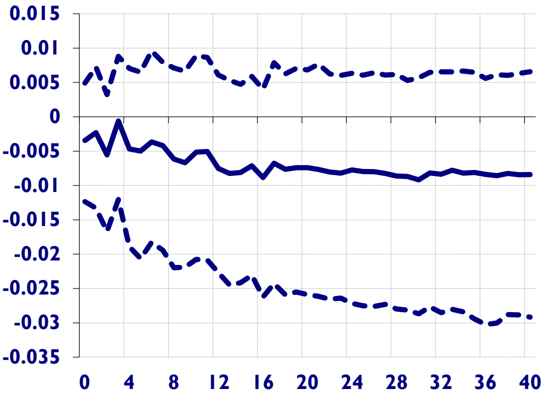

(f) The United Kingdom

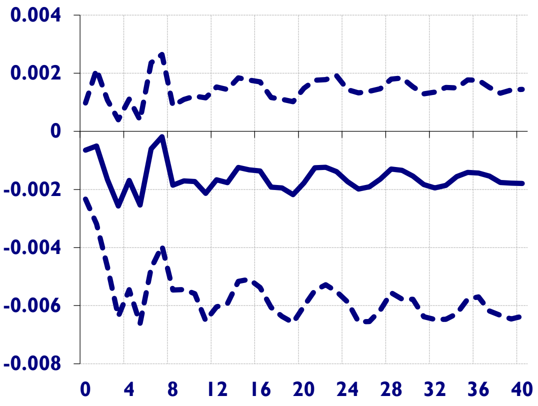

(g) The Rest of the EU

Figure S47. Generalized impulse response functions (GIRFs) of extra-EU cheese export prices after a negative one-standard-error shock to REU's cheese production

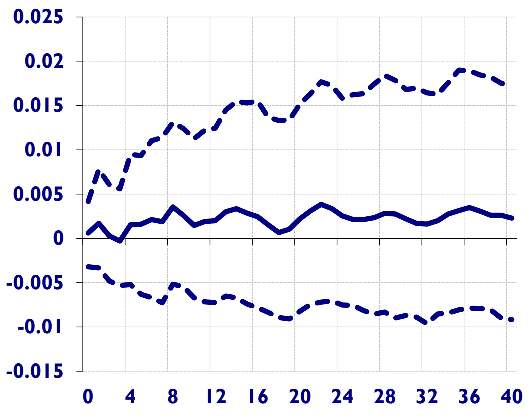

(a) Ireland

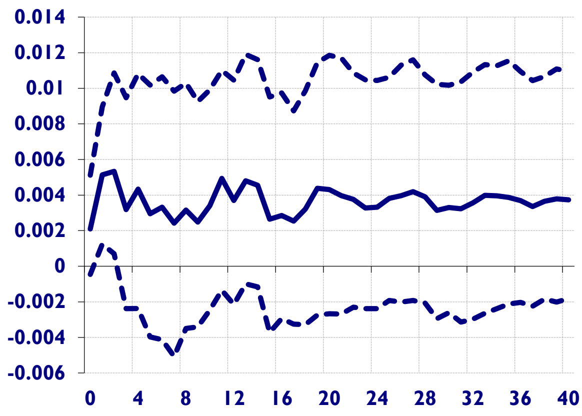

(b) The Netherlands

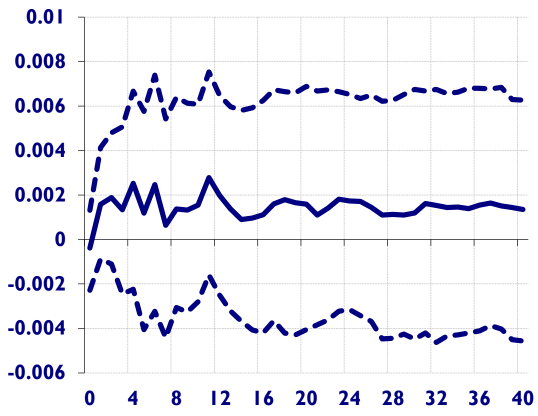

(c) Italy

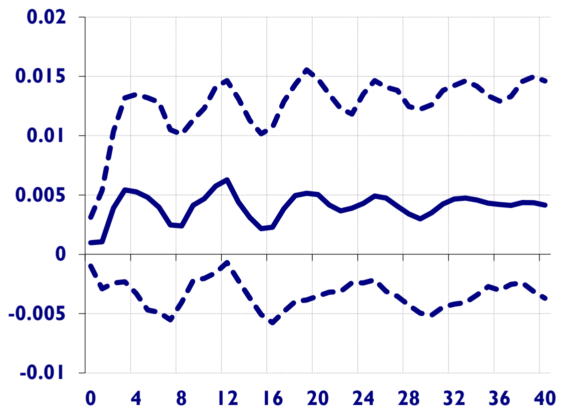

(d) Germany

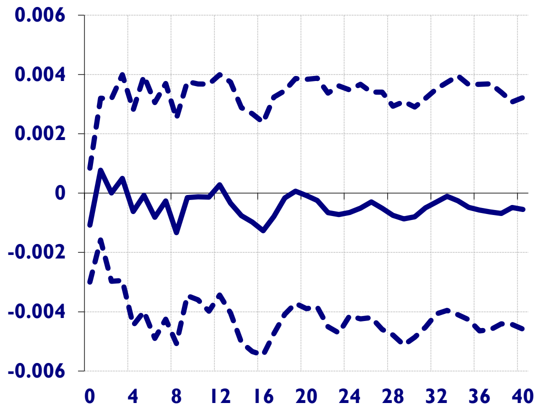

(e) France

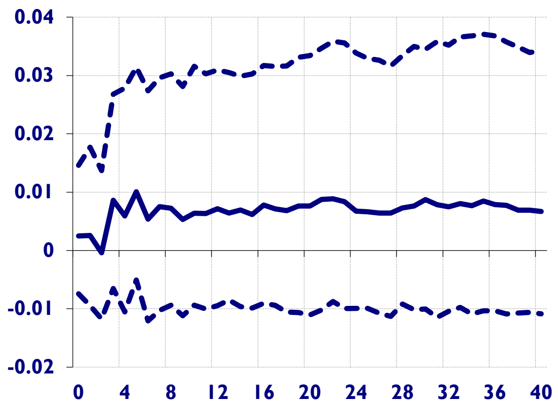

(f) The United Kingdom

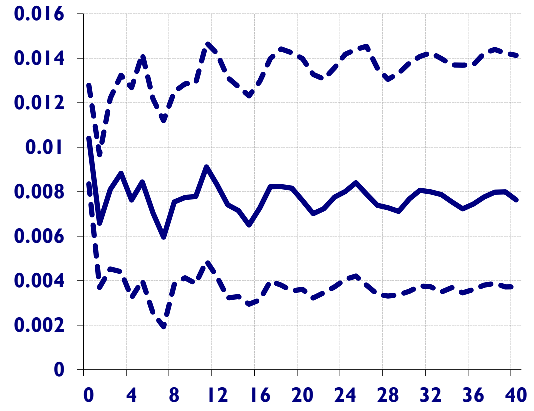

(g) The Rest of the EU

Figure S48. Generalized impulse response functions (GIRFs) of extra-EU cheese export prices after a positive one-standard-error shock to REU's extra EU cheese export price

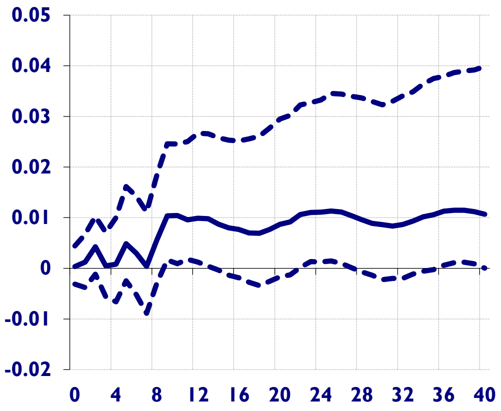

(a) Ireland

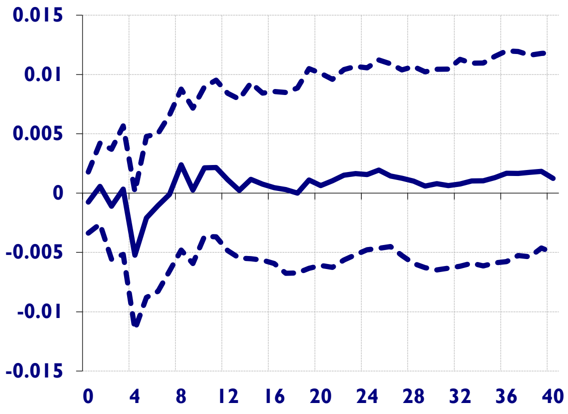

(b) The Netherlands

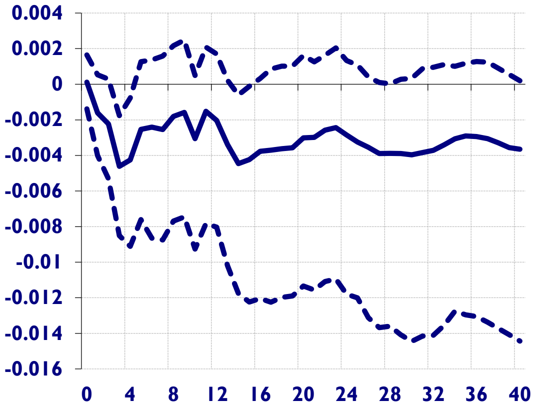

(c) Italy

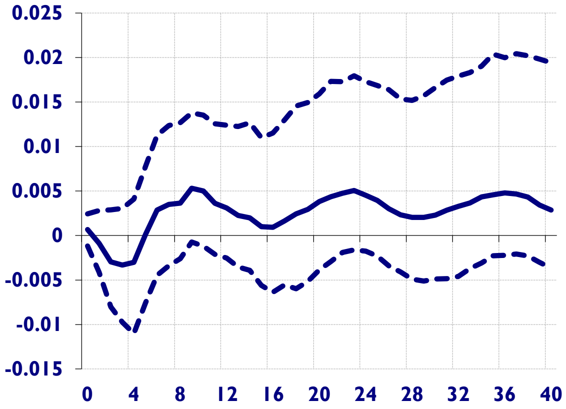

(d) Germany

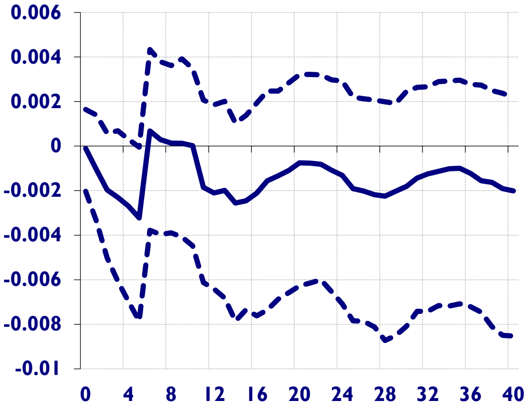

(e) France

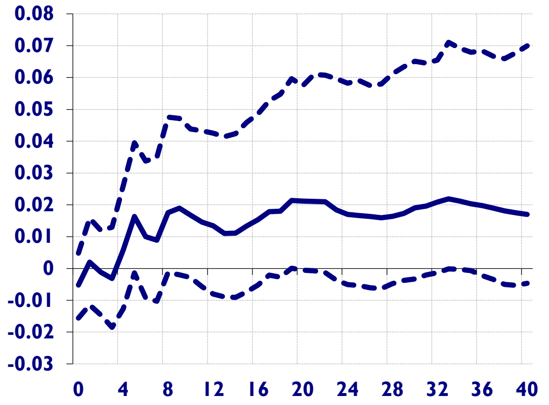

(f) The United Kingdom

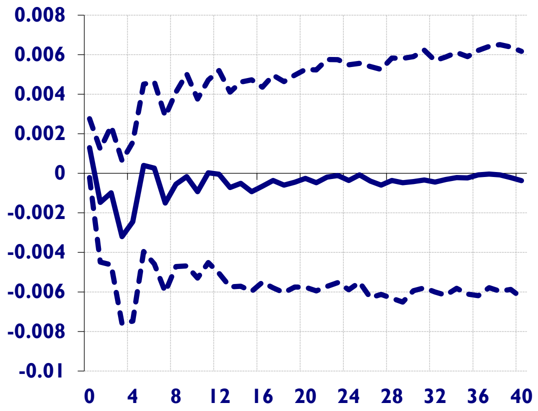

(g) The Rest of the EU

Figure S49. Generalized impulse response functions (GIRFs) of extra-EU cheese export prices after a positive one-standard-error shock to REU's HICP

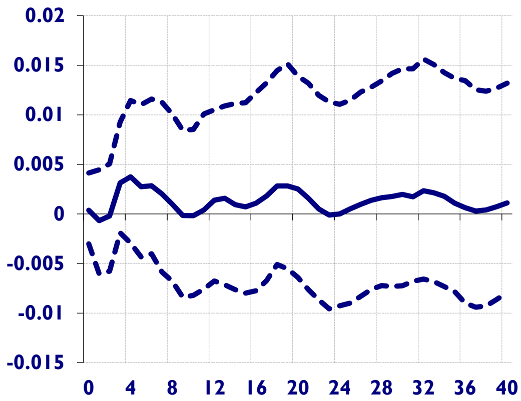

(a) Ireland

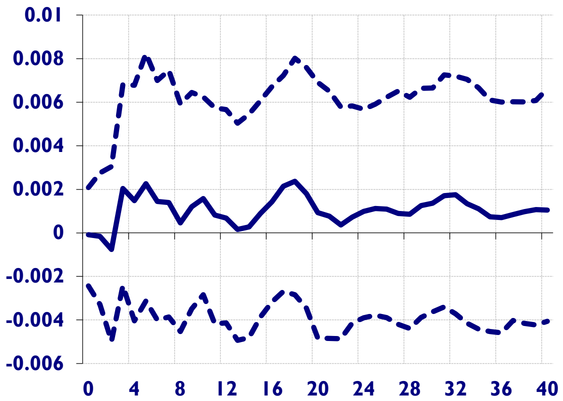

(b) The Netherlands

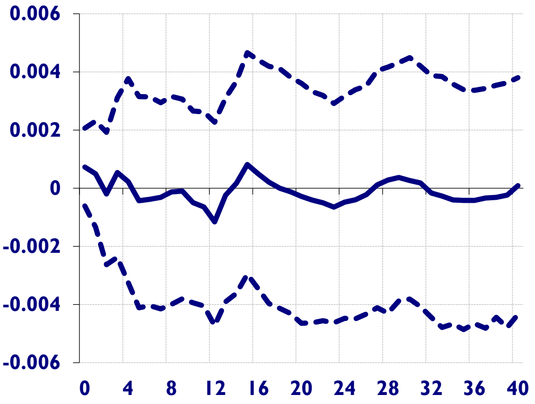

(c) Italy

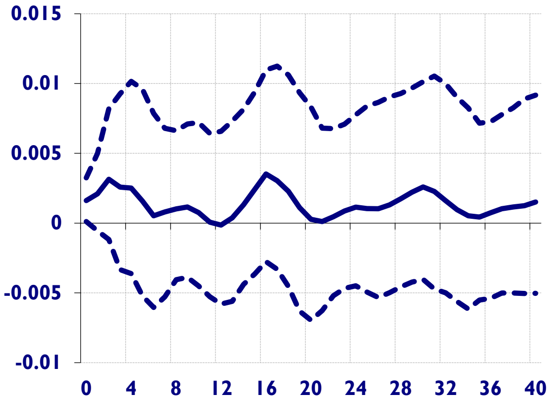

(d) Germany

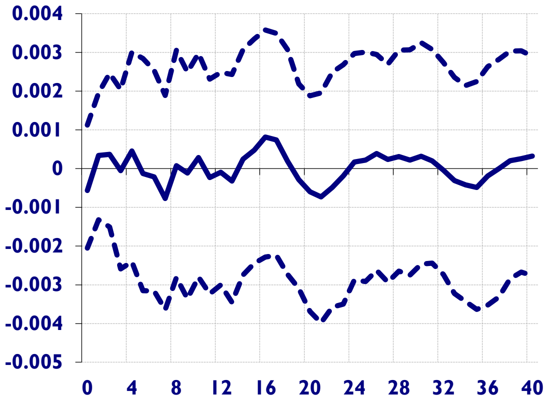

(e) France

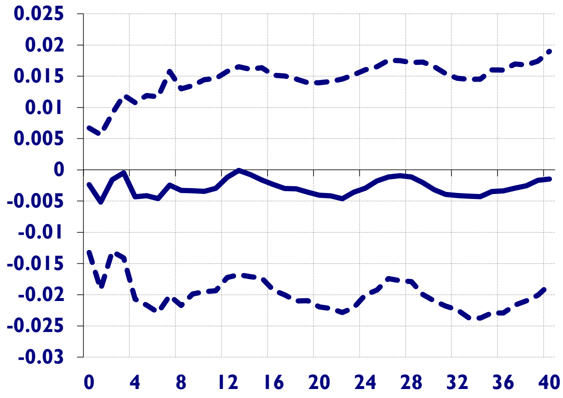

(f) The United Kingdom

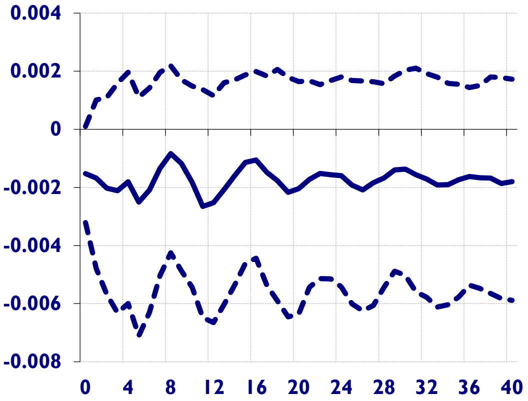

(g) The Rest of the EU

Figure S50. Generalized impulse response functions (GIRFs) of extra-EU cheese export prices after a positive one-standard-error shock to REU's raw milk price

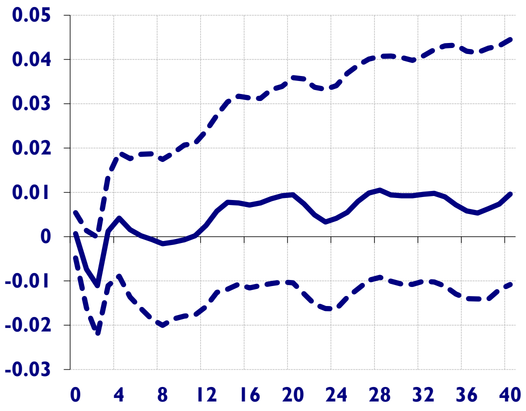

(a) Ireland

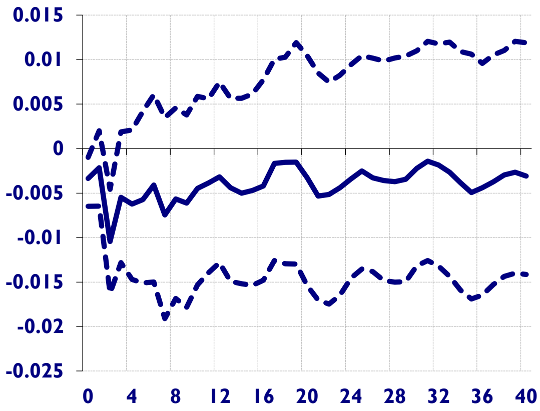

(b) The Netherlands

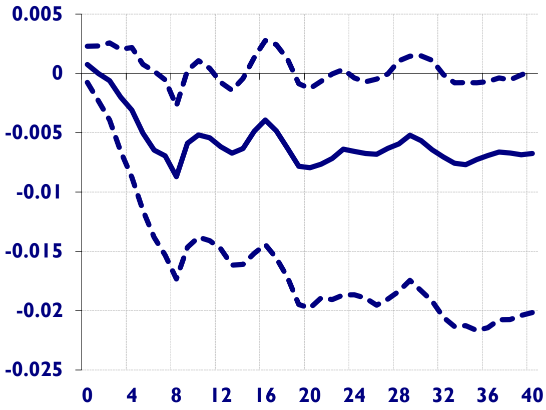

(c) Italy

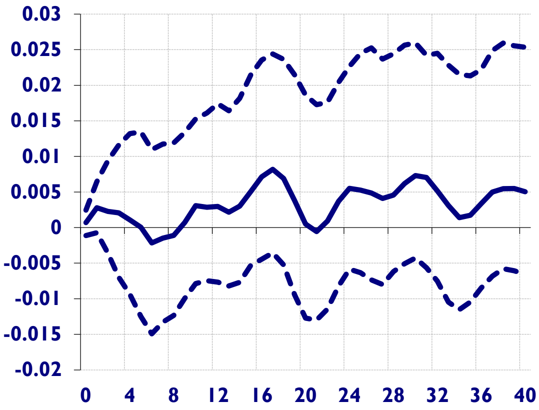

(d) Germany

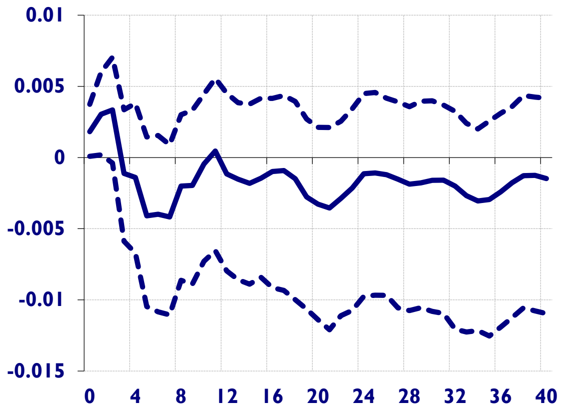

(e) France

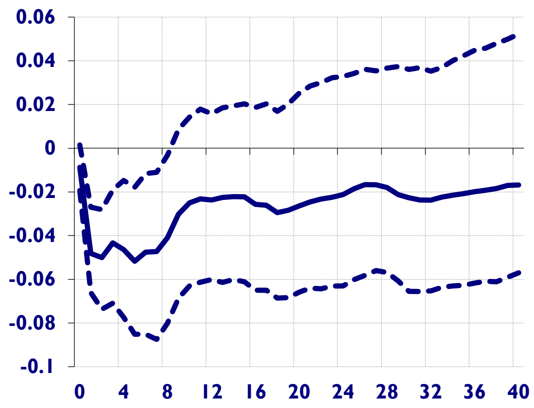

(f) The United Kingdom

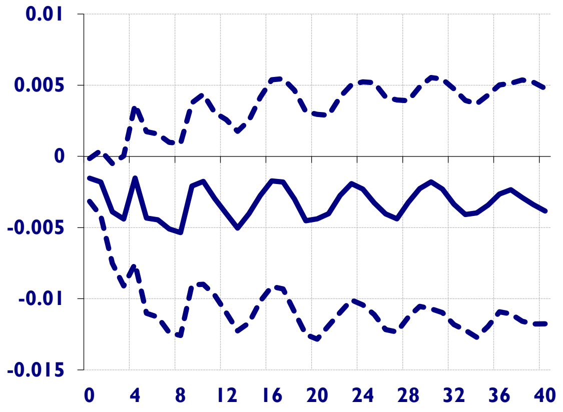

(g) The Rest of the EU

Figure S51. Generalized impulse response functions (GIRFs) of extra-EU cheese export prices after a negative one-standard-error shock to the UK's cheese production

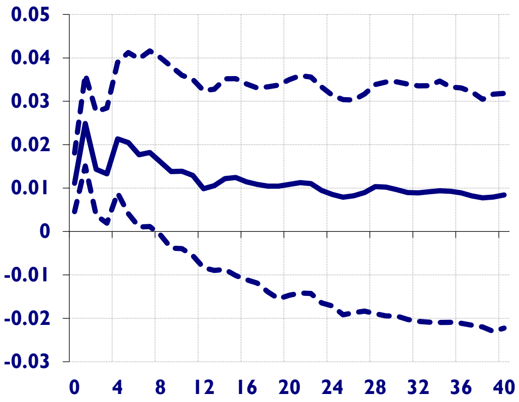

(a) Ireland

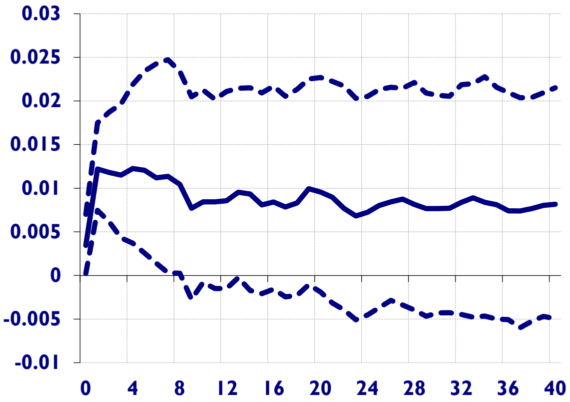

(b) The Netherlands

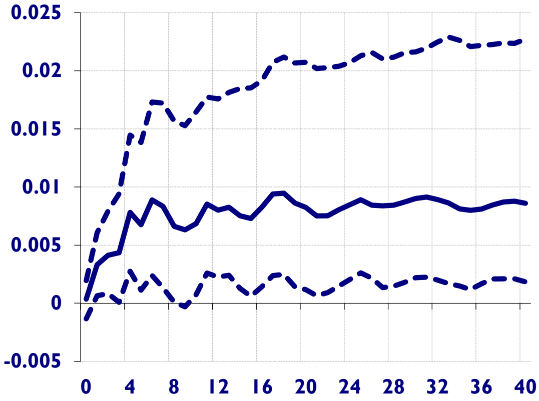

(c) Italy

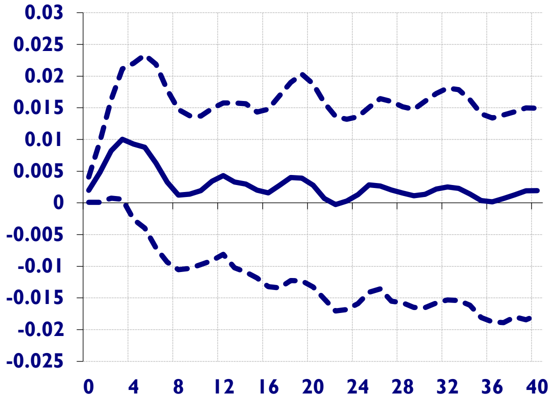

(d) Germany

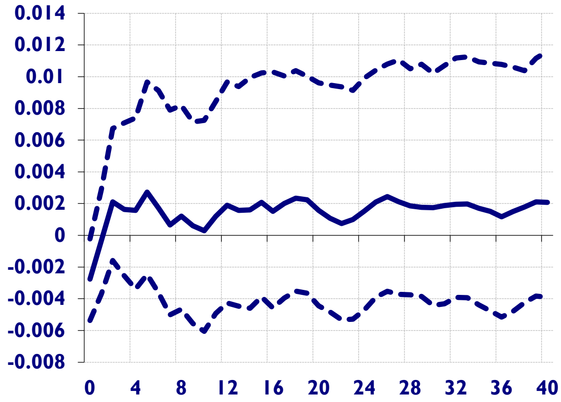

(e) France

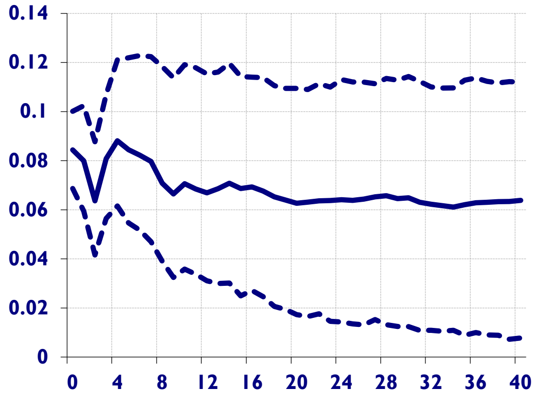

(f) The United Kingdom

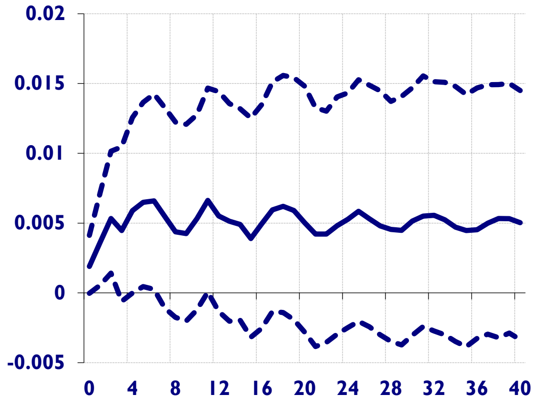

(g) The Rest of the EU

Figure S52. Generalized impulse response functions (GIRFs) of extra-EU cheese export prices after a positive one-standard-error shock to the UK's extra EU cheese export price

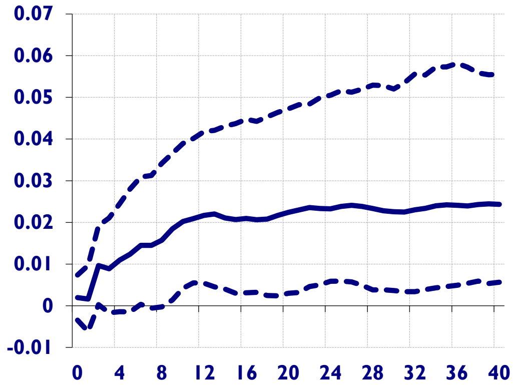

(a) Ireland

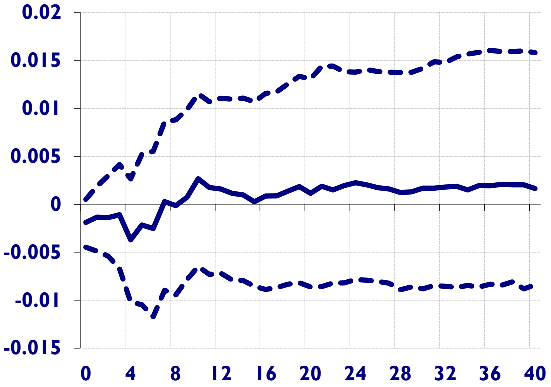

(b) The Netherlands

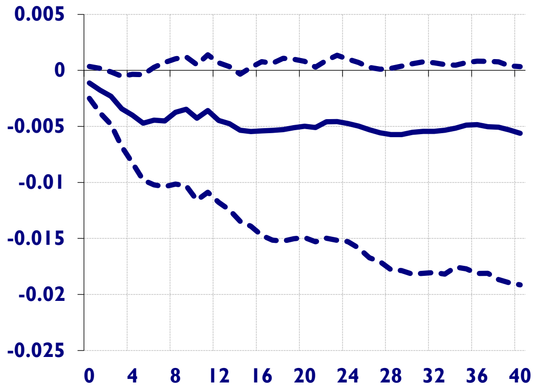

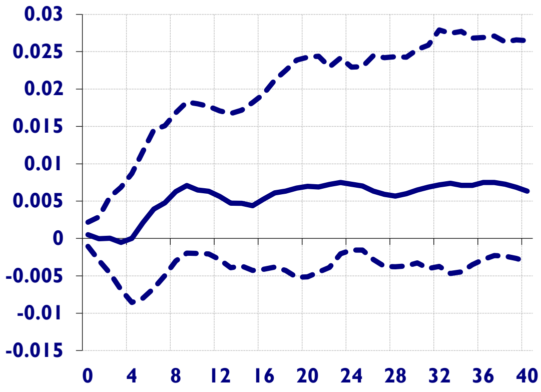

(c) Italy

(d) Germany

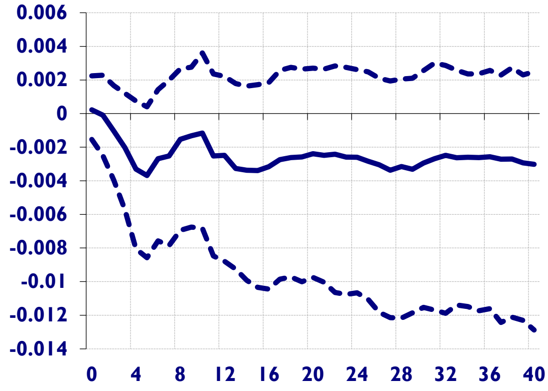

(e) France

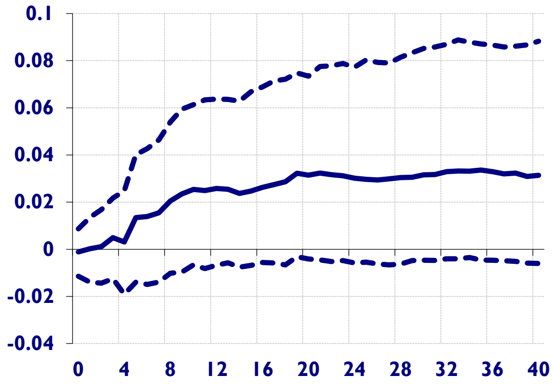

(f) The United Kingdom

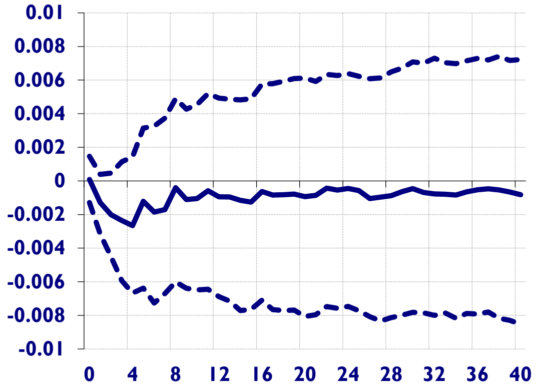

(g) The Rest of the EU

Figure S53. Generalized impulse response functions (GIRFs) of extra-EU cheese export prices after a positive one-standard-error shock to the UK's HICP

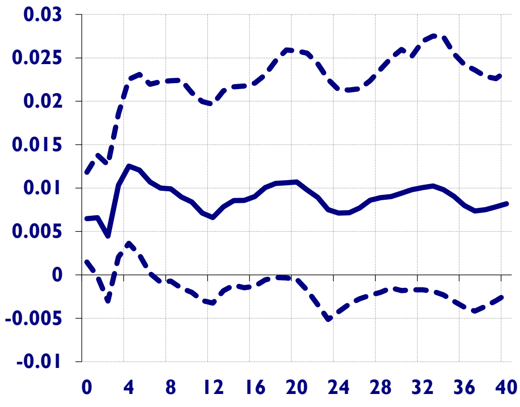

(a) Ireland

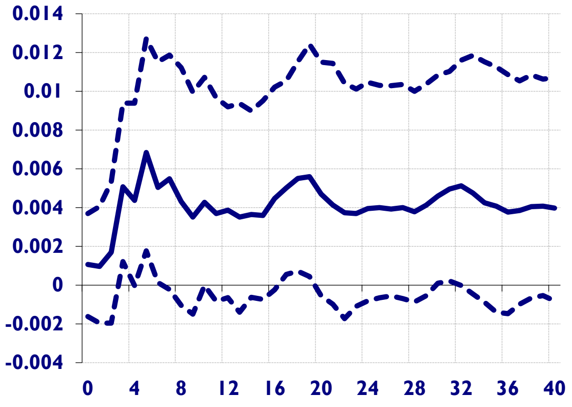

(b) The Netherlands

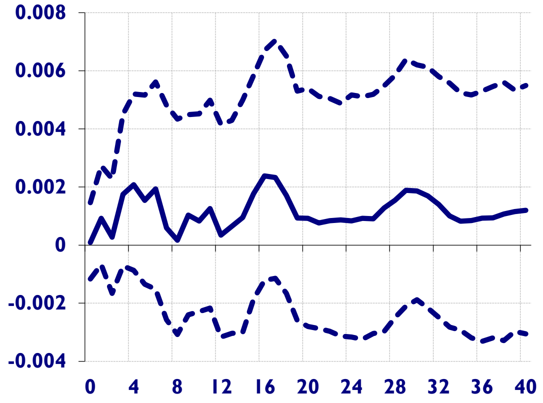

(c) Italy

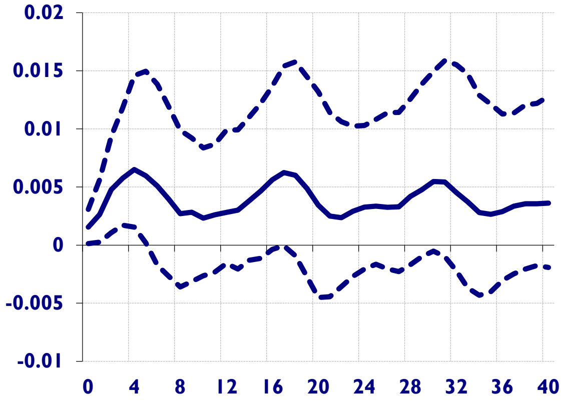

(d) Germany

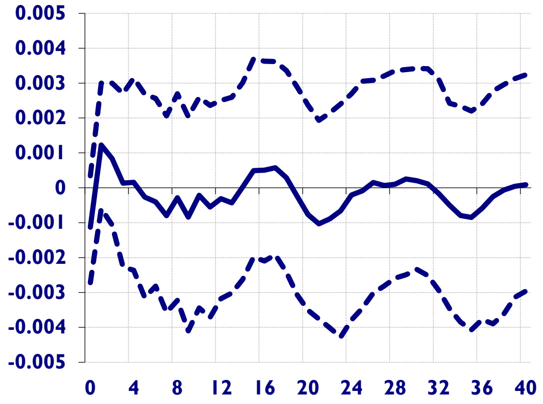

(e) France

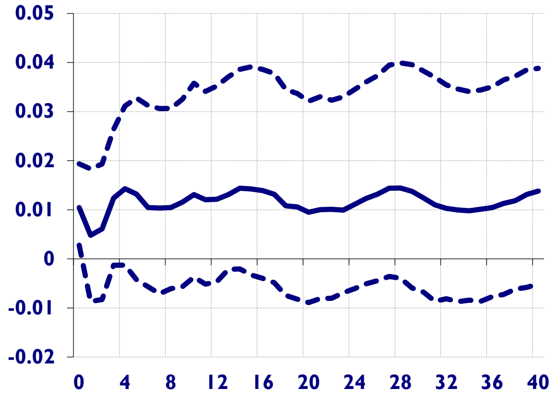

(f) The United Kingdom

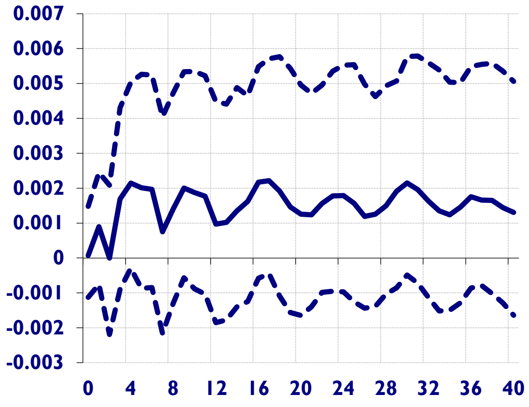

(g) The Rest of the EU

Figure S54. Generalized impulse response functions (GIRFs) of extra-EU cheese export prices after a positive one-standard-error shock to the UK's raw milk price

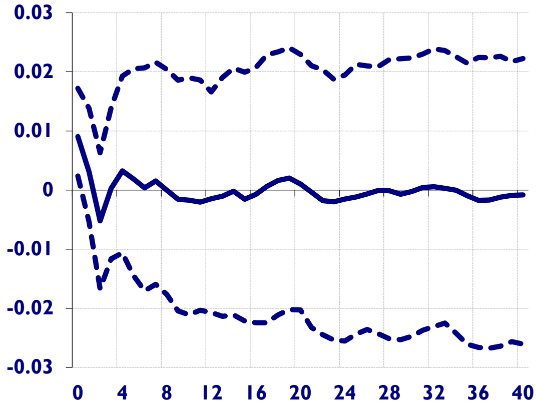

(a) Ireland

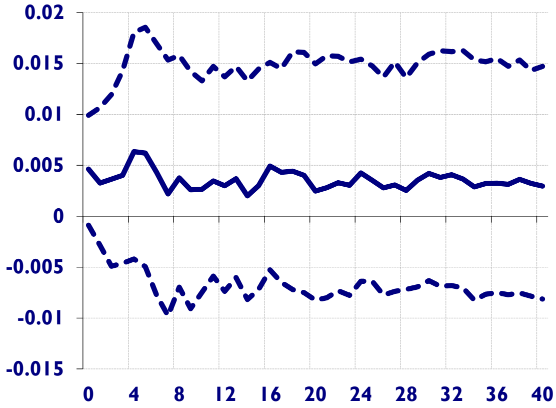

(b) The Netherlands

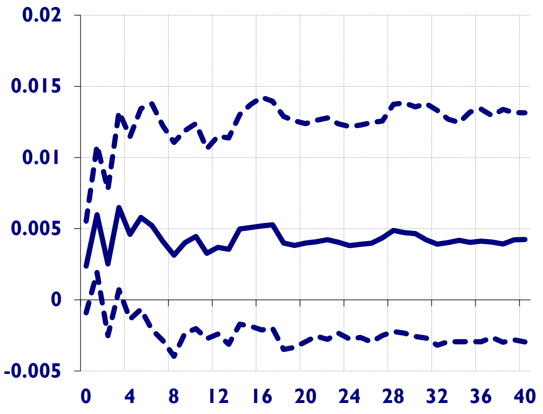

(c) Italy

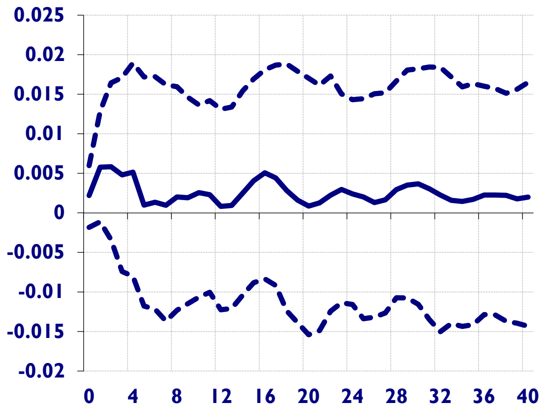

(d) Germany

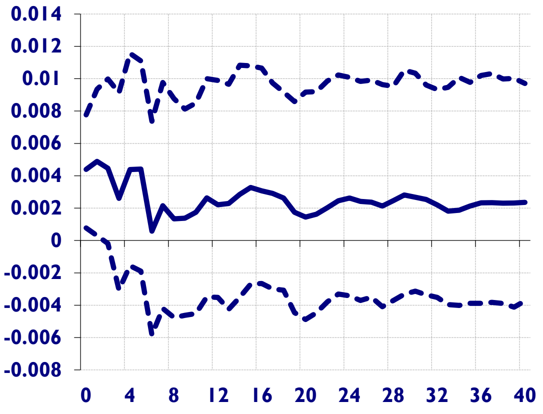

(e) France

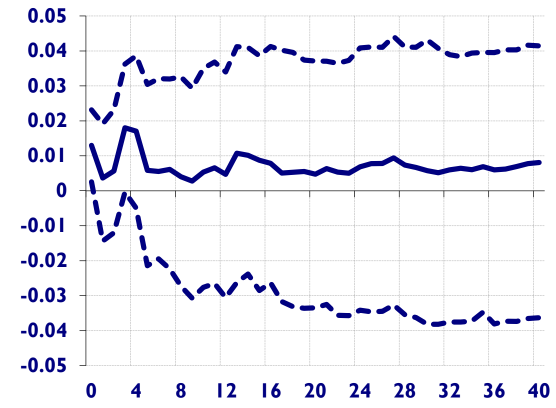

(f) The United Kingdom

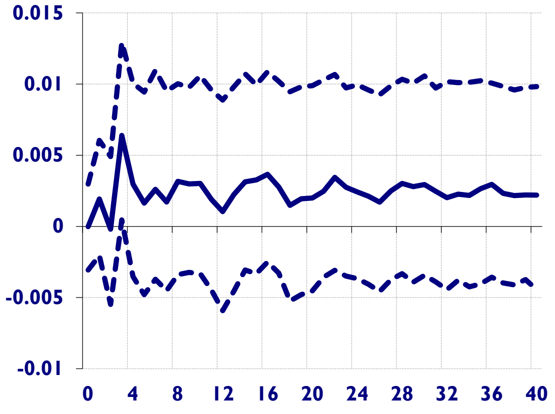

(g) The Rest of the EU

Figure S55. Generalized impulse response functions (GIRFs) of extra-EU cheese export prices after a positive one-standard-error shock to crude oil price
